# Naphthoquinone-derived tridentate Ru(ii) and Os(ii) organometallics with exceptional cytotoxicity: synthesis, characterization, stability in aqueous solution and biological *in vitro* evaluation

**DOI:** 10.1039/d5dt01649e

**Published:** 2026-01-20

**Authors:** Alexander Rosner, Heiko Geisler, Michaela Hejl, Mathias Gradl, Anton A. Legin, Alexander Prado-Roller, Michael A. Jakupec, Petra Heffeter, Walter Berger, Bernhard K. Keppler, Wolfgang Kandioller

**Affiliations:** a Institute of Inorganic Chemistry, Faculty of Chemistry, University of Vienna Währinger Str. 42 1090 Vienna Austria wolfgang.kandioller@univie.ac.at; b Vienna Doctoral School in Chemistry (DoSChem), Faculty of Chemistry, University of Vienna Währinger Str. 42 1090 Vienna Austria; c Research Cluster “Translational Cancer Therapy Research” Währinger Str. 42 1090 Vienna Austria; d Center for Cancer Research and Comprehensive Cancer Center, Medical University of Vienna Austria

## Abstract

In this work, a panel of twelve ruthenium(ii) and osmium(ii) derived *N*,*O*,*O*-tridentate complexes (1a–2f) with a variation of longer, branched and unbranched alkyl substituents was synthesized and characterized *via* NMR, HRMS, elemental analysis and X-ray diffraction analysis. Resilience to dissociation in biologically relevant solution was determined over 72 hours, revealing most stable complexes to derive from naphthoquinones bearing *tert*-butyl- and *neopentyl*-substituents. Osmium derived complexes were found to be generally more inert than their ruthenium counterparts. Cytotoxicity was examined, revealing IC_50_ values in the nanomolar to lower micromolar range for derivatives 1a–2f in three human cancer lines and a typical pattern of selectivity for SW480 cells. Cellular accumulation correlated with *in vitro* cytotoxicity; however, longer and branched substituents did not improve the cellular accumulation. Cell cycle experiments showed consistent cell cycle inhibition in both SW480 and CH1/PA-1 cells for ruthenium-based compounds only. Indolamin-2,3-dioxygenase 1 (IDO1) inhibition assays in SKOV3 cells revealed significant inhibitory potential of Ru-Ethyl, in clear distinction to other ruthenium and osmium complexes.

## Abstract 2

A panel of organometallic *N*,*O*,*O*-tridentate complexes bearing different alkyl substituents is presented. The impact on stability in aqueous systems, cytotoxicity, cellular accumulation, cell cycle and IDO inhibition is discussed.

## Graphical abstract

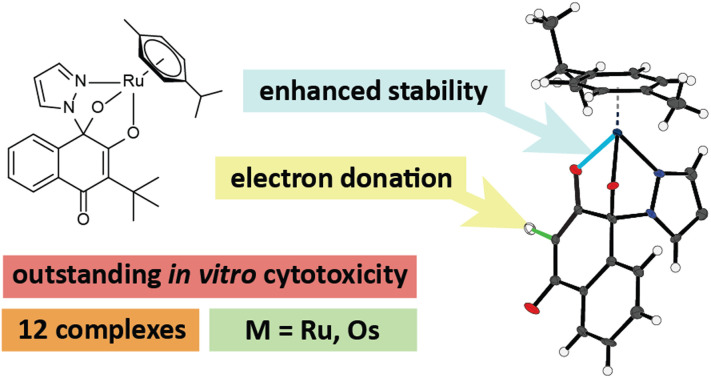

## Introduction

Transition metal-based therapeutics have been clinically used for centuries.^1^ Moving on from established platinum drugs, ongoing research strives for divergent modes of anticancer activity. Thus, novel methods for cancer treatment could be established and current therapeutic challenges overcome.

**Fig. 1:**
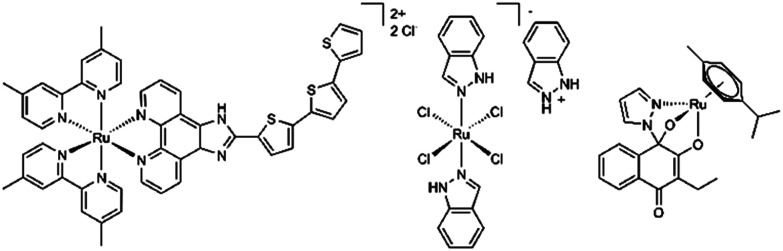
TLD-1433 (left), BOLD-100 (middle) and Ru-ethyl (right).

Certain ruthenium-based derivatives have been investigated for their impact on cancer cell metabolism and already entered clinical trials.^2^ TLD-1433 (Fig. 1), a ruthenium(ii) based complex acts *via* photodynamic activation of oxygen and is used for treatment of light accessible tissue. It was developed by McFarland *et al.* and has been examined in several clinical trials for treatment of bladder cancer, with current recruitment for an upcoming phase II study.^3–6^ For BOLD-100 (Fig. 1), investigations on modes of anticancer activity are driven by clinical research, *in vivo* and *in vitro* studies.^7^ Mode-of-action studies have revealed multimodal anticancer activity exerted *via* DNA damage, stress induction in the endoplasmic reticulum (ER), ribosomal interactions and modulation of GRP78.^8^ Recent *in vitro* studies have revealed alterations in lipid metabolism to result in acquired resistance to BOLD-100. Furthermore, glucose deprivation showed enhanced vulnerability of cells towards this “anti-Warburg drug”. These findings might allow strategic targeting of therapeutic challenges, to advance BOLD-100 in cancer therapy.^9,10^

Research on ruthenium and osmium derived agents often strives for the success of phototherapeutic agents such as TLD-1433, utilizing strongly coordinating *N*,*N*- or *C*,*N*-chelating ligands for an inert complex formation.^11,12^ These metallodrugs are generally favored by structurally simple design and high efficacy. Regardless, photodynamic therapy (PDT) has certain limitations, such as light penetration issues, poor pharmacokinetics and the narrow range of treatable cancer types.^13^ Recent approaches examined enhancement of bioaccumulation *via* insertion of targeting moieties or implementation of nanoparticle formulations.^11^ Modulation of photodynamic properties was studied *via* chromophore insertion to ligand scaffolds.^14^

Most other metallodrugs rely on an alterable coordination sphere. For many novel agents, formation of aquated species or metal center interaction with biological nucleophiles plays a vital role in tumor uptake and intracellular modes of action.^15,16^ Organometallic ruthenium(ii) and osmium(ii) piano stool complexes have been investigated for many years. Providing a highly variable coordination sphere, they allow fine-tuning of ADMET properties. Choice of ligand composition, selection of donor atoms and denticity are decisive for the pharmacokinetic and pharmacodynamic properties as well as solution chemistry of these metallodrugs.^17,18^ An η^6^-bound arene stabilizes the metal center and enhances lipophilicity, whereas remaining coordination sites are usually occupied by mono- or bidentate ligands.^19^ Ligands of low stability (*e.g.* Cl, Br) act as leaving groups, allowing the formation of aquated species available for interaction with molecular targets.^20^ Modes of anticancer activity tend to deviate from platinum derived agents. Dyson *et al.* reported RAPTA-C as a first in class agent in 2001.^21^ A PTA (1,3,5-triaza-7-phosphaadamantane) auxiliary and two chlorido ligands are coordinated to the *para*-cymene ruthenium scaffold. Despite its structural simplicity, RAPTA-C follows complex aquation mechanisms and acts *via* multiple modes of anticancer activity.^22^ Lack of activity *in vitro* and on primary tumours was opposed by high tolerability and growth inhibition on metastases *in vivo*.^23^ RAPTA-C and follow-up works have exemplified the difficulty to establish structure activity relationships (SAR) for piano stool complexes. Changes in ligand composition greatly impact modes of action and biodistribution, which to this day holds a main challenge in design and development of metallodrugs.^24,25^

The importance of ligand exchange kinetics was also demonstrated on the DNA-binding ruthenium agent RM175. Metal center variation from ruthenium to osmium led to a loss of anti-metastatic activity *in vivo*.^26^ Osmium analogs are generally known for their higher inertness towards aquation, which consequently alters pharmacokinetic profiles.^26–28^

Most piano stool complexes bearing tridentate ligand architectures were assumed to be too stable for this form of anticancer therapy. Although stable complex formation is a desirable approach for drug administration and delivery, these species have shown diminished activity and find scarce reports in literature.^29,30^ This indicates that activation of certain coordination sites for target interaction is necessary and again highlights the importance of ligand exchange kinetics.^30^ In contrast to these findings, a novel tridentate species with outstanding *in vitro* potency has been reported recently by our group.^31^ Dimeric metal precursors, bioactive naphthoquinones and 1,2-diazoles formed *in situ* a highly stable N,O,O-architecture (Fig. 1). Formation of a hemiaminal group is presumably mediated *via* the metal center, making the 1*H*-diazole proton susceptible to abstraction by a weak base. The resulting complexes show high stability in aqueous media and exceptional *in vitro* cytotoxicity with a certain selectivity towards rather chemo-resistant cell lines.^31^ Thus, possibly facilitating administration and body distribution as an intact species, their solution chemistry implies eventual cleavage of metal oxygen bonds. Previous works postulated initiation *via* breakage of the least stable ruthenium–oxygen bond (Ru–O_2_). Subsequent aquation of the metal center is followed by unresolved processes of ligand dissociation. In absence of biological targets, eventual formation of dimeric pyrazolo bridged dimers indicates naphthoquinone release. However, this might only account for the specific experimental setup in buffered aqueous solution and could strongly differ in a biological setting.^32^ Derivatives with alkyl substituents have shown increasing stability in aqueous solution, but nevertheless highest cytotoxicity *in vitro*. Geisler *et al.* proposed a positive inductive effect of aliphatic groups on the naphthoquinone π system to result in stronger coordination of the tridentate ligand.^32^ Evidently, highest stability in aqueous medium was observed for derivatives bearing methyl, ethyl and cyclohexyl residues. We hypothesized that further enhancement of complex stability could be achieved by variations of alkyl substituents. Thus, a broader scope of complexes with diverging alkyl substituents could provide proof for this hypothesis and further allow to elucidate impact on physicochemical properties. Moreover, the most stable of these complexes show highest cytotoxicity *in vitro*. Ru-Ethyl (Fig. 1) exerts exceptionally high impact on partially chemoresistant colon carcinoma cells (SW480: IC_50_ concentrations at 0.046 ± 0.007 µM over 96 hours), while other cell lines seem to be less sensitive (CH1/PA-1, A549).^32^ To best of our knowledge, this *in vitro* selectivity pattern can be attributed to all complexes of this compound class. Still, biological properties remain widely unexplored. DNA interaction or generation of reactive oxygen species (ROS) were already ruled out in previous works. No conclusive results on targets of free naphthoquinones (*e.g.* the redox enzyme NQO1) were found in previous works.^32,33^ The naphthoquinone scaffold is associated with a broad variety of anticancer activities. However, target specificity is often limited to certain derivatives.^34^ Indoleamine-2,3-dioxygenase 1 (IDO1) has been identified as the target of naphthoquinone derived anticancer agents.^35,36^ While metabolizing tryptophan in the kynurenine pathway, IDO1 holds the regulatory role of maintaining immune tolerance. In cancer, aberration of its healthy functions leads to immunosuppression, crucially impacting mechanisms of tumor immune evasion.^37^ Within this work we want to provide further insight into the impact of chemical modifications on the naphthoquinone scaffold on physicochemical properties and changes in biological behavior.

Moreover, alteration of cell cycle progression and IDO1 as a potential target were examined.

## Results and discussion

### Synthetic procedures

**Fig. 2:**
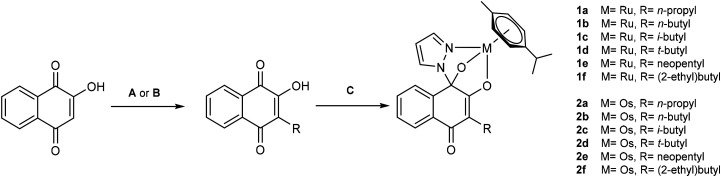
Synthetic scheme for synthesis of ligands a–f and complexes 1a–2f. Procedure **A** (lawsone, aldehyde RHO, l-proline, Hantzsch ester; one-pot reaction under microwave irradiation) yielded ligands a, b, c and f. Procedure **B** (lawsone, acid RCOOH, peroxodisulfate, silver nitrate; heating in acetonitrile/water) provided access to ligands d and f. Procedure **C** (ligand a–f, pyrazole, metal dimer 1 or 2, triethylamine; one-pot reaction under microwave irradiation) for synthesis of complexes 1a–2f.

1,4-Hydroxynaphthoquinones were modified with electron inducing (aliphatic) substituents to enhance the coordination motif's inertness towards dissociation. Overall, a small compound library of twelve naphthoquinone derived tridentate ruthenium and osmium organometallics (1a–f, 2a–f) and their corresponding ligands (a–f) was synthesized. Incorporation of a range of longer and branched alkyl chains (C_3_–C_6_) on naphthoquinone ligands required different synthetic strategies (Fig. 2).

Synthesis of naphthoquinone ligands a, b, c and f was adapted from organocatalytic procedures published by Ramachary, D. B. *et al*.^38^ In a one-pot microwave reaction, aliphatic aldehydes were used to alkylate Lawsone *via* a l-proline catalyzed mechanism. For introduction of longer and branched alkyl chains, reaction temperatures up to 78 °C were sufficient. Hantzsch ester served as a mild reductant, to yield desired alkyl naphthoquinones as their corresponding bases. Acidic workup and purification *via* column chromatography led to desired ligands in acceptable to excellent yields (36–85%).

This approach provided access to a variety of alkyl naphthoquinones, however there are limitations. Longer and branched aldehydes (*e.g.* pivalaldehyde) have shown diminished or no reactivity. Furthermore, this procedure conserves the aldehyde carbon as a methylene moiety on the substituent. Thus, it provides C–C bond formation of 4-hydroxynaphthoquinone to primary carbon atoms. Geisler *et al.* utilized procedures for the access of secondary carbon substituents (*e.g.* cyclohexyl),^32^ whereas tertiary substituents are accessible *via* radical induced activation of carboxylic acids.^39^ Synthesis of naphthoquinone ligands d and e was adapted from literature, using procedures established by Olímpio da Silva, A. *et al.*^39^ Pivalic acid or *tert*-butylacetic acid were treated with peroxodisulfate and catalytic amounts of silver nitrate, in presence of lawsone. The desired naphthoquinones were obtained in acceptable yields (25–27%).

Ruthenium or osmium based derivatives were synthesized in analogy to published procedures.^32^ In a one-pot reaction, organometallic precursor 1 or 2 were treated with pyrazole, triethylamine and the respective naphthoquinone derivatives a–f under microwave irradiation. Subsequent purification *via* column chromatography and precipitation yielded desired complexes 1a–2f as yellow powders in good to excellent yields (52–89%) (Fig. 2).

### Characterization

**Fig. 3:**
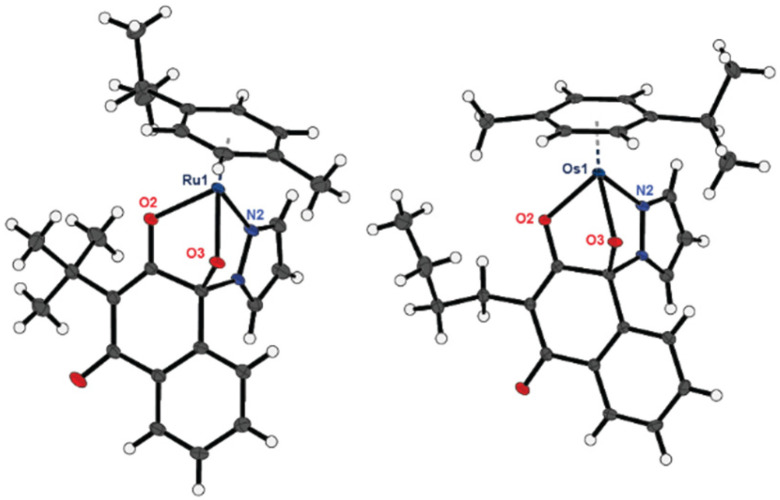
Crystal structures and numbering of coordination motifs of complexes 1d (left) and 2b (right).

Formation of desired complexes was confirmed *via* standard analytical procedures such as ^1^H- and ^13^C-NMR (see Fig. S1–S18), respectively the according 2D-NMR spectra and HRMS spectra (see Fig. S19–S30). High elemental purity was confirmed *via* elemental analysis. In addition, the hygroscopic behavior of most complexes was proven by the measured elemental O-values, which is characteristic for this class of organometallics and in line with similar derivatives of previous works.^32,33^ Furthermore, single crystals of the complexes 1a–2f were investigated *via* X-ray crystallography (Fig. 3).

^1^H-NMR shifts of the aromatic protons of pyrazole and naphthoquinone moieties confirmed complex formation.

Aromatic protons of the *p*-cymene ligand separate into four distinct signals due to complex formation. In addition, ^13^C-NMR spectra confirmed the hemiaminal structure, displayed as a characteristic peak with low intensity around 95 ppm. HRMS spectra were measured in positive mode and show distinctive ruthenium and osmium isotope distribution patterns. Both [M + H^+^] and [M + Na^+^] adducts were detected.

Single crystals were grown from diethyl ether/DCM/toluene *via* vapor diffusion (see Fig. S31–S42 and Tables S1–S12). Compounds 1a, 1c–2a, 2d–2f crystallized in the triclinic space group *P*1̄, whereas 1b, 2b, 2c show a monoclinic crystal system of the space group *P*2_1_/*n*. Oxygen O2 was found to form the longest bonds to respective metal centers for all derivatives, except 1a (Table 1). The latter has a longer bond from nitrogen N2 to the ruthenium center.

**Table 1:** Selected bond lengths of the coordination motifs of 1a–2f

Derivative	Bond length/Å		
	N2–M	O2–M	O3–M
1a	2.1041(10)	2.1015(8)	2.0560(8)
1b	2.1028(16)	2.1200(15)	2.0431(13)
1c	2.088(2)	2.114(2)	2.0503(19)
1d	2.1030(12)	2.1159(11)	2.0551(10)
1e	2.090(2)	2.0944(18)	2.0568(17)
1f	2.081(2)	2.0924(18)	2.0618(17)
2a	2.103(2)	2.1123(19)	2.0643(17)
2b	2.0994(17)	2.1329(14)	2.0545(13)
2c	2.101(5)	2.104(4)	2.059(4)
2d	2.086(5)	2.112(4)	2.041(4)
2e	2.101(4)	2.096(4)	2.071(3)
2f	2.097(5)	2.109(5)	2.066(4)
Ru-Ethyl ^32^	2.097(2)	2.116(2)	2.049(2)

Solubilities of (2-ethyl)butyl derivatives 1f and 2f were found insufficient and therefore these compounds were excluded from biological evaluations and studies on physicochemical properties. Osmium derivative 2d showed slow precipitation over time and was therefore excluded from UHPLC based stability measurements. Dimethyl sulfoxide was used as solubilizer for stability and biological studies.

^1^H-NMR studies showed sufficient stability of the complexes and no adduct formation or decomposition was observed.

### Behavior in aqueous Systems

**Fig. 4:**
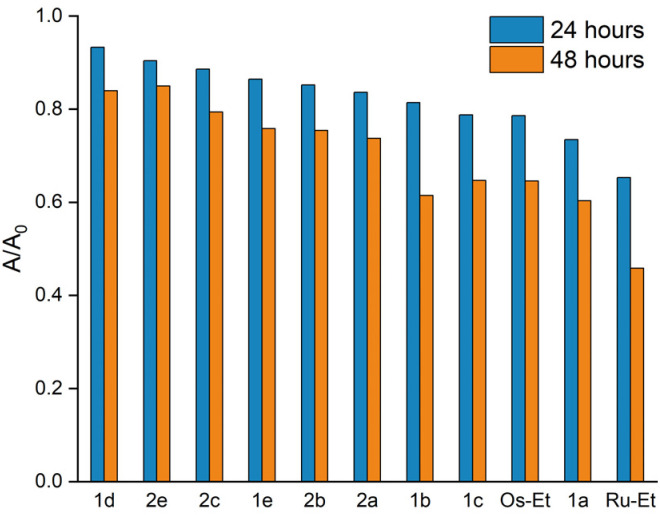
Stability order of complexes in 1% DMSO/PBS over 24 and 48 hours.

Aqueous stability of complexes 1a–e, 2a–e, Ru-Ethyl and Os-Ethyl was determined *via* incubation in phosphate buffered saline (pH 7.4)/1% DMSO at 20 °C over 48 hours. Solutions were analyzed *via* UHPLC at 0, 1, 2, 3, 4, 5, 24 and 48 hours (see Fig. S43–S56). Peak areas of intact complex species were compared to areas at time point 0 (*A*/*A*_0_ = area at given timepoint/area at timepoint 0). In analogy to previous publications,^31^ osmium derivatives 2a–f show higher inertness than their ruthenium counterparts 1a–f. All measured complexes showed higher stability than the ethyl analogs, according to the following ranking: Ru-Ethyl < 1a ∼ 1b < 1c < 1e < 1d, respectively Os-Ethyl < 2a ∼ 2b < 2c < 2e (Fig. 4). Concluding from these results, complex stability is favored by longer and branched alkyl chains in the order ethyl < propyl ∼ butyl < isobutyl < neopentyl < tertbutyl. Despite the less inert ruthenium center of tertbutyl complex 1d, it shows higher stability than osmium based neopentyl derivative 2e over 24 hours and similar stability over 48 hours. A tertbutyl group should have the highest inductive effect on the naphthoquinone π system and thus could be reason for extraordinary stability of 1d. Osmium analog 2d slowly precipitated from solution, therefore a comparative value cannot be provided. These results strongly support interpretations and estimations of Geisler *et.al.*^32^ Concluding from X-ray diffraction data, enhanced complex stability is not reflected in relevant changes of bond lengths of the tridentate coordination motif (Table 1).

### Cytotoxicity

Cytotoxicity of complexes 1a–2e and ligands a–e was determined in three human carcinoma cell lines, namely A549 (non-small cell lung cancer), SW480 (colon cancer) and CH1/PA-1 (ovarian teratocarcinoma) over 96 hours. For IDO1 inhibition assays, IC_50_ concentrations in SKOV3 (ovarian adenocarcinoma) were determined for Ru-Ethyl, Os-Ethyl, 1a, 2a, 1c and 2c (Table 2, Fig. S60 and S61). Both ruthenium and osmium derived complexes show distinctly higher cytotoxicity in A549 and SW480 than in the otherwise highly chemosensitive CH1/PA-1 cell line. In the latter, IC_50_ values are in a similar range as those of the free ligands a–e, which exert only moderate to poor cytotoxicity overall. Clearly, highest impact was found on SW480 cells, which aligns with results of previous publications on this class of compounds.^31,32^ Metal center variation does not display a strong impact on the observed IC_50_ values. Overall, ruthenium and osmium derived complexes of same ligand composition show comparable values here. Usually, ruthenium- and osmium-based analogs are somewhat divergent in their biological properties, mainly due to the slower ligand exchange kinetics of osmium.^17^ In SW480 cells, IC_50_ values are lowest for complexes deriving from *n*-propyl and *n*-butyl naphthoquinone ligands a and b. Notably, higher values were obtained for complexes of branched derivatives c, d and e. With exception of 2d, their IC_50_ values are in the low µM range. Although all complexes of this work are highly cytotoxic in SW480 cells, Ru-Ethyl ranks among the most potent ruthenium based anticancer agents in literature and was at least one order of magnitude more active in SW480, slightly more active in A549 and similarly active in PA-1/CH1 cells compared to the presented compounds 1a–2e.

**Table 2:** *In vitro* anticancer activity of ligands a–e and the corresponding Ru(ii) and Os(ii) complexes (1a–2e)

Compound	Metal center	IC_50_ values^a^/µM			
		A549	SW480	CH1/PA-1	SKOV3
a		50 ± 8	39 ± 1	67 ± 3	n.d.
1a	Ru^II^	2.3 ± 0.7	0.26 ± 0.07	46 ± 8	11.7 ± 4.4
2a	Os^II^	1.8 ± 0.5	0.23 ± 0.03	64 ± 9	18.7 ± 2.6
b		73 ± 3	49 ± 8	41 ± 5	n.d.
1b	Ru^II^	3.0 ± 0.3	0.58 ± 0.01	26 ± 1	n.d.
2b	Os^II^	1.6 ± 0.1	0.36 ± 0.08	43 ± 4	n.d.
c		71 ± 2	56 ± 3	44 ± 3	n.d.
1c	Ru^II^	9.6 ± 2.0	1.7 ± 0.2	32 ± 3	54.6 ± 3.2
2c	Os^II^	6.4 ± 0.7	1.2 ± 0.1	55 ± 8	>50
d		89 ± 9	51 ± 5	33 ± 2	n.d.
1d	Ru^II^	8.0 ± 1.6	2.0 ± 0.4	14 ± 2	n.d.
2d	Os^II^	2.4 ± 0.2	0.63 ± 0.05	29 ± 3	n.d.
e		45 ± 1	43 ± 1	35 ± 1	n.d.
1e	Ru^II^	11 ± 1	1.5 ± 0.3	30.0 ± 0.3	n.d.
2e	Os^II^	7.3 ± 1.1	1.6 ± 0.3	36 ± 3	n.d.
Ru-Ethyl ^32^	Ru^II^	0.76 ± 0.14	0.046 ± 0.007	62 ± 5	4.1 ± 1.7
Os-Ethyl ^40^	Os^II^	0.88 ± 0.19	0.072 ± 0.003	61 ± 4	12.7 ± 2.7
Cisplatin ^41^	Pt^II^	6.2 ± 1.2	3.3 ± 0.2	0.077 ± 0.006	4.6 ± 1.8
BOLD-100 ^42^	Ru^III^	156 ± 11	88 ± 19	62 ± 9	n.d.

a IC_50_ concentrations were determined in human carcinoma cell lines *via* MTT assay over 96 hours. n.d. = not determined.

### Cellular accumulation

**Fig. 5:**
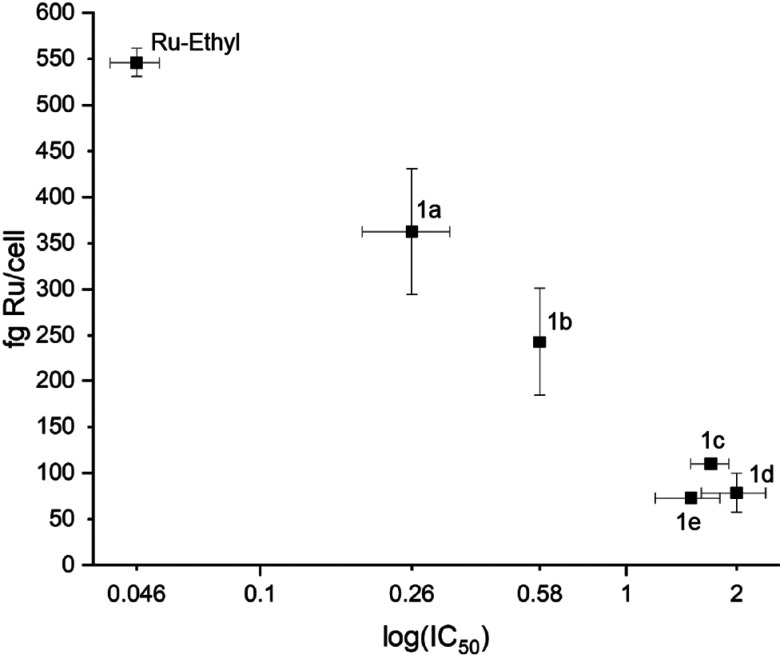
Correlation of cellular accumulation and cytotoxicity.

Cellular accumulation was determined for ruthenium derivatives 1a–1e and Ru-Ethyl. SW480 cells were treated with respective complexes, lysed and analyzed for their ruthenium content *via* ICP-MS (see Fig. 5 and Table S13). Obtained values range from a maximum of 546 ± 15 ng per cell for Ru-Ethyl down to 73 ± 6 ng per cell for 1e. Complexes only differ in length and branching of aliphatic residues on the naphthoquinone scaffold. Table 3 provides an overview of substituents and calculated miLog *P* (Molinspiration Log *P* (ref. 43)) values of free ligands, compared to the respective IC_50_ values. Although ligand lipophilicities are not necessarily translatable to the respective metal complexes, we assume a tendency of increasing lipophilicity when it comes to substituent chain extension from two to five carbon units. As can be seen in Table 3, accumulation of 1a–1e does not confirm this expectation. Complexes with longer and branched aliphatic substituents show lower cellular accumulation and consequently higher IC_50_ values than Ru-Ethyl. This trend was observed for 1a and 1b and branched derivatives 1c, 1d and 1e (see Fig. 5). This indicates a greater role of the naphthoquinone substituents’ steric demands for cellular accumulation. Previous works showed an opposite trend for arene variation, where more lipophilic arenes correlated with enhanced cellular accumulation.^33^

**Table 3:** Accumulation of ruthenium derivatives 1a–1e and Ru-Ethyl in SW480 cells (solvent: 0.5% DMSO). Values are complemented by substituent types, lipophilicities of free ligands and IC_50_ values of 1a–1e and Ru-Ethyl

Compound	Substituent	Cellular accumulation^a^	Lipophilicity^b^	Cytotoxicity
		fg Ru per cell	miLog *P* ligand	IC_50_/µM
Ru-Ethyl	C_2_H_5_	546 ± 15	2.22	0.046 ± 0.007
1a	C_3_H_7_	362 ± 68	2.61	0.26 ± 0.07
1b	C_4_H_9_	243 ± 59	3.17	0.58 ± 0.01
1c	C_4_H_9_	110 ± 5	2.61	1.70 ± 0.2
1d	C_4_H_9_	78 ± 21	3.01	2.00 ± 0.4
1e	C_5_H_11_	73 ± 6	3.19	1.50 ± 0.3

a Cellular accumulation in SW480 cells upon 2 h of exposure to 50 µM of test substance (solvent: 0.5% DMSO in MEM). Ru was quantified by ICP-MS and recalculated per cell.

b Lipophilicity of ligands was determined from the molecular properties calculator of molinspiration.^43^ Lipophilic properties of complexes presumably correlate to lipophilicities of free ligands.

### Cell cycle

The effects of organoruthenium and organoosmium complexes Ru-Ethyl, 1a, 1c, Os-Ethyl, 2a, 2c on cell cycle distribution were investigated in ovarian teratocarcinoma (PA-1/CH1) and colon carcinoma (SW480) cell lines (Fig. 7). The exposure concentrations were chosen in relation to the cytotoxicity results in both cell lines, with corresponding IC_50_ values as maximum applied concentrations. For comparison, the highest concentration applied in the more sensitive SW480 cells was also applied in the PA-1/CH1 cell line. Consequently, we observed a less pronounced effect in ovarian teratocarcinoma cell line when compared to that in colon cancer cells (Fig. 7). Interestingly, alterations in the cell cycle distribution in both cell lines were much more evident following 24 h exposure to ruthenium-based compounds than to osmium analogues. (In contrast, the 96 h exposure resulted in very similar cytotoxic potency in SW480 and PA-1/CH1 cell lines, as mentioned above.) The strongest cell cycle inhibitory efficacy in both cell lines was shown for the most cytotoxic ethyl-substituted naphthoquinone ruthenium complex (Ru-Ethyl), namely a 14.5% average increase in G2/M phase relative to control in CH1/PA-1 cells (Fig. 7, top left) and a 20.5% average increase in G2/M phase in SW480 cells. Moreover, the arrest in G2/M phase is accompanied by a sharp decrease of cell subpopulation in G1/G0 cell cycle phase (*e.g.* >30% decrease relative to control in SW480 cells, Fig. 7). In contrast to PA-1/CH1, SW480 cells treated with each of ruthenium-based complexes showed a concentration dependent increase in S phase (11–15% in average) parallel to G2/M phase inhibition. Overall, only ruthenium complexes were consistently inhibiting the cell cycle of both CH1/PA-1 and SW480 cells in a concentration dependent manner (Fig. 7); however, based on the known slower kinetics of osmium complexes it cannot be excluded that an impact on the cell cycle can occur at a later time point.

### IDO inhibition assay

**Fig. 6:**
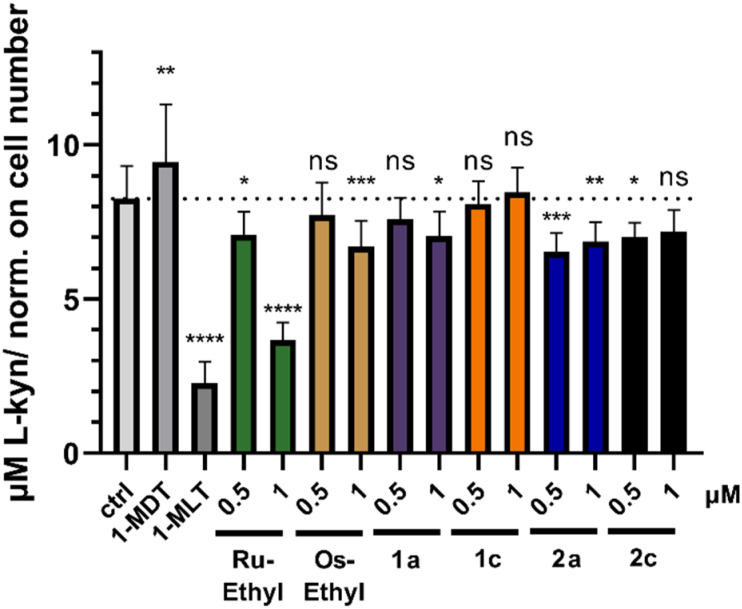
Inhibition of the enzymatic IDO activity of SKOV3 cells *in vitro*. Colorimetric Kyn detection assay in supernatant of SKOV3 cells. Bars depict mean ± SD of 10 replicates normalized to cell number. Significance was calculated in comparison to control.

Based on the recently reported IDO inhibitory potential of some naphthoquinones, we hypothesized that our compound panel could have an impact on this enzyme with immunomodulatory functions. We employed the colorimetric kynurenine (Kyn) assay with 1-MDT (1-methyl-d-tryptophan, *in vivo* positive control) and 1-MLT (1-methyl-l-tryptophan *in vitro* positive control). Indeed, as shown in Fig. 6 especially Ru-Ethyl had distinct and highly significant IDO inhibitory properties in SKOV3 cells whereas its osmium analog and complexes 1a, 1c, 2a, 2c were found distinctly less active. To the best of our knowledge, Ru-Ethyl is the first Ru(ii) complex exhibiting intrinsic IDO inhibition properties.

**Fig. 7:**
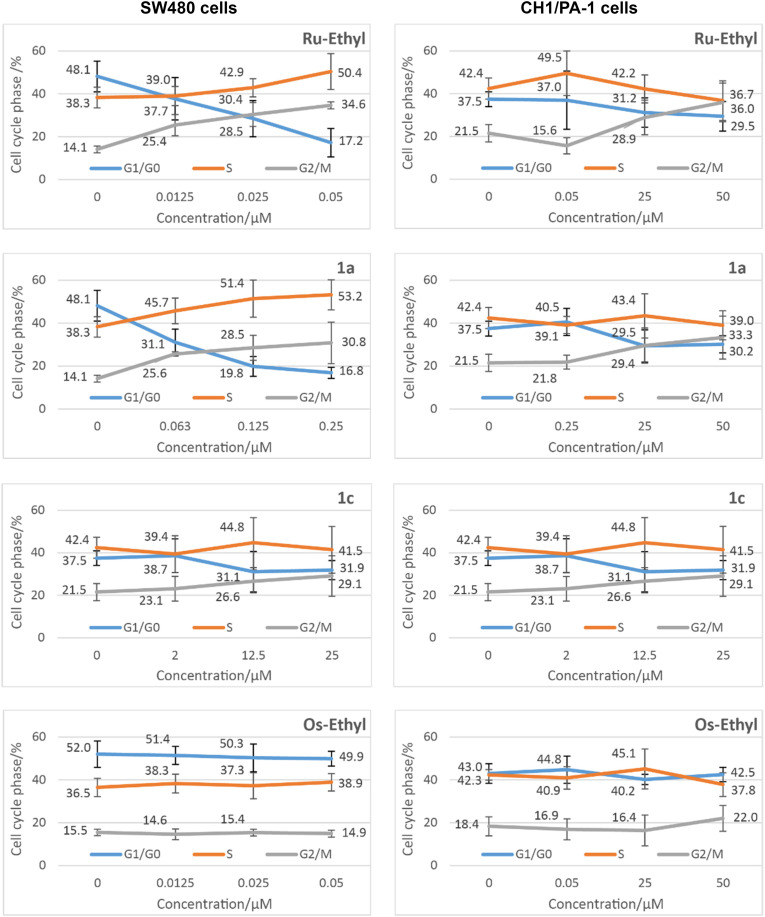
Cell cycle distribution in SW480 colon cancer cells (left) and CH1/PA-1 ovarian cancer (right) cells upon 24 h exposure to ruthenium and osmium complexes with different alkyl-substituents.

## Experimental part

### Materials and methods

**Fig. 8:**
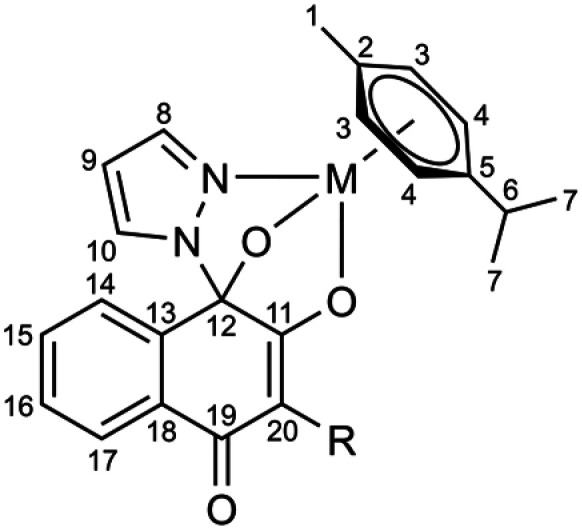
Numbering scheme for NMR assignments.

Ruthenium dimer [RuCl_2_(*p*-cym)]_2_,^44^ osmium dimer [OsCl_2_(*p*-cym)]_2_,^44^ Ru-Ethyl^32^ and Os-Ethyl^40^ were synthesized in accordance to published procedures. Following chemicals, materials and solvents were used without further purification: ruthenium(iii)chloride hydrate (Johnson Matthey), osmium tetroxide (Johnson Matthey), 1*H*-pyrazole (Acros), triethylamine (Fisher/Acros), 2-hydroxy-1,4-naphthoquinone (Acros-Fisher), acetaldehyde (Sigma-Aldrich), propionaldehyde (TCI Europe), butyraldehyde (TCI Europe), isobutyraldehyde (Sigma-Aldrich), 2-ethyl-butyraldehyde (Sigma-Aldrich), l-proline (Merck), Hantzsch ester (TCI), silver nitrate (abcr), pivalic acid (Fluka), 3,3-dimethylbutyric acid (Fisher/Acros), ammonium persulfate (Fisher/Acros), hydrochloric acid (37%) (Fluka), 4-(dimethylamino)-benzaldehyde (Sigma-Aldrich), acetic acid (Merck), l-Kyn (Sigma-Aldrich). Methanol (Sigma Aldrich), ethyl acetate (Riedel-de Haën), acetonitrile (Sigma-Aldrich), ethanol (96%, Brenntag), dichloromethane (DCM) (Sigma-Aldrich) and *n*-hexane (Sigma-Aldrich) were used without further purification. DCM was dried over anhydrous calcium chloride, filtrated and stored over molecular sieve (4 Å) under inert conditions. Microwave syntheses were conducted using a Biotage® Initiator+ instrument. Compounds were purified using a Biotage® Isolera™ system with silica packed columns (silica 60, 40–63 µM, Macherey-Nagel). Samples from stability measurements were analyzed on a Dionex Thermo Scientific UltiMate 3000 HPLC system, equipped with a HPG-3400RS binary pump and a DAD-3000 UV-VIS detector. High resolution ESI mass spectra of the metalacycles were recorded at the Mass Spectrometry Center of the University of Vienna (Faculty of Chemistry) on a Bruker maXis ESI-Qq-TOF mass spectrometer. Elemental analyses were performed by the Microanalytical Laboratory of the University of Vienna with an Eurovector EA3000 (2009) CHNSO analyzer equipped with a high temperature pyrolysis furnace (HT, Hekatech, Germany, 2009). O-analysis used the HT 1500 high temperature unit coupled to the above instrument. Carbon monoxide is used as analytical species to quantify oxygen. Elemental analyses samples were weighed on a Sartorius SEC 2 ultra-micro balance with ±0.1 µg resolution. Sample weights of 1–3 mg were used. For calibration two NIST-certified reference materials were used: sulfanilamide (C_6_H_8_N_2_O_2_S) and BBOT (2,5-bis-(5-*tert*-butyl-2-benzoxazol-2-yl)-thiophenone, C_26_H_26_N_2_O_2_S). The limit of quantification (LOQ) was 0.05 wt% for C, H, N and 0.02 wt% for S. The presented values are the average of determinations in triplicate. NMR spectra were measured on an AV III HD 700 Bruker BioSpin 700 MHz instrument or an AV III 600 Bruker BioSpin 600 MHz spectrometer. NMR signals were assigned according to the following numbering scheme (Fig. 8).

### Ligand synthesis

#### General procedure A

Lawsone (1 equiv.), Hantzsch ester (1.1 equiv.) and the respective aldehyde (2 equiv.) were taken up in dry DCM (12–20 mL) and stirred for five minutes at r.t. l-Proline (0.2 equiv.) was added and the resulting yellow suspension was stirred under microwave irradiation (85–100 °C, 25–60 minutes). The dark red solution was washed with 10% HCl and brine. The organic layer was dried over anhydrous sodium sulfate and the solvent was removed *in vacuo*, yielding the crude product as a solid or viscous oil. Pure product was obtained as a yellow solid *via* column chromatography (silica, gradient 40–80% DCM in *n*-hexane).

#### General procedure B

2-Hydroxy-1,4-naphthoquinone (1 equiv.), silver nitrate (0.2 equiv.) and the respective carboxylic acid (2 or 4 equiv.) were taken up in a mixture of ACN in H_2_O (1 : 1) to form an orange suspension. It was heated up to 65–78 °C (oil bath temperature) and a solution of ammonium peroxodisulfate (2 equiv.) in water was added slowly, to result in precipitation of a yellow solid. After two hours, it was cooled to r.t. and 10% HCl was added. The mixture was extracted with DCM, the organic phase was washed with brine and dried over anhydrous sodium sulfate. The solvent was removed under reduced pressure to give the crude product as a solid. The crude product was purified *via* column chromatography (silica, gradient 40–80% DCM in *n*-hexane) yielding the product as a yellow solid.

#### 2-Propyl-3-hydroxynaphthalene-1,4-dione: a

The product was synthesized according to general procedure A, using lawsone (506 mg, 2.91 mmol, 1 equiv.), Hantzsch ester (800 mg, 3.16 mmol, 1.1 equiv.), propionaldehyde (412 µL, 334 mg, 5.74 mmol, 2 equiv.) and l-proline (66 mg, 0.57 mmol, 0.2 equiv.) in dry DCM (20 mL). The mixture was stirred at 85 °C for 25 min under microwave irradiation. The crude product (*R*_f_ 0.25, 50% DCM in *n*-hexane) was purified *via* column chromatography (silica, gradient 40–80% DCM in *n*-hexane), to yield a as a yellow solid (536 mg, 2.48 mmol, 85%). ^1^H NMR (600 MHz, CDCl_3_) *δ* 8.12 (d, *J* = 7.7 Hz, 1H, 5/8), 8.08 (d, *J* = 7.6 Hz, 1H, 5/8), 7.75 (dd, *J* = 7.5, 7.5 Hz, 1H, 6/7), 7.68 (dd, *J* = 7.5, 7.5 Hz, 1H, 6/7), 7.29 (s, 1H, 1), 2.59 (t, *J* = 7.6 Hz, 2H, 12), 1.61–1.56 (m, *J* = 14.9, 7.5 Hz, 2H, 13), 0.99 (t, *J* = 7.3 Hz, 3H, 14) ppm. ^13^C NMR (151 MHz, CDCl_3_) *δ* 184.7 (10), 181.5 (3), 153.08 (2), 134.8 (6/7), 133.0 (4/9), 132.8 (6/7), 129.5 (4/9), 126.8 (5/8), 126.0 (5/8), 124.6 (11), 25.3 (12), 21.6 (13), 14.2 (14) ppm. Elemental analysis calculated for C_13_H_12_O_3_: C 72.21%, H 5.59%, O 22.20%. Found: C 71.86%, H 5.55%, O 22.03%.

#### 2-Hydroxy-3-propylnaphthalene-1,4-dione: b

The product was synthesized according to general procedure A, using lawsone (504 mg, 2.89 mmol, 1 equiv.), Hantzsch ester (810 mg, 3.20 mmol, 1.1 equiv.), butyraldehyde (517 µL, 414 mg, 5.74 mmol, 2 equiv.) and l-proline (70 mg, 0.61 mmol, 0.2 equiv.) in dry DCM (20 mL). The mixture was stirred at 85 °C for 25 min under microwave irradiation. The crude product was purified *via* column chromatography (silica, gradient 40–80% DCM in *n*-hexane), to yield b as a yellow solid (531 mg, 2.31 mmol, 80%). ^1^H NMR (700 MHz, CDCl_3_) *δ* 8.12 (dd, *J* = 7.7, 0.5 Hz, 1H, 5/8), 8.07 (dd, *J* = 7.6, 0.7 Hz, 1H, 5/8), 7.75 (ddd, *J* = 7.6, 1.1 Hz, 1H, 6/7), 7.68 (ddd, *J* = 7.5, 1.1 Hz, 1H, 6/7), 7.28 (s, 1H, 1), 2.62–2.59 (m, 2H, 12), 1.55–1.50 (m, 2H, 13), 1.42–1.38 (m, 2H, 14), 0.94 (t, *J* = 7.4 Hz, 3H, 15) ppm. ^13^C NMR (176 MHz, CDCl_3_) *δ* 184.7 (10), 181.5 (3), 153.0 (2), 134.8 (7), 133.0 (4/9), 132.8 (7), 129.4 (4/9), 126.8 (5/8), 126.0 (5/8), 124.8 (11), 30.4 (12), 23.1 (13), 22.9 (14), 13.9 (15) ppm. Elemental analysis calculated for C_14_H_14_O_3_: C 73.03%, H 6.13%, O 20.85%. Found: C 72.70%, H 6.17%, O 20.65%.

#### 2-Hydroxy-3-isobutylnaphthalene-1,4-dione: c

The product was synthesized according to general procedure **A**, using lawsone (503 mg, 2.89 mmol, 1 equiv.), Hantzsch ester (817 mg, 3.22 mmol, 1.1 equiv.), isobutyraldehyde (424 µL, 414 mg, 5.74 mmol, 2 equiv.) and l-proline (72 mg, 0.62 mmol, 0.2 equiv.) in dry DCM (20 mL). The mixture was stirred at 90 °C for 45 min under microwave irradiation. The crude product was purified *via* column chromatography (silica, gradient 40–80% DCM in *n*-hexane), to yield c as a yellow solid (392 mg, 1.70 mmol, 59%). ^1^H NMR (600 MHz, CDCl_3_) *δ* 8.12 (dd, *J* = 7.7 Hz, 1H, 5/8), 8.08 (dd, *J* = 7.6 Hz, 1H, 5/8), 7.75 (ddd, *J* = 7.5 Hz, 1H, 6/7), 7.68 (ddd, *J* = 7.5 Hz, 1H, 6/7), 7.29 (s, 1H, 1), 2.51 (d, *J* = 7.3 Hz, 2H, 12), 1.99 (sept, *J* = 13.5, 6.7 Hz, 1H, 13), 0.95 (d, *J* = 6.6 Hz, 6H, 14) ppm. ^13^C NMR (151 MHz, CDCl_3_) *δ* 185.0 (10), 181.6 (3), 153.6 (2), 135.0 (6/7), 133.1 (4/9), 133.0 (6/7), 129.6 (4/9), 127.0 (5/8), 126.2 (5/8), 124.1 (11), 32.4 (12), 28.3 (13), 22.9 (14) ppm. Elemental analysis calculated for C_14_H_14_O_3_: C 73.03%, H 6.13%, O 20.85%. Found: C 72.93%, H 6.14%, O 20.71%.

#### 2-(*tert*-Butyl)-3-hydroxynaphthalene-1,4-dione: d

The product was synthesized in accordance to general procedure **B**, using lawsone (1.5 g, 8.62 mmol, 1 equiv.), silver nitrate (0.293 g, 1.72 mmol, 0.2 equiv.) and 3,3-dimethylbutanoic acid (1.330 g, 13.02 mmol, 1.5 equiv.). The mixture was heated up to 65 °C (oil bath temperature) and a solution of ammonium peroxodisulfate (3 g, 13.14 mmol, 1.5 equiv.) in water (10 mL) was added. After work up, the crude product was purified *via* column chromatography (silica, gradient 40–80% DCM in *n*-hexane) yielding d as a yellow solid (0.531 g, 2.31 mmol, 27%). ^1^H NMR (700 MHz, CDCl_3_) *δ* 8.06 (dd, *J* = 7.7, 0.5 Hz, 1H, 5/8), 8.03 (dd, *J* = 7.6, 0.7 Hz, 1H, 5/8), 7.84 (s, 1H, 1), 7.74 (ddd, *J* = 7.6, 1.2 Hz, 1H, 6/7), 7.64 (ddd, *J* = 7.5, 1.1 Hz, 1H, 6/7), 1.48 (s, 9H, 13) ppm. ^13^C NMR (176 MHz, CDCl_3_) *δ* 185.6 (10), 182.5 (3), 152.6 (2), 135.3 (6/7), 134.7 (4/9), 132.6 (6/7), 129.9 (11), 128.5 (4/9), 127.1 (5/8), 125.9 (5/8), 36.2 (12), 30.7 (13) ppm. Elemental analysis calculated for C_14_H_14_O_3_: C 73.03%, H 6.13%, O 20.85%. Found: C 72.78%, H 6.12%, O 21.01%.

#### 2-Hydroxy-3-neopentylnaphthalene-1,4-dione: e

The product was synthesized in accordance to general procedure **B**, using lawsone (1.547 g, 8.89 mmol, 1 equiv.), silver nitrate (0.296 g, 1.74 mmol, 0.2 equiv.) and 3,3-dimethylbutanoic acid (4.39 mL, 4.003 g, 34.46 mmol, 4 equiv.). The mixture was heated up to 65 °C (oil bath temperature) and a solution of ammonium peroxodisulfate (3.939 g, 17.26 mmol, 2 equiv.) in water (10 mL) was added. After work up, the crude product was purified *via* column chromatography (silica, gradient 40–80% DCM in *n*-hexane) yielding e as a yellow solid (0.550 g, 2.25 mmol, 25%). ^1^H NMR (600 MHz, CDCl_3_) *δ* 8.12 (d, *J* = 9.8 Hz, 1H, 5/8), 8.09 (d, *J* = 7.6 Hz, 1H, 5/8), 7.75 (dd, *J* = 7.6, 1.0 Hz, 1H, 6/7), 7.68 (dd, *J* = 7.5, 1.0 Hz, 1H, 6/7), 7.40 (s, 1H, 1), 2.59 (s, 2H, 12), 0.96 (s, 9H, 14) ppm. ^13^C NMR (151 MHz, CDCl_3_) *δ* 185.0 (10), 181.5 (3), 154.0 (2), 134.9 (6/7), 133.1 (4/9), 132.8 (6/7), 129.4 (4/9), 127.0 (5/8), 126.0 (5/8), 123.0 (11), 36.0 (12), 33.9 (13), 30.3 (14) ppm. Elemental analysis calculated for C_15_H_16_O_3_: C 73.75%, H 6.60%, O 19.65%. Found: C 73.55%, H 6.77%, O 19.24%.

#### 2-(2-Ethylbutyl)-3-hydroxynaphthalene-1,4-dione: f

The product was synthesized according to general procedure **A**, using lawsone (505 mg, 2.90 mmol, 1 equiv.), Hantzsch ester (813 mg, 3.21 mmol, 1.1 equiv.), 2-ethylbutanal (706 µL, 575 mg, 5.74 mmol, 2 equiv.) and l-proline (72 mg, 0.63 mmol, 0.2 equiv.) in dry DCM (20 mL). The mixture was stirred at 100 °C for 60 min under microwave irradiation. The crude product (*R*_f_ 0.59, 50% DCM in *n*-hexane) was purified *via* column chromatography (silica, gradient 40–80% DCM in *n*-hexane), to yield f as a yellow solid (273 mg, 1.05 mmol, 36%). ^1^H NMR (700 MHz, CDCl_3_) *δ* 8.12 (dd, *J* = 7.7 Hz, 1H, 5/8), 8.08 (dd, *J* = 7.6 Hz, 1H, 5/8), 7.75 (ddd, *J* = 7.5 Hz, 1H, 6/7), 7.68 (ddd, *J* = 7.5 Hz, 1H, 6/7), 7.30 (s, 1H, 1), 2.55 (d, *J* = 7.3 Hz, 2H, 12), 1.66–1.62 (m, 1H, 13), 1.37–1.31 (m, 4H, 14), 0.90 (t, *J* = 7.4 Hz, 6H, 15) ppm. ^13^C NMR (176 MHz, CDCl_3_) *δ* 185.1 (10), 181.5 (3), 153.7 (2), 135.0 (6/7), 133.2 (4/9), 133.0 (6/7), 129.6 (4/9), 127.0 (5/8), 126.2 (5/8), 124.6 (11), 40.4 (12), 27.8 (13), 25.7 (14), 10.9 (15) ppm. Elemental analysis calculated for C_16_H_18_O_3_: C 74.40%, H 7.02%, O 18.58%. Found: C 74.02%, H 7.04%, O 18.62%.

### Complex syntheses

#### General procedure

Ruthenium or osmium dimer (1 equiv.), pyrazole (1.9 equiv.) and the respective naphthoquinone derivative (1.9 equiv.) were dissolved in methanol (12 mL) and triethylamine (6 equiv.) was added. Stirring under microwave irradiation (60 °C, 20 min) was followed by solvent removal under reduced pressure. The crude product was purified *via* column chromatography (silica, isocratic, 70% ethyl acetate, 5% triethylamine in *n*-hexane). The fractions where combined and the solvent was removed *in vacuo*. The residue was dissolved in a small amount of DCM, the product precipitated by the addition of *n*-hexane and stored at 4 °C overnight. The formed pale yellow to greenish solid was separated by filtration and dried at 60 °C.

#### [(3-Propyl-1-(1*H*-κ*N*2-pyrazol-1-yl)-4-oxo-1,4-dihydronaphtalene-1,2-bis(olato)-κ*O*1-κ*O*2)(η^6^-*p*-cymene)ruthenium(ii)]: 1a

The product was synthesized according to the general procedure, using ruthenium(ii) dimer (160 mg, 0.26 mmol, 1 equiv.), naphthoquinone derivative a (107 mg, 0.47 mmol, 1.9 equiv.), pyrazole (34 mg, 0.50 mmol, 1.9 equiv.) and triethylamine (217 µL, 159 mg, 1.57 mmol, 6 equiv.) in methanol (12 mL). Stirring under microwave irradiation led to the formation of a red solution. The solvent was removed under reduced pressure and the crude product was purified *via* column chromatography (*R*_f_ 0.24, silica, isocratic 70% ethyl acetate and 5% triethylamine in *n*-hexane). Precipitation from DCM/*n*-hexane led to the desired product 1a as a yellow powder (163 mg, 0.31 mmol, 60%). ^1^H NMR (700 MHz, MeOD) *δ* 8.32 (d, *J* = 1.5 Hz, 1H, 8), 8.09 (dd, *J* = 5.5, 3.1 Hz, 1H, 14), 7.64–7.55 (m, 3H, 15–17), 6.68 (d, *J* = 2.2 Hz, 1H, 10), 6.34 (s, 1H, 9), 5.96 (d, *J* = 5.8 Hz, 1H, 4), 5.87 (d, *J* = 5.8 Hz, 1H, 4), 5.60 (dd, *J* = 6.7, 6.7 Hz, 2H, 3), 2.86 (hept, *J* = 6.8 Hz, 1H, 6), 2.33 (s, 3H, 1), 2.38–2.22 (m, 2H, 21), 1.41–1.23 (m, 2H, 22), 1.33 (d, *J* = 6.9 Hz, 6H, 7), 0.76 (t, *J* = 7.4 Hz, 3H, 23) ppm. ^13^C NMR (176 MHz, MeOD) *δ* 184.1, 183.3 (11 and 19), 141.4 (8), 137.7, 134.4 (13 and 18), 132.2, 131.2 (15–17), 127.9, 127.7 (15–17 and 14), 127.1 (10), 110.5 (20), 108.7 (9), 101.2 (5), 98.5 (2), 94.9 (12), 83.4 (4), 82.8 (4), 80.2 (3), 32.7 (6), 25.6 (21), 23.1 (7), 22.9 (22), 22.7 (7), 18.3 (1), 14.3 (23) ppm. ESI-HR-MS [M + H^+^] *m*/*z* found: 519.1223 (519.12162), [M + Na^+^] *m*/*z* found: 541.1044 (541.10356). Elemental analysis calculated for C_26_H_28_N_2_O_3_Ru: C 60.33%, H 5.45%, N 5.41%, O 9.27%. Found: C 59.95%, H 5.45%, N 5.49%, O 9.16%.

#### [(3-Butyl-1-(1*H*-κ*N*2-pyrazol-1-yl)-4-oxo-1,4-dihydronaphtalene-1,2-bis(olato)-κ*O*1-κ*O*2)(η^6^-*p*-cymene)ruthenium(ii)]: 1b

The product was synthesized according to the general procedure, using ruthenium(ii) dimer (160 mg, 0.26 mmol, 1 equiv.), naphthoquinone derivative b (107 mg, 0.47 mmol, 1.9 equiv.), pyrazole (33 mg, 0.48 mmol, 1.9 equiv.) and triethylamine (204 µL, 149 mg, 1.47 mmol, 6 equiv.) in methanol (12 mL). Stirring under microwave irradiation led to the formation of a brown/blackish solution. The solvent was removed under reduced pressure and the crude product was purified *via* column chromatography (*R*_f_ 0.30, silica, isocratic 70% ethyl acetate and 5% triethylamine in *n*-hexane). Precipitation from DCM/*n*-hexane led to the desired product 1b as a green powder (161 mg, 0.30 mmol, 58%). ^1^H NMR (600 MHz, MeOD) *δ* 8.33 (d, *J* = 2.0 Hz, 1H, 8), 8.11–8.06 (m, 1H, 14), 7.62–7.57 (m, 3H, 15–17), 6.68 (d, *J* = 2.4 Hz, 1H, 10), 6.34 (dd, *J* = 2.3, 2.3 Hz, 1H, 9), 5.96 (d, *J* = 5.9 Hz, 1H, 4), 5.87 (d, *J* = 5.9 Hz, 1H, 4), 5.60 (dd, *J* = 6.1, 6.1 Hz, 2H, 3), 2.87 (hept, *J* = 6.9 Hz, 1H, 6), 2.39–2.34 (m, 1H, 21), 2.33 (s, 3H, 1), 2.31–2.26 (m, 1H, 21), 1.34 (d, *J* = 6.9 Hz, 6H, 7), 1.32–1.28 (m, 1H, 22), 1.28–1.22 (m, 1H, 22), 1.21–1.15 (m, 2H, 23), 0.83 (t, *J* = 7.3 Hz, 3H, 24) ppm. ^13^C NMR (151 MHz, MeOD) *δ* 184.0, 183.3 (11 and 19), 141.4 (8), 137.7, 134.4 (13 and 18), 132.2 (15–17), 131.2 (15–17), 127.9 (14), 127.7, 127.1 (15–17 and 10), 110.8 (20), 108.7 (9), 101.2 (5), 98.5 (2), 94.9 (12), 83.4 (4), 82.8 (4), 80.2 (3), 32.6 (6), 32.1 (21), 23.6 (22), 23.3 (23), 23.1 (7), 22.8 (7), 18.4 (1), 14.5 (24) ppm. ESI-HR-MS [M + H^+^] *m*/*z* found: 533.1370 (533.13727), [M + Na^+^] *m*/*z* found: 555.1188 (555.11921). Elemental analysis calculated for C_27_H_30_N_2_O_3_Ru·0.15 H_2_O: C 60.69%, H 5.72%, N 5.24%, O 9.43%. Found: C 60.50%, H 5.67%, N 5.35%, O 9.15%.

#### [(3-Isobutyl-1-(1*H*-κ*N*2-pyrazol-1-yl)-4-oxo-1,4-dihydronaphtalene-1,2-bis(olato)-κ*O*1-κ*O*2)(η^6^-*p*-cymene)ruthenium(ii)]: 1c

The product was synthesized according to the general procedure, using ruthenium(ii) dimer (105 mg, 0.17 mmol, 1 equiv.), naphthoquinone derivative c (72 mg, 0.31 mmol, 1.9 equiv.), pyrazole (21 mg, 0.31 mmol, 1.9 equiv.) and triethylamine (136 µL, 99 mg, 0.98 mmol, 6 equiv.) in methanol (12 mL). Stirring under microwave irradiation led to the formation of a brown/blackish solution. The solvent was removed under reduced pressure and the crude product was purified *via* column chromatography (*R*_f_ 0.30, silica, isocratic 70% ethyl acetate and 5% triethylamine in *n*-hexane). Precipitation from DCM/*n*-hexane led to the desired product 1c as a yellow powder (116.0 mg, 0.22 mmol, 64%). ^1^H NMR (700 MHz, MeOD) *δ* 8.33 (d, *J* = 1.4 Hz, 1H, 8), 8.10 (dd, *J* = 5.5, 3.2 Hz, 1H, 14), 7.65–7.59 (m, 3H, 15–17), 6.70 (d, *J* = 2.0 Hz, 1H, 10), 6.35 (s, 1H, 9), 5.98 (d, *J* = 5.8 Hz, 1H, 4), 5.90 (d, *J* = 5.8 Hz, 1H, 4), 5.62 (d, *J* = 5.8 Hz, 1H, 3), 5.58 (d, *J* = 5.8 Hz, 1H, 3), 2.90–2.83 (m, 1H, 6), 2.34 (s, 3H, 1), 2.25–2.17 (m, 2H, 21), 1.74 (hept, *J* = 6.6 Hz, 1H, 22), 1.35 (d, *J* = 6.3 Hz, 6H, 7), 0.80 (d, *J* = 6.6 Hz, 3H, 23), 0.64 (d, *J* = 6.6 Hz, 3H, 23) ppm. ^13^C NMR (176 MHz, MeOD) *δ* 184.5 (11 or 19), 183.5 (11 or 19), 141.4 (8), 137.7 (13 or 18), 134.4 (13 or 18), 132.1 (15–17), 131.2 (15–17), 127.8, 127.6, 127.1 (10, 15–17 or 14), 109.8 (20), 108.7 (9), 101.0 (5), 98.6 (2), 95.0 (12), 83.6 (4), 82.9 (4), 80.1 (3), 80.0 (3), 32.6 (6), 32.6 (21), 29.0 (22), 23.2, 22.7 (7 and 23), 18.4 (1) ppm. ESI-HR-MS [M + H^+^] *m*/*z* found: 533.1371 (533.13727), [M + Na^+^] *m*/*z* found: 555.1187 (555.11921). Elemental analysis calculated for C_27_H_30_N_2_O_3_Ru·0.15 H_2_O: C 60.69%, H 5.72%, N 5.24%, O 9.43%. Found: C 60.43%, H 5.66%, N 5.44%, O 9.24%.

#### [(3-(*tert*-Butyl)-1-(1*H*-κ*N*2-pyrazol-1-yl)-4-oxo-1,4-dihydronaphtalene-1,2-bis(olato)-κ*O*1-κ*O*2)(η^6^-*p*-cymene)ruthenium(ii)]: 1d

The product was synthesized according to the general procedure, using ruthenium(ii) dimer (151 mg, 0.25 mmol, 1 equiv.), naphthoquinone derivative e (108 mg, 0.47 mmol, 1.9 equiv.), pyrazole (33 mg, 0.48 mmol, 1.9 equiv.) and triethylamine (203 µL, 148 mg, 1.46 mmol, 6 equiv.) in methanol (12 mL). Stirring under microwave irradiation led to formation of a reddish solution. The solvent was removed under reduced pressure and the crude product was purified *via* column chromatography (silica, isocratic 70% ethyl acetate and 5% triethylamine in *n*-hexane). Precipitation with DCM/*n*-hexane led to the product 1e as a yellow powder (181 mg, 0.33 mmol, 71%). ^1^H NMR (600 MHz, MeOD) *δ* 8.29 (d, *J* = 2.1 Hz, 1H, 8), 8.04–7.95 (m, 1H, 14), 7.59–7.49 (m, 3H, 15–17), 6.65 (d, *J* = 2.5 Hz, 1H, 10), 6.32 (dd, *J* = 2.4, 2.4 Hz, 1H, 9), 5.95 (d, *J* = 5.9 Hz, 1H, 4), 5.88 (d, *J* = 5.8 Hz, 1H, 4), 5.61 (d, *J* = 5.8 Hz, 1H, 3), 5.54 (d, *J* = 5.8 Hz, 1H, 3), 2.85 (hept, *J* = 6.9 Hz, 1H, 6), 2.34 (s, 3H, 1), 1.32 (d, *J* = 7.0, 1.7 Hz, 6H, 7), 1.28 (s, 9H, 22) ppm. ^13^C NMR (151 MHz, MeOD) *δ* 184.0 (11 or 19), 183.4 (11 or 19), 141.2 (8), 136.9 (13 or 18), 135.9 (13 or 18), 131.7 (15–17), 131.1 (15–17), 127.6 (15–17, 14 or 10), 127.1 (two of 15–17, 14 or 10), 116.3 (20), 108.5 (9), 100.7 (5), 99.1 (2), 95.8 (12), 83.8 (4), 83.3 (4), 79.9 (3), 79.8 (3), 35.6 (21), 32.7 (6), 31.4 (22), 23.2 (7), 22.9 (7), 18.9 (1) ppm. ESI-HR-MS [M + H^+^] *m*/*z* found: 533.1379 (533.13727), [M + Na^+^] *m*/*z* found: 555.1199 (555.11921). Elemental analysis calculated for C_27_H_30_N_2_O_3_Ru·0.1 H_2_O: C 60.80%, H 5.71%, N 5.25%, O 9.30%. Found: C 60.50%, H 5.68%, N 5.31%, O 9.08%.

#### [(3-Neopentyl-1-(1*H*-κ*N*2-pyrazol-1-yl)-4-oxo-1,4-dihydronaphtalene-1,2-bis(olato)-κ*O*1-κ*O*2)(η^6^-*p*-cymene)ruthenium(ii)]: 1e

The product was synthesized according to the general procedure, using ruthenium(ii) dimer (110 mg, 0.18 mmol, 1 equiv.), naphthoquinone derivative e (84 mg, 0.34 mmol, 1.9 equiv.), pyrazole (23 mg, 0.34 mmol, 1.9 equiv.) and triethylamine (149 µL, 109 mg, 1.08 mmol, 6 equiv.) in methanol (12 mL). Stirring under microwave irradiation led to formation of a brown/blackish solution. The solvent was removed under reduced pressure and the crude product was purified *via* column chromatography (*R*_f_ 0.35, silica, isocratic 70% ethyl acetate and 5% triethylamine in *n*-hexane). Precipitation with DCM/*n*-hexane led to the product 1e as a yellow powder (153 mg, 0.28 mmol, 78%). ^1^H NMR (600 MHz, MeOD) *δ* 8.31 (d, *J* = 2.1 Hz, 1H, 8), 8.11–8.04 (m, 1H, 14), 7.66–7.55 (m, 3H, 15–17), 6.71 (d, *J* = 2.5 Hz, 1H, 10), 6.33 (dd, *J* = 2.4, 2.4 Hz, 1H, 9), 5.95 (d, *J* = 6.0, 1.1 Hz, 1H, 4), 5.90 (d, *J* = 6.0, 1.1 Hz, 1H, 4), 5.62 (d, *J* = 6.0, 1.1 Hz, 1H, 3), 5.52 (d, *J* = 5.8, 1.1 Hz, 1H, 3), 2.86 (hept, *J* = 6.9 Hz, 1H, 6), 2.33 (s, 3H, 1), 2.35–2.24 (m, 2H, 21), 1.40–1.35 (m, 6H, 7), 0.68 (s, 9H, 23) ppm. ^13^C NMR (151 MHz, MeOD) *δ* 185.1 (11 or 19), 183.6 (11 or 19), 141.4 (8), 137.6 (13 or 18), 134.5 (13 or 18), 132.1 (15–17), 131.2 (15–17), 127.7, 127.6 and 127.3 (15–17, 14 or 10), 108.7 (9), 108.6 (20), 100.8 (5), 98.7 (2), 95.1 (12), 83.8 (4), 83.1 (4), 80.1 (3), 79.9 (3), 36.1 (21), 34.3 (22), 32.5 (6), 30.5 (23), 23.2 (7), 22.9 (7), 18.5 (1) ppm. ESI-HR-MS [M + H^+^] *m*/*z* found: 547.1536 (547.15292), [M + Na^+^] *m*/*z* found: 569.1354 (569.13486). Elemental analysis calculated for C_28_H_32_N_2_O_3_Ru·0.15 H_2_O: C 61.33%, H 5.94%, N 5.11%, O 9.19%. Found: C 61.10%, H 5.93%, N 5.25%, O 8.88%.

#### [(3-(2-Ethylbutyl)-1-(1*H*-κ*N*2-pyrazol-1-yl)-4-oxo-1,4-dihydronaphtalene-1,2-bis(olato)-κ*O*1-κ*O*2)(η^6^-*p*-cymene)ruthenium(ii)]: 1f

The product was synthesized according to the general procedure, using ruthenium(ii) dimer (113 mg, 0.18 mmol, 1 equiv.), naphthoquinone derivative f (88 mg, 0.34 mmol, 1.9 equiv.), pyrazole (24 mg, 0.35 mmol, 1.9 equiv.) and triethylamine (149 µL, 109 mg, 1.08 mmol, 6 equiv.) in methanol (12 mL). Stirring under microwave irradiation led to the formation of a black solution. The solvent was removed under reduced pressure and the crude product was purified *via* column chromatography (*R*_f_ 0.32, silica, isocratic 70% ethyl acetate and 5% triethylamine in *n*-hexane). Precipitation from DCM/*n*-hexane led to the desire product 1f as a yellow powder (107 mg, 0.19 mmol, 52%). ^1^H NMR (600 MHz, MeOD) *δ* 8.32 (d, *J* = 2.1 Hz, 1H, 1), 8.11–8.03 (m, 1H, 14), 7.67–7.53 (m, 3H, 15–17), 6.68 (d, *J* = 2.5 Hz, 1H, 10), 6.33 (dd, *J* = 2.4, 2.4 Hz, 1H, 9), 5.96 (d, *J* = 5.9 Hz, 1H, 4), 5.89 (d, *J* = 5.9 Hz, 1H, 4), 5.61 (d, *J* = 5.9 Hz, 1H, 3), 5.56 (d, *J* = 5.9 Hz, 1H, 3), 2.95–2.86 (m, 1H, 6), 2.33 (s, 3H, 1), 2.32–2.21 (m, 2H, 21), 1.44–1.37 (m, 1H, 22), 1.34 (d, *J* = 6.9, 2.0 Hz, 6H, 7), 1.22–1.14 (m, 2H, 23), 1.09–0.98 (m, 2H, 23), 0.80 (t, *J* = 7.4 Hz, 3H, 24), 0.74 (t, *J* = 7.4 Hz, 3H, 24) ppm. ^13^C NMR (151 MHz, MeOD) *δ* 184.4 (11 or 19), 183.5 (11 or 19), 141.4 (8), 137.7 (13 or 18), 134.4 (13 or 18), 132.1 (15–17), 131.2 (15–17), 127.8 (15–17, 14 or 10), 127.6 (15–17, 14 or 10), 127.1 (15–17, 14 or 10), 110.0 (20), 108.7 (9), 100.9 (5), 98.7 (2), 95.0 (12), 83.7 (4), 83.0 (4), 80.1 (3), 80.0 (3), 41.2 (22), 32.6 (6), 27.8 (21), 26.7 (23), 26.3 (23), 23.2 (7), 22.8 (7), 18.4 (1), 11.6 (24), 11.3 (24) ppm. ESI-HR-MS [M + H^+^] *m*/*z* found: 561.1688 (561.16857), [M + Na^+^] *m*/*z* found: 583.1508 (583.15051). Elemental analysis calculated for C_29_H_34_N_2_O_3_Ru·0.15 H_2_O: C 61.94%, H 6.15%, N 4.98%, O 8.96%. Found: C 61.63%, H 6.21%, N 5.01%, O 8.71%.

#### [(3-Propyl-1-(1*H*-κ*N*2-pyrazol-1-yl)-4-oxo-1,4-dihydronaphtalene-1,2-bis(olato)-κ*O*1-κ*O*2)(η^6^-*p*-cymene)osmium(ii)]: 2a

The product was synthesized according to the general procedure, using osmium(ii) dimer (151 mg, 0.25 mmol, 1 equiv.), naphthoquinone derivative a (108 mg, 0.47 mmol, 1.9 equiv.), pyrazole (33 mg, 0.48 mmol, 1.9 equiv.) and triethylamine (203 µL, 148 mg, 1.46 mmol, 6 equiv.) in methanol (12 mL). Stirring under microwave irradiation led to formation of a reddish solution. The solvent was removed under reduced pressure and the crude product was purified *via* column chromatography (silica, isocratic 70% ethyl acetate and 5% triethylamine in *n*-hexane). Precipitation with DCM/*n*-hexane led to the product 2a as a yellow powder (181 mg, 0.33 mmol, 71%). ^1^H NMR (600 MHz, MeOD) *δ* 8.28 (d, *J* = 2.2 Hz, 1H, 8), 8.16–8.10 (m, 1H, 14), 7.66–7.60 (m, 3H, 15–17), 6.87 (d, *J* = 2.6 Hz, 1H, 10), 6.39 (dd, *J* = 2.4, 2.4 Hz, 1H, 9), 6.17 (d, *J* = 5.4 Hz, 1H, 4), 6.09 (d, *J* = 5.4 Hz, 1H, 4), 5.84 (d, *J* = 5.4 Hz, 1H, 3), 5.79 (d, *J* = 5.4 Hz, 1H, 3), 2.74 (hept, *J* = 6.9 Hz, 1H, 6), 2.39 (s, 3H, 1), 2.38–2.26 (m, 2H, 21), 1.42–1.25 (m, 2H, 22), 1.31 (dd, *J* = 6.9 Hz, 6H, 7), 0.75 (t, *J* = 7.4 Hz, 3H, 23) ppm. ^13^C NMR (151 MHz, MeOD) *δ* 184.0 (11 or 19), 183.5 (11 or 19), 141.4 (8), 136.7 (13 or 18), 134.5 (13 or 18), 132.4 (15–17), 131.5 (15–17), 127.7 (15–17, 14 and 10), 127.5 (15–17, 14 and 10), 127.3 (15–17, 14 and 10), 110.3 (20), 109.4 (9), 98.2 (12), 91.0 (5), 88.9 (2), 73.9 (4), 73.2 (4), 70.3 (3), 33.1 (6), 25.6 (21), 23.5 (7), 23.1 (7), 22.8 (22), 18.6 (1), 14.2 (23) ppm. ESI-HR-MS [M + H^+^] *m*/*z* found: 609.1780 (609.17875), [M + Na^+^] *m*/*z* found: 631.1601 (631.16069). Elemental analysis calculated for C_26_H_28_N_2_O_3_Os·0.3 H_2_O: C 51.01%, H 4.71%, N 4.58%, O 8.63%. Found: C 50.63%, H 4.56%, N 4.69%, O 8.32%.

#### [(3-Butyl-1-(1*H*-κ*N*2-pyrazol-1-yl)-4-oxo-1,4-dihydronaphtalene-1,2-bis(olato)-κ*O*1-κ*O*2)(η^6^-*p*-cymene)osmium(ii)]: 2b

The product was synthesized according to the general procedure, using osmium(ii) dimer (113 mg, 0.14 mmol, 1 equiv.), naphthoquinone derivative b (62 mg, 0.27 mmol, 1.9 equiv.), pyrazole (18 mg, 0.26 mmol, 1.9 equiv.) and triethylamine (116 µL, 84 mg, 0.83 mmol, 6 equiv.) in methanol (12 mL). Stirring under microwave irradiation led to formation of a reddish solution. The solvent was removed under reduced pressure and the crude product was purified *via* column chromatography (silica, isocratic 70% ethyl acetate and 5% triethylamine in *n*-hexane). Precipitation with DCM/*n*-hexane led to the product 2b as a yellow powder (142 mg, 0.23 mmol, 88%). ^1^H NMR (700 MHz, MeOD) *δ* 8.28 (d, *J* = 2.1 Hz, 1H, 8), 8.16–8.10 (m, 1H, 14), 7.67–7.59 (m, 3H, 15–17), 6.87 (d, *J* = 2.5 Hz, 1H, 10), 6.39 (d, *J* = 2.3, 2.3 Hz, 1H, 9), 6.17 (d, *J* = 5.4 Hz, 1H, 4), 6.09 (d, *J* = 5.4 Hz, 1H, 4), 5.84 (d, *J* = 5.5 Hz, 1H, 3), 5.80 (d, *J* = 5.4 Hz, 1H, 3), 2.74 (hept, *J* = 6.9 Hz, 1H, 6), 2.39 (s, 3H, 1), 2.38–2.28 (m, 2H, 21), 1.31 (d, *J* = 6.9 Hz, 6H, 7), 1.29–1.22 (m, 2H, 22), 1.21–1.13 (m, 2H, 23), 0.82 (t, *J* = 7.3 Hz, 3H, 24) ppm. ^13^C NMR (176 MHz, MeOD) *δ* 184.0 (11 or 19), 183.4 (11 or 19), 141.4 (8), 136.7 (13 or 18), 134.5 (13 or 18), 132.4 (15–17), 131.5 (15–17), 127.7 (15–17, 14 or 10), 127.5 (15–17, 14 or 10), 127.3 (15–17, 14 or 10), 110.5 (20), 109.4 (9), 98.2 (12), 91.0 (5), 88.9 (2), 73.9 (4), 73.2 (4), 70.4 (3), 70.3 (3), 33.1 (6), 32.0 (21), 23.6 (22), 23.5 (7), 23.3 (23), 23.1 (7), 18.6 (1), 14.5 (24) ppm. ESI-HR-MS [M + H^+^] *m*/*z* found: 623.1930 (623.19440), [M + Na^+^] *m*/*z* found: 645.1750 (645.17634). Elemental analysis calculated for C_27_H_30_N_2_O_3_Os: C 52.24%, H 4.87%, N 4.51%, O 7.73%. Found: C 52.00%, H 4.87%, N 4.62%, O 7.81%.

#### [(3-Isobutyl-1-(1*H*-κ*N*2-pyrazol-1-yl)-4-oxo-1,4-dihydronaphtalene-1,2-bis(olato)-κ*O*1-κ*O*2)(η^6^-*p*-cymene)osmium(ii)]: 2c

The product was synthesized according to the general procedure, using osmium(ii) dimer (100 mg, 0.13 mmol, 1 equiv.), naphthoquinone derivative c (55 mg, 0.24 mmol, 1.9 equiv.), pyrazole (18 mg, 0.26 mmol, 1.9 equiv.) and triethylamine (105 µL, 77 mg, 0.76 mmol, 6 equiv.) in methanol (12 mL). Stirring under microwave irradiation led to formation of a reddish solution. The solvent was removed under reduced pressure and the crude product was purified *via* column chromatography (silica, isocratic 70% ethyl acetate and 5% triethylamine in *n*-hexane). Precipitation with DCM/*n*-hexane led to the product 2c as a yellow powder (121 mg, 0.19 mmol, 79%). ^1^H NMR (600 MHz, MeOD) *δ* 8.27 (d, *J* = 2.1 Hz, 1H, 8), 8.15–8.10 (m, 1H, 14), 7.68–7.59 (m, 3H, 15–17), 6.88 (d, *J* = 2.6 Hz, 1H, 10), 6.39 (dd, *J* = 2.4, 2.4 Hz, 1H, 9), 6.17 (d, *J* = 5.4 Hz, 1H, 4), 6.10 (d, *J* = 5.4 Hz, 1H, 4), 5.84 (d, *J* = 5.4 Hz, 1H, 3), 5.77 (d, *J* = 5.4 Hz, 1H, 3), 2.74 (hept, *J* = 7.0 Hz, 1H, 6), 2.39 (s, 3H, 1), 2.27–2.17 (m, 2H, 21), 1.76–1.69 (m, 1H, 22), 1.31 (d, *J* = 6.9 Hz, 6H, 7), 0.78 (d, *J* = 6.7 Hz, 3H, 23), 0.63 (d, *J* = 6.6 Hz, 3H, 23) ppm. ^13^C NMR (151 MHz, MeOD) *δ* 184.2 (11 or 19), 184.0 (11 or 19), 141.4 (8), 136.7 (13 or 18), 134.5 (13 or 18), 132.4 (15–17), 131.5 (15–17), 127.7 (15–17, 14 or 10), 127.5 (15–17, 14 or 10), 127.3 (15–17, 14 or 10), 109.6 (20), 109.4 (9), 98.2 (12), 90.8 (5), 89.0 (2), 74.2 (4), 73.3 (4), 70.3 (3), 70.2 (3), 33.1 (6), 32.7 (21), 29.0 (22), 23.6 (4), 23.2 (7 or 23), 23.1 (7 or 23), 22.7 (7 or 23), 18.6 (1) ppm. ESI-HR-MS [M + H^+^] *m*/*z* found: 623.1937 (623.19440), [M + Na^+^] *m*/*z* found: 645.1756 (645.17634). Elemental analysis calculated for C_27_H_30_N_2_O_3_Os: C 52.24%, H 4.87%, N 4.51%, O 7.73%. Found: C 51.88%, H 4.85%, N 4.61%, O 7.67%.

#### [(3-(*tert*-Butyl)-1-(1*H*-κ*N*2-pyrazol-1-yl)-4-oxo-1,4-dihydronaphtalene-1,2-bis(olato)-κ*O*1-κ*O*2)(η^6^-*p*-cymene)osmium(ii)]: 2d

The product was synthesized according to the general procedure, using osmium(ii) dimer (105 mg, 0.13 mmol, 1 equiv.), naphthoquinone derivative d (58 mg, 0.25 mmol, 1.9 equiv.), pyrazole (22 mg, 0.32 mmol, 2.5 equiv.) and triethylamine (105 µL, 77 mg, 0.76 mmol, 6 equiv.) in methanol (12 mL). Stirring under microwave irradiation led to formation of a red solution. The solvent was removed under reduced pressure and the crude product was purified *via* column chromatography (silica, isocratic 70% ethyl acetate and 5% triethylamine in *n*-hexane). Precipitation with DCM/*n*-hexane led to the product 2d as a yellow powder (123 mg, 0.20 mmol, 80%). ^1^H NMR (600 MHz, MeOD) *δ* 8.26 (d, *J* = 2.3, 0.7 Hz, 1H, 8), 8.07–8.01 (m, 1H, 14), 7.62–7.54 (m, 3H, 15–17), 6.84 (dd, *J* = 2.6, 0.7 Hz, 1H, 10), 6.38 (dd, *J* = 2.4, 2.4 Hz, 1H, 9), 6.17 (d, *J* = 5.4 Hz, 1H, 4), 6.09 (d, *J* = 5.4 Hz, 1H, 4), 5.83 (d, *J* = 5.3 Hz, 1H, 3), 5.74 (d, *J* = 5.4 Hz, 1H, 3), 2.72 (hept, *J* = 6.9 Hz, 1H, 6), 2.41 (s, 3H, 1), 1.30 (d, *J* = 6.9 Hz, 6H, 7), 1.28 (s, 9H, 22) ppm. ^13^C NMR (151 MHz, MeOD) *δ* 184.8 (11 or 19), 182.6 (11 or 19), 141.2 (8), 136.0 (13 or 18), 135.9 (13 or 18), 132.0 (15–17), 131.4 (15–17), 127.3 (15–17, 14 or 10), 127.2 (15–17, 14 or 10), 127.1 (15–17, 14 or 10), 116.0 (20), 109.2 (9), 98.9 (12), 90.2 (5), 89.6 (2), 74.4 (4), 73.7 (4), 69.9 (3), 35.7 (21), 33.1 (6), 31.3 (22), 23.6 (7), 23.3 (7), 19.2 (1) ppm. ESI-HR-MS [M + H^+^] *m*/*z* found: 623.1942 (623.19440), [M + Na^+^] *m*/*z* found: 645.1761 (645.17634). Elemental analysis calculated for C_27_H_30_N_2_O_3_Os·0.15 H_2_O: C 52.01%, H 4.90%, N 4.49%, O 8.08%. Found: C 51.78%, H 4.85%, N 4.55%, O 7.86%.

#### [(3-Neopentyl-1-(1*H*-κ*N*2-pyrazol-1-yl)-4-oxo-1,4-dihydronaphtalene-1,2-bis(olato)-κ*O*1-κ*O*2)(η^6^-*p*-cymene)osmium(ii)]: 2e

The product was synthesized according to the general procedure, using osmium(ii) dimer (100 mg, 0.13 mmol, 1 equiv.), naphthoquinone derivative e (59 mg, 0.24 mmol, 1.9 equiv.), pyrazole (17 mg, 0.25 mmol, 1.9 equiv.) and triethylamine (105 µL, 77 mg, 0.76 mmol, 6 equiv.) in methanol (12 mL). Stirring under microwave irradiation led to formation of a greenish solution. The solvent was removed under reduced pressure and the crude product was purified *via* column chromatography (silica, isocratic 70% ethyl acetate and 5% triethylamine in *n*-hexane). Precipitation with DCM/*n*-hexane led to the product 2e as a yellow powder (94 mg, 0.15 mmol, 63%). ^1^H NMR (600 MHz, MeOD) *δ* 8.27 (d, *J* = 2.1 Hz, 1H, 8), 8.15–8.08 (m, 1H, 14), 7.69–7.59 (m, 3H, 15–17), 6.90 (d, *J* = 2.5 Hz, 1H, 10), 6.39 (dd, *J* = 2.4, 2.4 Hz, 1H, 9), 6.16 (d, *J* = 5.4 Hz, 1H, 4), 6.11 (d, *J* = 5.4 Hz, 1H, 4), 5.84 (d, *J* = 5.4 Hz, 1H, 3), 5.73 (d, *J* = 5.4 Hz, 1H, 3), 2.74 (hept, *J* = 6.9 Hz, 1H, 6), 2.39 (s, 3H, 1), 2.35–2.27 (m, 2H, 21), 1.32 (d, *J* = 6.9 Hz, 6H, 7), 0.69 (s, 9H, 23) ppm. ^13^C NMR (151 MHz, MeOD) *δ* 184.6 (11 or 19), 184.3 (11 or 19), 141.4 (8), 136.6 (13 or 18), 134.6 (13 or 18), 132.3 (15–17), 131.5 (15–17), 127.6 (15–17, 14 or 10), 127.4 (15–17, 14 or 10), 127.4 (15–17, 14 or 10), 109.4 (9), 108.4 (20), 98.4 (12), 90.4 (5), 89.0 (2), 74.4 (4), 73.5 (4), 70.3 (3), 70.2 (3), 36.1 (21), 34.2 (22), 33.1 (6), 30.5 (23), 23.6 (7), 23.2 (7), 18.8 (1) ppm. ESI-HR-MS [M + H^+^] *m*/*z* found: 637.2091 (637.21005), [M + Na^+^] *m*/*z* found: 659.1910 (659.19199). Elemental analysis calculated for C_28_H_32_N_2_O_3_Os·0.15 H_2_O: C 52.75%, H 5.11%, N 4.39%, O 7.91%. Found: C 52.45%, H 5.03%, N 4.52%, O 7.57%.

#### [(3-(2-Ethylbutyl)-1-(1*H*-κ*N*2-pyrazol-1-yl)-4-oxo-1,4-dihydronaphtalene-1,2-bis(olato)-κ*O*1-κ*O*2)(η^6^-*p*-cymene)osmium(ii)]: 2f

The product was synthesized according to the general procedure, using osmium(ii) dimer (99 mg, 0.126 mmol, 1 equiv.), naphthoquinone derivative f (62 mg, 0.24 mmol, 1.9 equiv.), pyrazole (16 mg, 0.24 mmol, 1.9 equiv.) and triethylamine (106 µL, 77 mg, 0.76 mmol, 6 equiv.) in methanol (12 mL). Stirring under microwave irradiation led to formation of a red solution. The solvent was removed under reduced pressure and the crude product was purified *via* column chromatography (silica, isocratic 70% ethyl acetate and 5% triethylamine in *n*-hexane). Precipitation with DCM/*n*-hexane led to the product 2f as a yellow powder (139 mg, 0.214 mmol, 89%). ^1^H NMR (600 MHz, MeOD) *δ* 8.28 (d, *J* = 2.1 Hz, 1H, 8), 8.15–8.08 (m, 1H, 14), 7.67–7.61 (m, 3H, 15–17), 6.87 (d, *J* = 2.5 Hz, 1H, 10), 6.39 (dd, *J* = 2.4, 2.4 Hz, 1H, 9), 6.17 (d, *J* = 5.5 Hz, 1H, 4), 6.10 (d, *J* = 5.4 Hz, 1H, 4), 5.84 (d, *J* = 5.5 Hz, 1H, 3), 5.76 (d, *J* = 5.4 Hz, 1H, 3), 2.74 (hept, *J* = 6.9 Hz, 1H, 6), 2.39 (s, 3H, 1), 2.33–2.23 (m, 2H, 21), 1.43–1.38 (m, 1H, 22), 1.33–1.30 (m, 6H, 7), 1.21–1.15 (m, 2H, 23), 1.08–1.00 (m, 2H, 23), 0.79 (t, *J* = 7.5 Hz, 3H, 24), 0.73 (t, *J* = 7.5 Hz, 3H, 24) ppm. ^13^C NMR (151 MHz, MeOD) *δ* 184.2 (11 or 19), 183.9 (11 or 19), 141.4 (8), 136.7 (13 or 18), 134.6 (13 or 18), 132.3 (15–17), 131.5 (15–17), 127.7 (15–17, 14 or 10), 127.4 (15–17, 14 or 10), 127.3 (15–17, 14 or 10), 109.8 (20), 109.4 (9), 98.3 (12), 90.6 (5), 89.1 (2), 74.3 (4), 73.4 (4), 70.2 (3), 70.2 (3), 41.2 (22), 33.1 (6), 27.8 (21), 26.7 (23), 26.3 (23), 23.7 (7), 23.2 (7), 18.7 (1), 11.6 (24), 11.2 (24) ppm. ESI-HR-MS [M + H^+^] *m*/*z* found: 651.2249 (651.22570), [M + Na^+^] *m*/*z* found: 673.2070 (673.20764). Elemental analysis calculated for C_29_H_34_N_2_O_3_Os: C 53.68%, H 5.28%, N 4.32%, O 7.40%. Found: C 53.70%, H 5.26%, N 4.43%, O 7.53%.

## Conclusions

A series of twelve organoruthenium and organoosmium tridentate complexes was synthesized and characterized *via* 1D- and 2D-NMR, HRMS and X-ray crystallography. Variation of electron inducing alkyl substituents on the *in situ* formed tridentate ligand proposedly results in a more stable complex formation and overall altered physicochemical and biological properties. Stability enhancement was determined *via* incubation in aqueous solution and isolation of intact complex at certain time points *via* UHPLC. Osmium derivatives showed overall higher inertness than ruthenium equivalents. Higher branched substituents proved to support complex stability, with peak values found for ruthenium *tert* butyl derivative 1d and osmium neopentyl complex 2e. MTT assays reconfirmed the outstanding cytotoxicity of this compound class. Moreover, the known *in vitro* selectivity pattern for SW480 colon cancer cells was corroborated (Table 2). Cellular accumulation was measured for ruthenium-based derivatives and showed clear correlation of higher cellular uptake to lower IC_50_ values in SW480 cells. Strikingly, higher lipophilicity does not correlate with enhanced accumulation, although it usually promotes membrane permeability. Furthermore, longer and branched substituents on the tridentate scaffold result in lower cellular uptake, alongside reduced cytotoxicity. Overall, steric demand on the naphthoquinone scaffold seems to impact bioavailability of this compound class. In contrast to earlier beliefs, enhanced stability does not reflect strongly in *in vitro* results of this compound class. As depicted in Fig. 4, metal center variation to osmium strongly favors stability in aqueous solution (2a, 2b, 2c, 2e). However, IC_50_ values are not far off their ruthenium counterparts, and clearly appear to be more reliant on choice of naphthoquinones a–e. Inhibition of cell cycle progression was analyzed for Ru-Ethyl, 1a, 1c, Os-Ethyl, 2a, and 2c. Pronounced effects were only found for ruthenium-based derivatives, while SW480 cells showed higher sensitivity than CH1/PA-1 cells. For ruthenium complexes, most prominently Ru-Ethyl, a strong decrease in G0/G1 phase, alongside cell cycle arrest in G2/M and S phases was found. Based on this data, choice of the metal center seems to play a role, in favor of ruthenium. Nevertheless, this does not fully reflect in IC_50_ values. Ru-Ethyl was identified as a potential IDO1 inhibitor. Significant inhibition was found in SKOV3 cells, yet it could only be attributed to this certain derivative. However, these results exemplify the multimodal, yet hardly predictable impact of *N*,*O*,*O* tridentate complexes on cancer cell metabolism. Hence, these first investigations on IDO1 might pave the way for a broader investigation on immunomodulation by Ru-Ethyl. Evidently, choice of the naphthoquinone derivative is a decisive factor not only for complex stability, but essentially for *in vitro* bioavailability. It could therefore play a critical role in an *in vivo* setting. Therefore, we suggest further studies in cellular and animal models, to review current data. In upcoming studies, Ru-Ethyl might be validated as the most promising derivative of *N*,*O*,*O*-tridentate complexes.

## Author contributions

Conceptualization, A. R. and W. K.; methodology, A. R., W. K. and M. A. J., P. H., W. B.; validation A. R., A. P.-R., M. A. J., P. H., W. B. and W. K.; formal analysis, A. R., H. G., M. H., M. G., A. R.-P., A. L., M. A. J. and W. K.; investigation, A. R., H. G., M. H., M. G., A. R.-P., A. L. and M. A. J.; resources, P. H., W. B., W. K., M. A. J. and B. K. K.; data curation, A. R., A. R.-P., A. L., P. H., M. A. J and W. K.; writing – original draft preparation, A. R., A. P.-R., M. A. J., P. H. and W. K.; writing – review and editing, A. R., W. K., and M. A. J.; visualization, A. R., M. G., A. P.-R., A. L., W. K. and M. A. J.; supervision, M. A. J., P. H., W. B., W. K., and B. K. K.; project administration, W. K.; funding acquisition, W. K., M. A. J., P. H., and B. K. K.; all authors have read and agreed to the published version of the manuscript.

## Conflicts of interest

There are no conflicts to declare.

## Supplementary Material

DT-055-D5DT01649E-s001

DT-055-D5DT01649E-s002

## Data Availability

The data supporting this article have been included as part of the supplementary information (SI). Supplementary information: NMR spectra, MS spectra, X-crystallography data, HPLC chromatograms and cell culture experiments. See DOI: https://doi.org/10.1039/d5dt01649e. CCDC 2422945, 2422947–2422950, 2448265, 2448267, 2448268, 2448913, 2469755–2469757 contain the supplementary crystallographic data for this paper.^45*a*–*l*^
