# Supplementary material for: Naphthoquinone-derived tridentate Ru(ii) and Os(ii) organometallics with exceptional cytotoxicity: synthesis, characterization, stability in aqueous solution and biological in vitro evaluation
Source: Dalton Trans. 2026 Jan 20;55(5):2254–68. doi: 10.1039/d5dt01649e (PMC12818348; doi:10.1039/d5dt01649e)
Supplement: DT-055-D5DT01649E-s001 [file DT-055-D5DT01649E-s001.pdf]

Supplementary Information (SI) for Dalton Transactions.
This journal is © The Royal Society of Chemistry 2026

Supporting Information

Naphthoquinone-derived tridentate Ru(II) and Os(II) organometallics with exceptional cytotoxicity: Synthesis, characterization, stability in aqueous solution and biological *in vitro* evaluation

Alexander Rosner,[a,b] Heiko Geisler,[a] Michaela Hejl,[a] Mathias Gradl,[c,d] Anton A. Legin,[a] Alexander Roller,[a] Michael A. Jakupec,[a,c] Petra Heffeter,[c,d] Walter Berger,[c,d] Bernhard K. Keppler,[a,c] Wolfgang Kandioller[a,c,*]

[a]Institute of Inorganic Chemistry, Faculty of Chemistry, University of Vienna, Währinger Str. 42, 1090 Vienna, Austria.

[b]Vienna Doctoral School in Chemistry (DoSChem), Faculty of Chemistry, University of Vienna, Währinger Str. 42, 1090 Vienna, Austria.

[c]Research Cluster "Translational Cancer Therapy Research", Währinger Str. 42, 1090 Vienna, Austria.

[d]Center for Cancer Research and Comprehensive Cancer Center, Medical University of Vienna, Austria.

*wolfgang.kandioller@univie.ac.at, +43-1-4277-52609

Table of Content

1 NMR Spectra 2

2 Mass spectra 20

3 X-ray crystallography data 32

4 HPLC-MS 56

5 Cell Culture 61

5.1 Cell Viability Assay (MTT Assay) 61

5.2 Cellular accumulation 64

5.3 Cell Cycle Studies 64

6 Appendix 67

1 NMR Spectra

1.1.1 2-Propyl-3-hydroxynaphthalene-1,4-dione: **a**

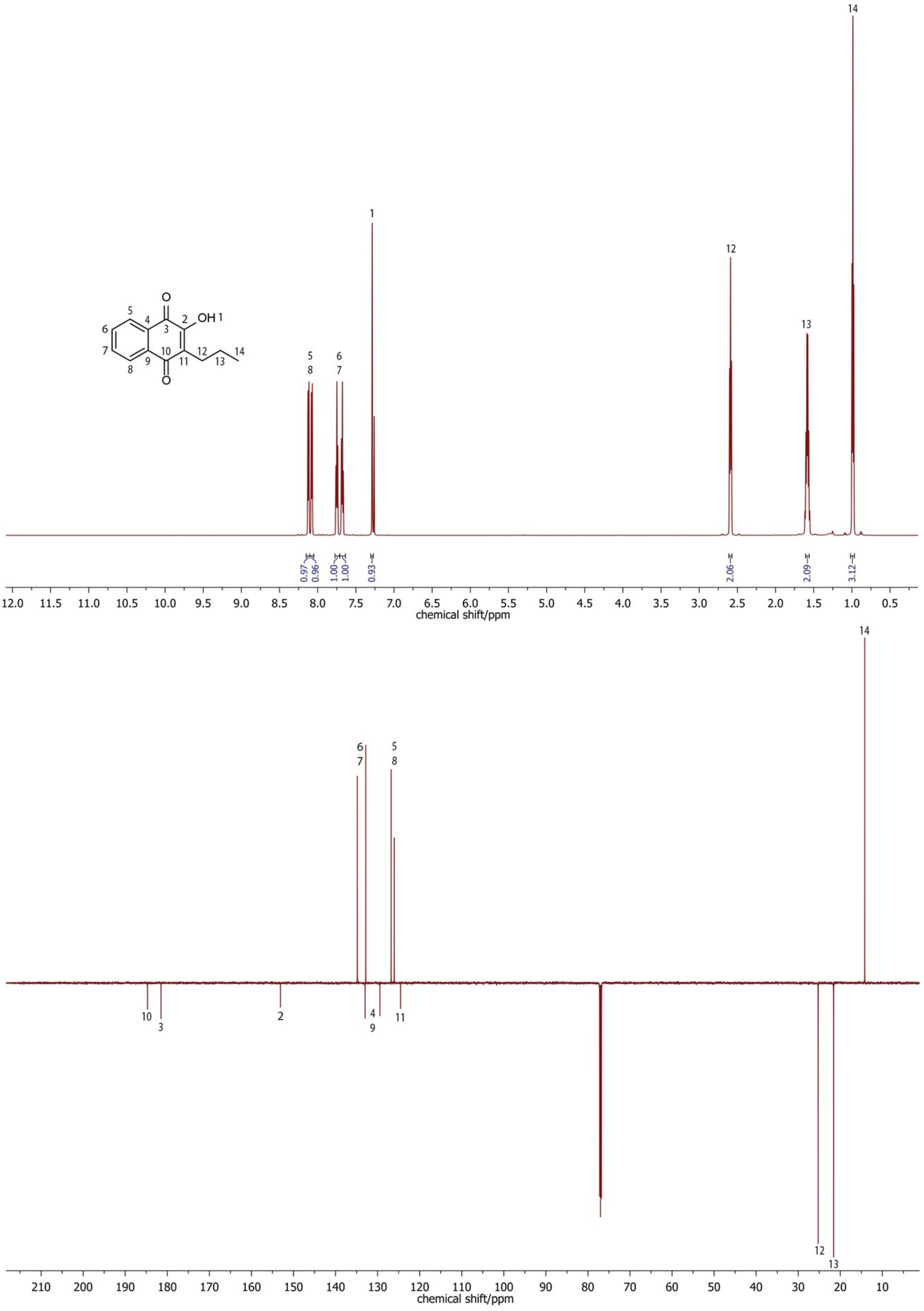

Figure S1: ^{1}H (above) and ^{13}C (below) NMR spectra of ligand **a** in $CDCl_3$.

1.1.2 2-Hydroxy-3-propylnaphthalene-1,4-dione: **b**

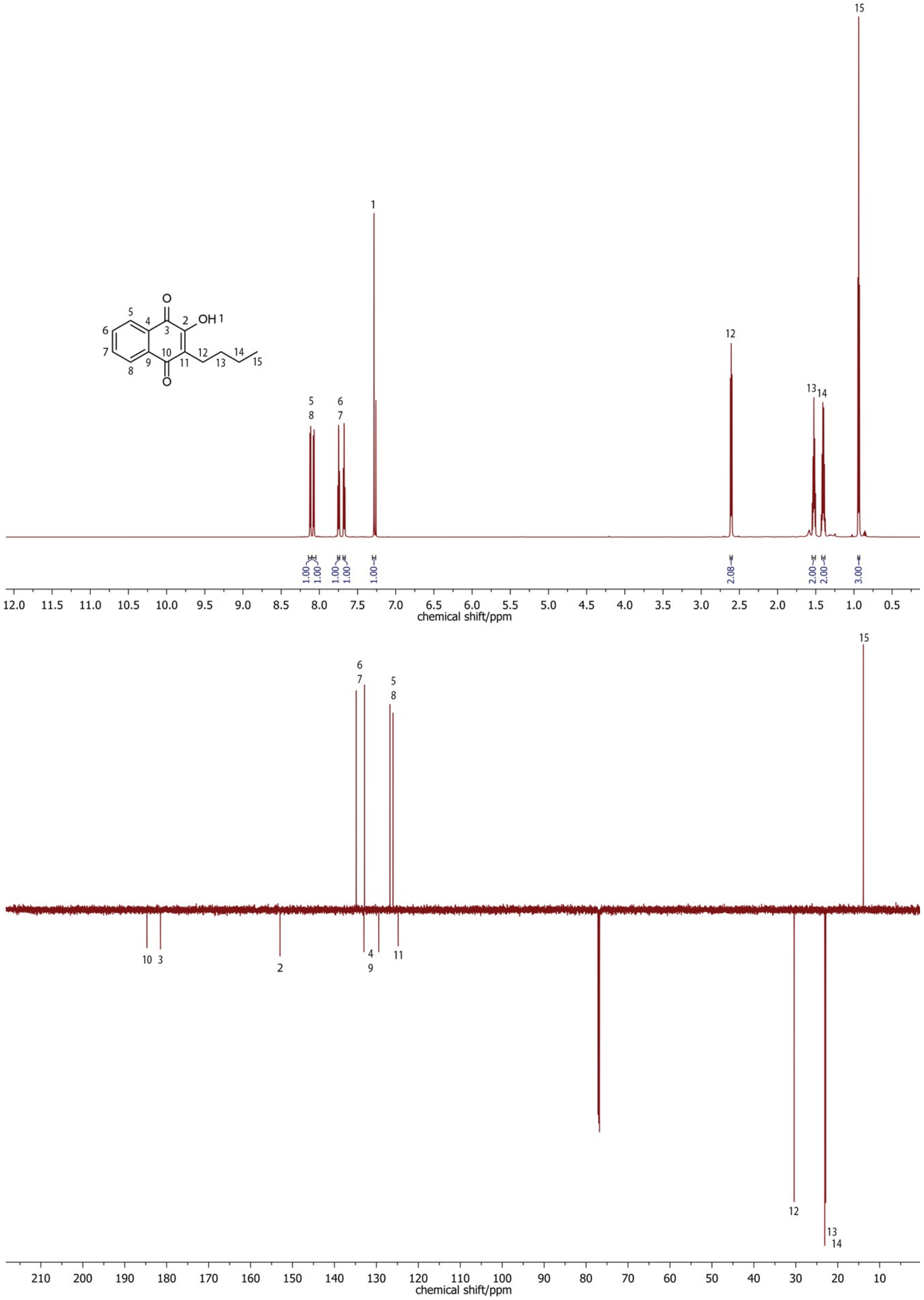

Figure S2: ^{1}H (above) and ^{13}C (below) NMR spectra of ligand **b** in $CDCl_3$.

1.1.3 2-Hydroxy-3-isobutylnaphthalene-1,4-dione: **c**

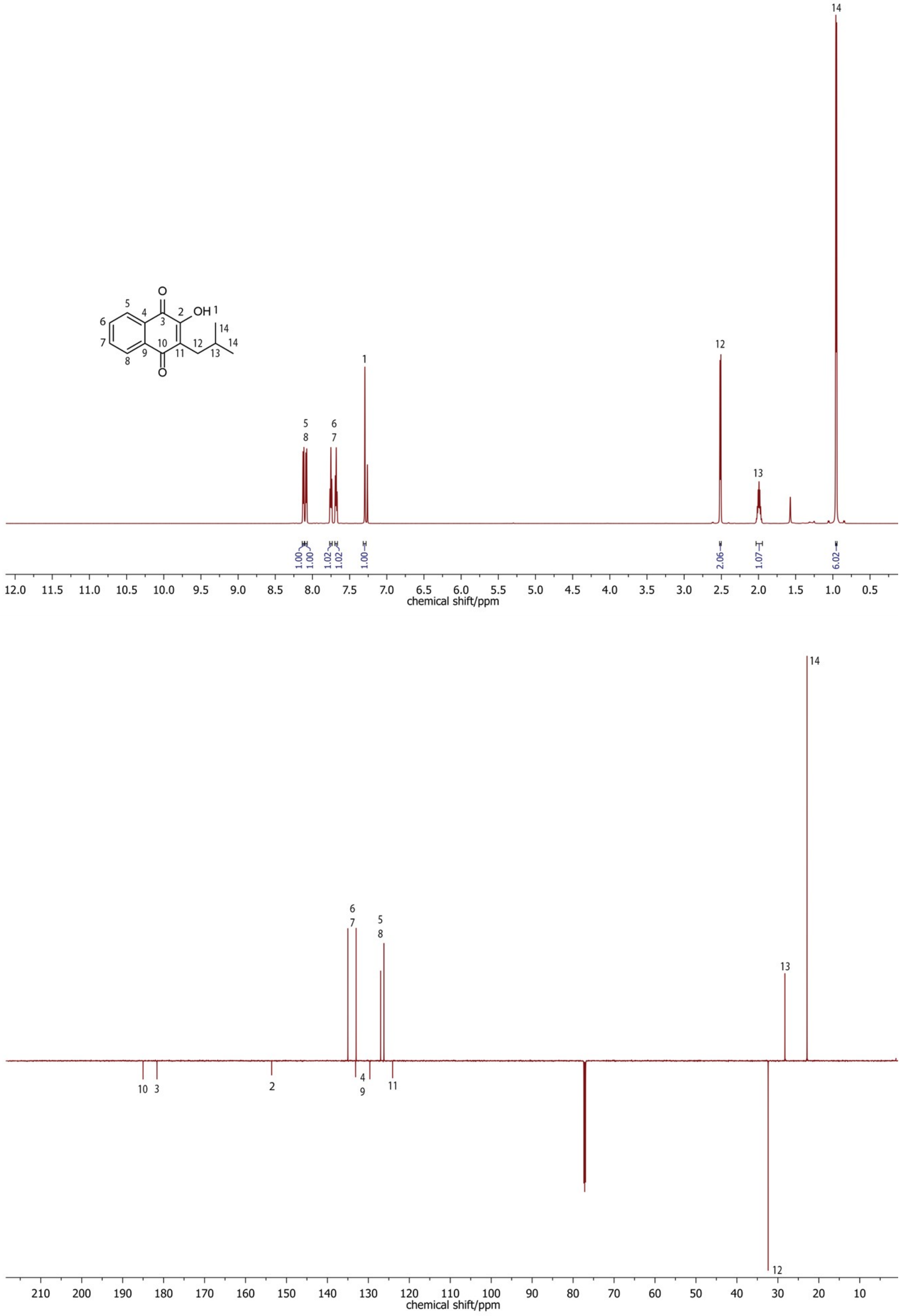

Figure S3: ^{1}H (above) and ^{13}C (below) NMR spectra of ligand **c** in $CDCl_3$.

1.1.4 2-(tert-Butyl)-3-hydroxynaphthalene-1,4-dione: **d**

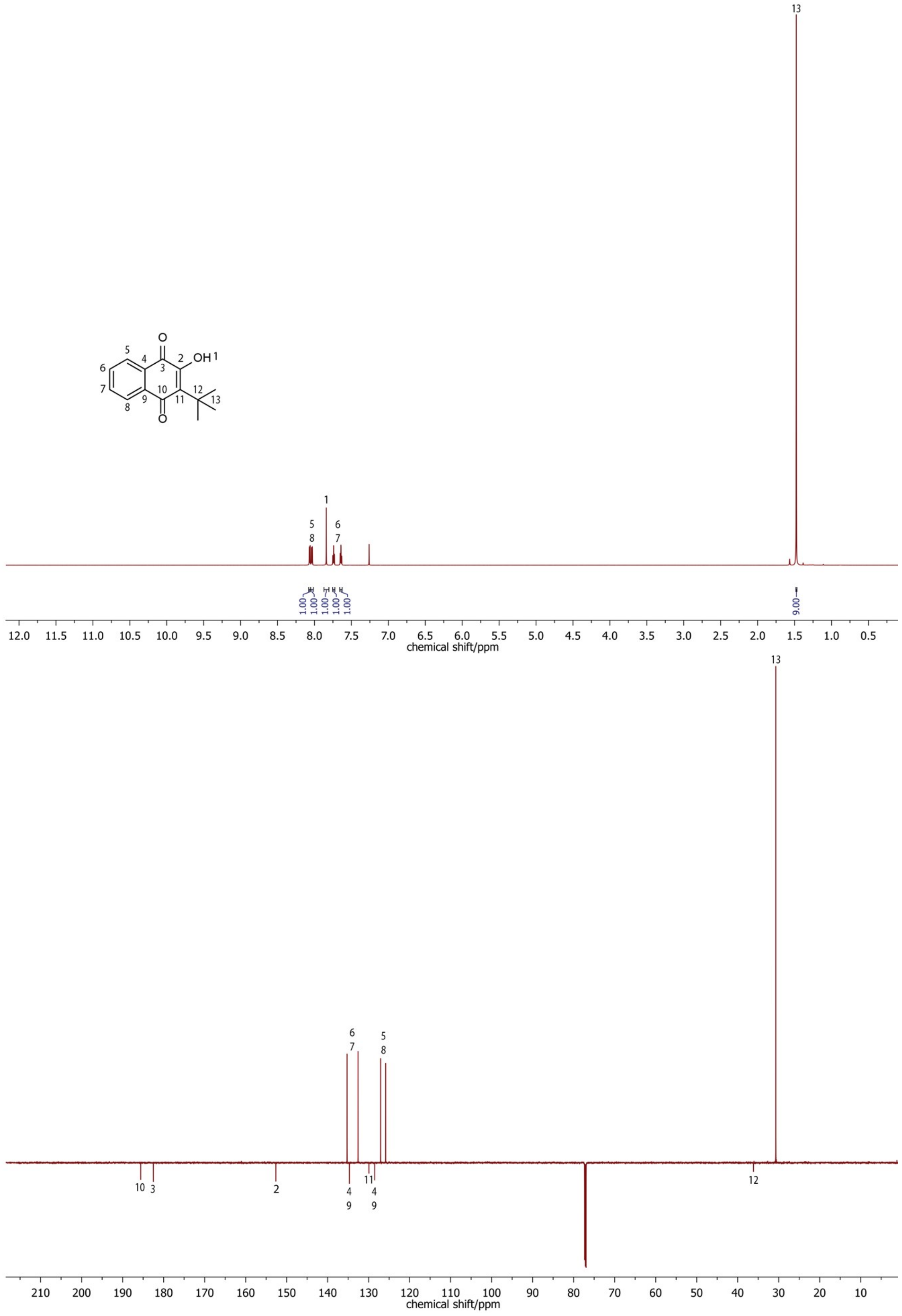

Figure S4: ^{1}H (above) and ^{13}C (below) NMR spectra of ligand **d** in $CDCl_3$.

1.1.5 2-Hydroxy-3-neopentylnaphthalene-1,4-dione: **e**

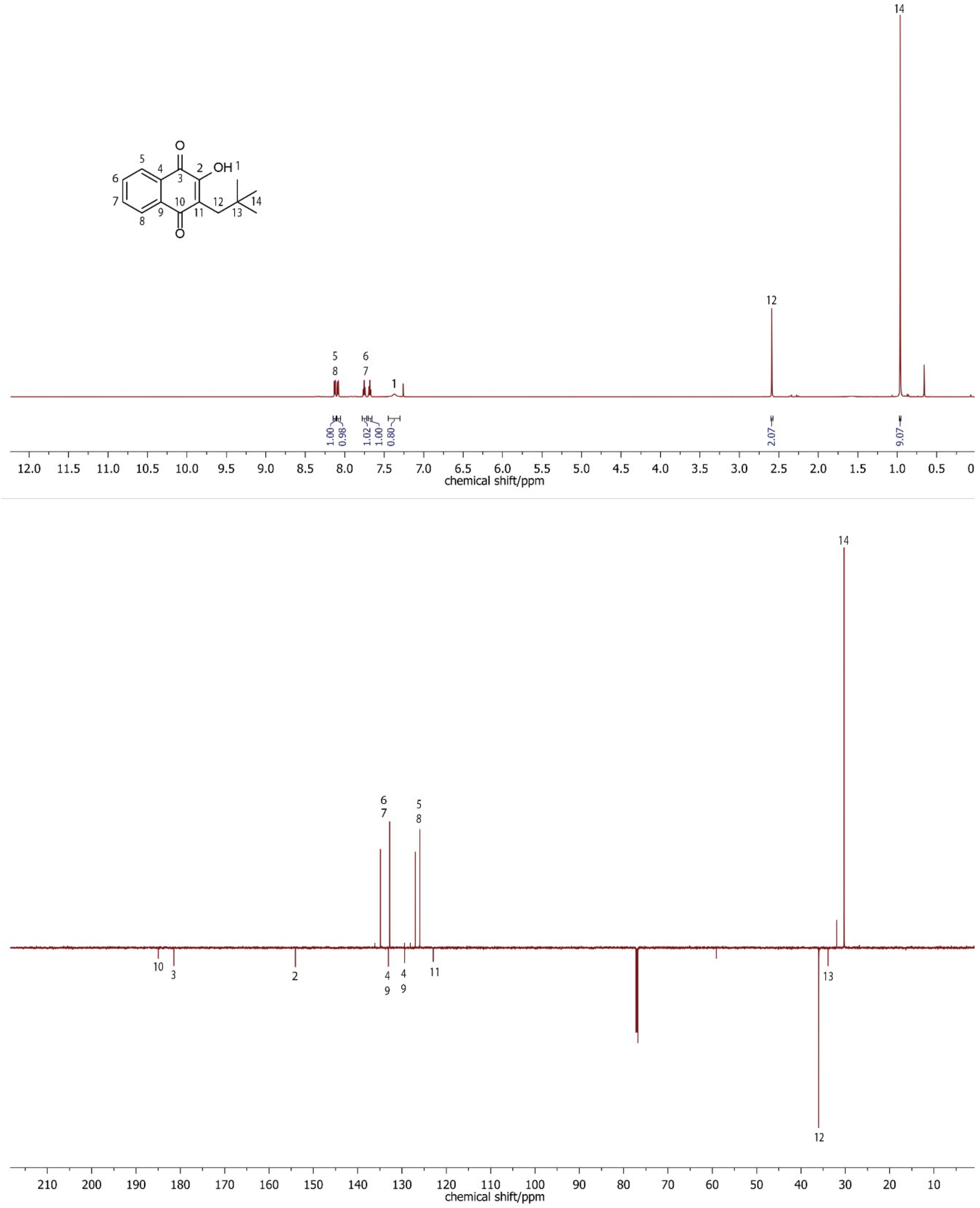

Figure S5: ^{1}H (above) and ^{13}C (below) NMR spectra of ligand **e** in $CDCl_3$.

1.1.6 2-(2-Ethylbutyl)-3-hydroxynaphthalene-1,4-dione: **f**

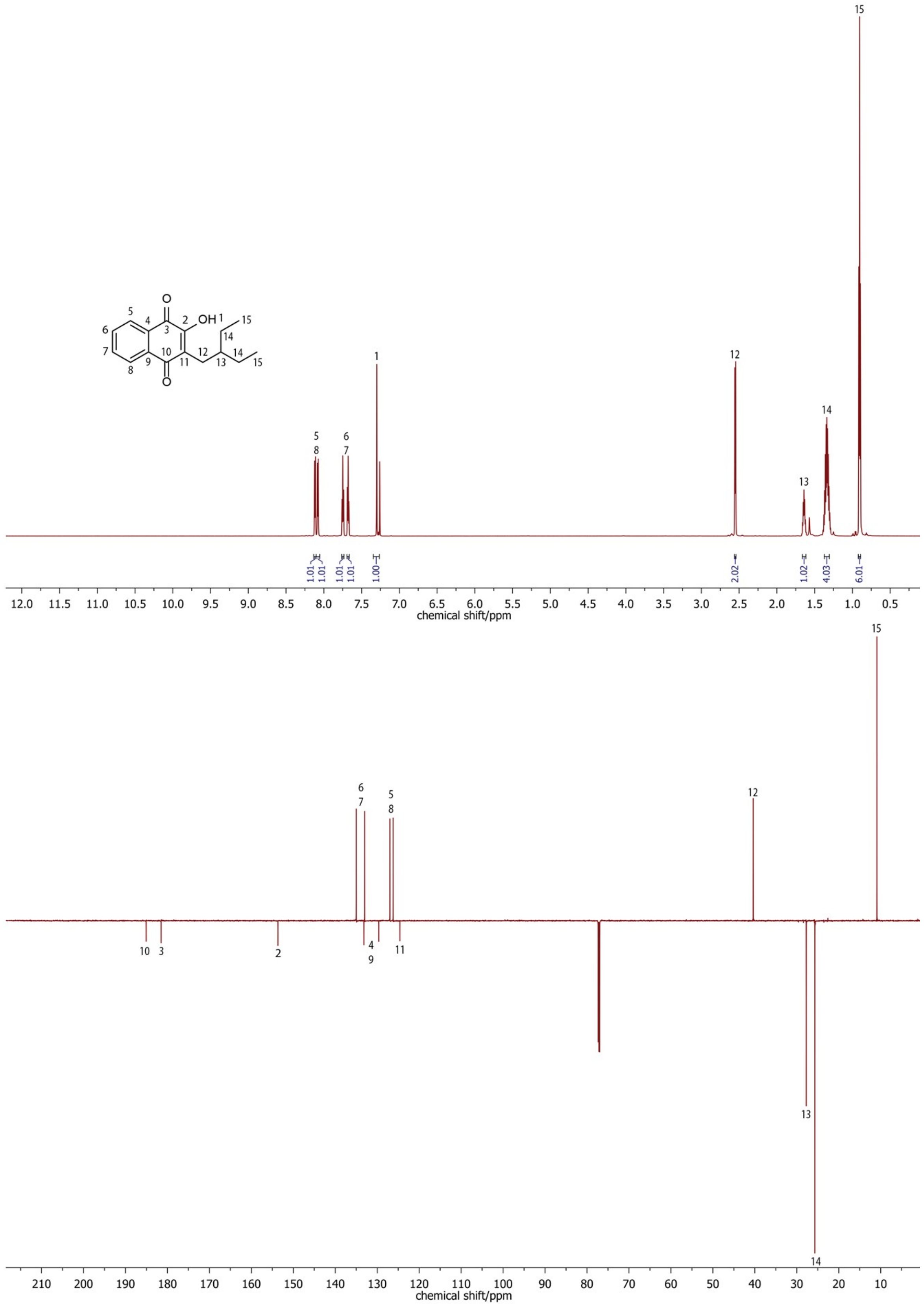

Figure S6: ^{1}H (above) and ^{13}C (below) NMR spectra of ligand **f** in $CDCl_3$.

1.1.7 [(3-Propyl-1-(1H-κ*N*2-pyrazol-1-yl)-4-oxo-1,4-dihydronaphtalene-1,2-bis(olato)-κ*O*1-κ*O*2)(η^6-*p*-cymene)ruthenium(II)]: **1a**

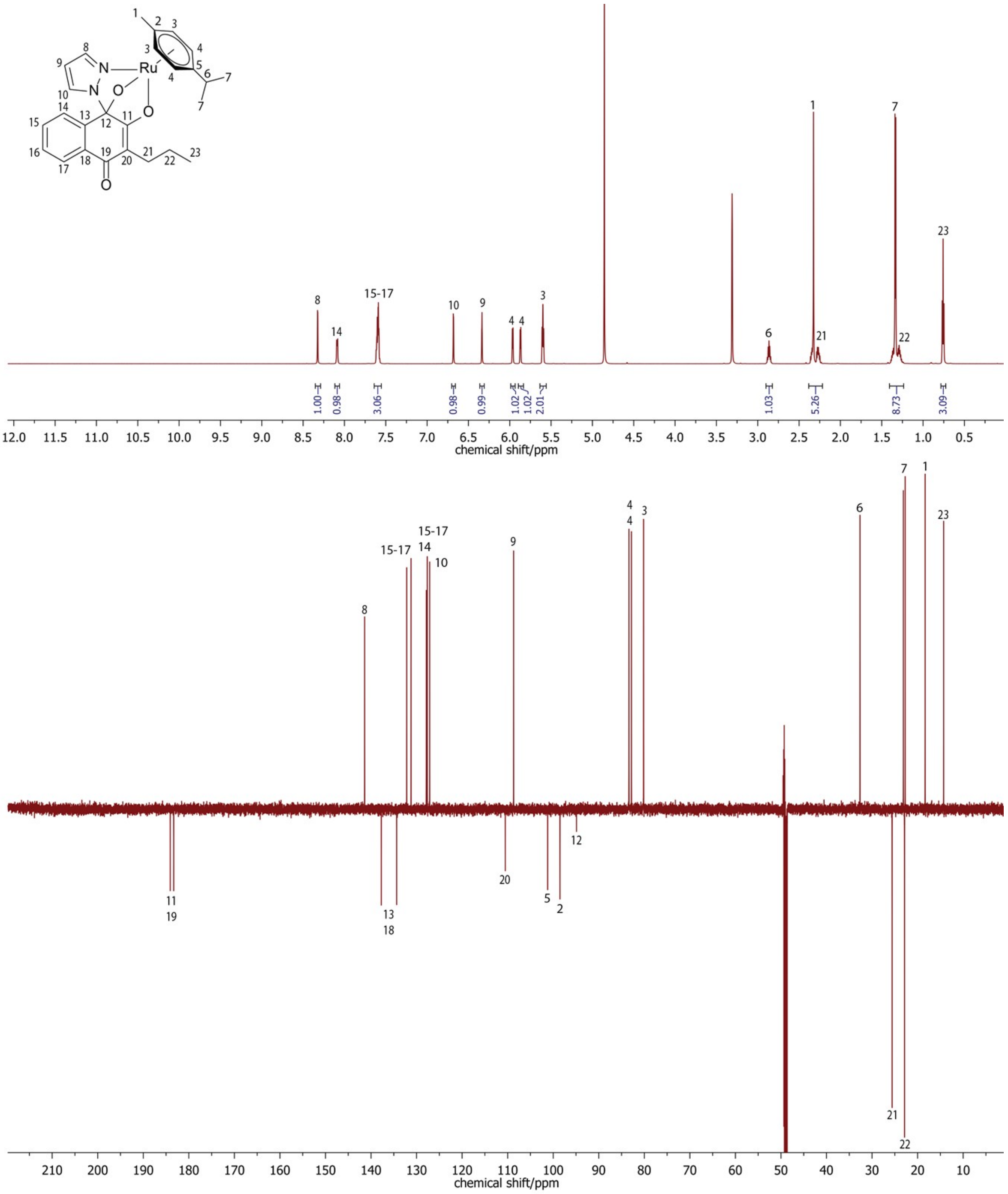

Figure S7: ^{1}H (above) and ^{13}C (below) NMR spectra of complex **1a** in d_4-MeOD.

1.1.8 [(3-Butyl-1-(1H-κ*N*2-pyrazol-1-yl)-4-oxo-1,4-dihydronaphtalene-1,2-bis(olato)-κ*O*1-κ*O*2)(η^6-*p*-cymene)ruthenium(II)]: **1b**

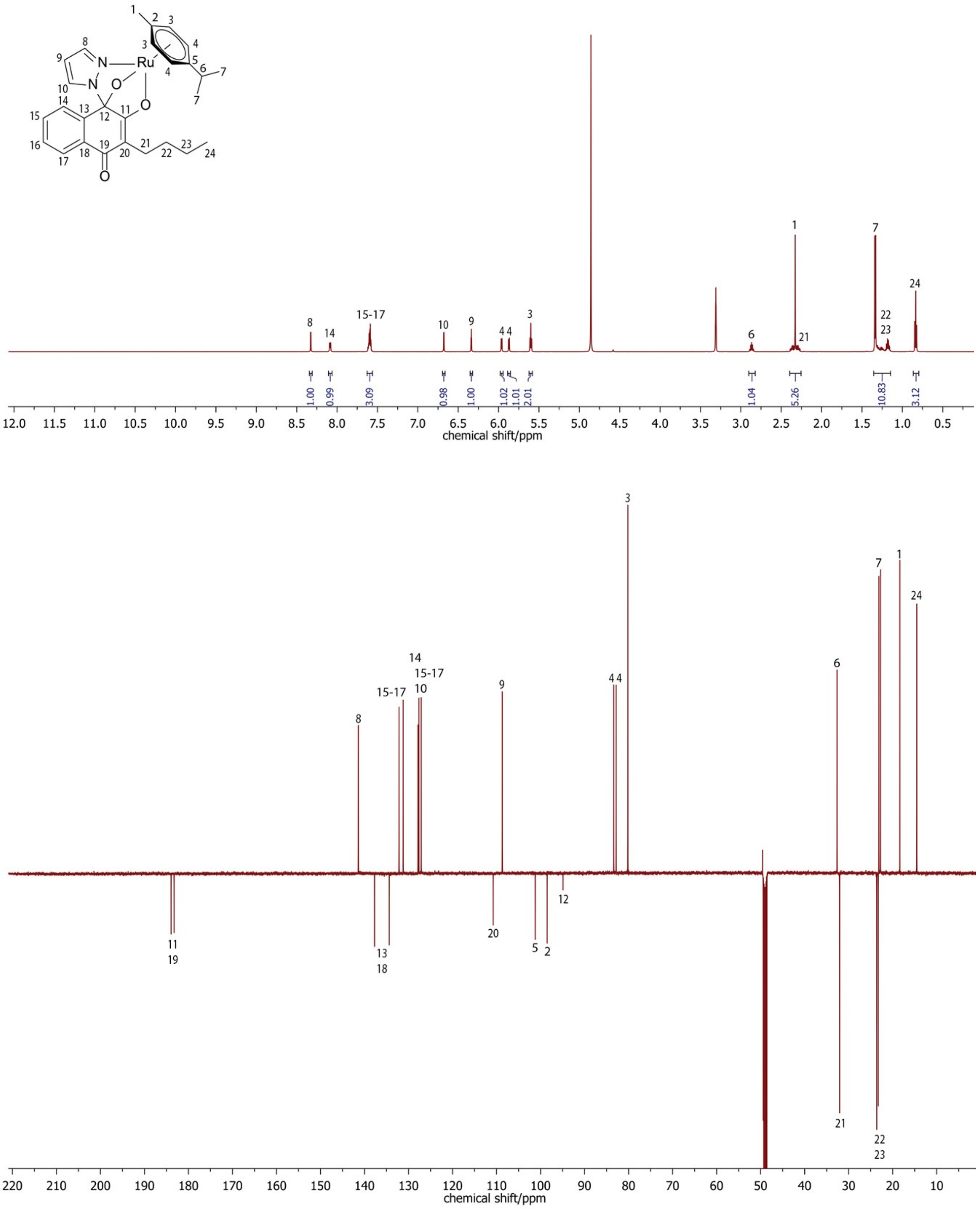

Figure S8: ^{1}H (above) and ^{13}C (below) NMR spectra of complex **1b** in d_4-MeOD.

1.1.9 [(3-Isobutyl-1-(1H-κ*N*2-pyrazol-1-yl)-4-oxo-1,4-dihydronaphtalene-1,2-bis(olato)-κ*O*1-κ*O*2)(η^6-*p*-cymene)ruthenium(II)]: **1c**

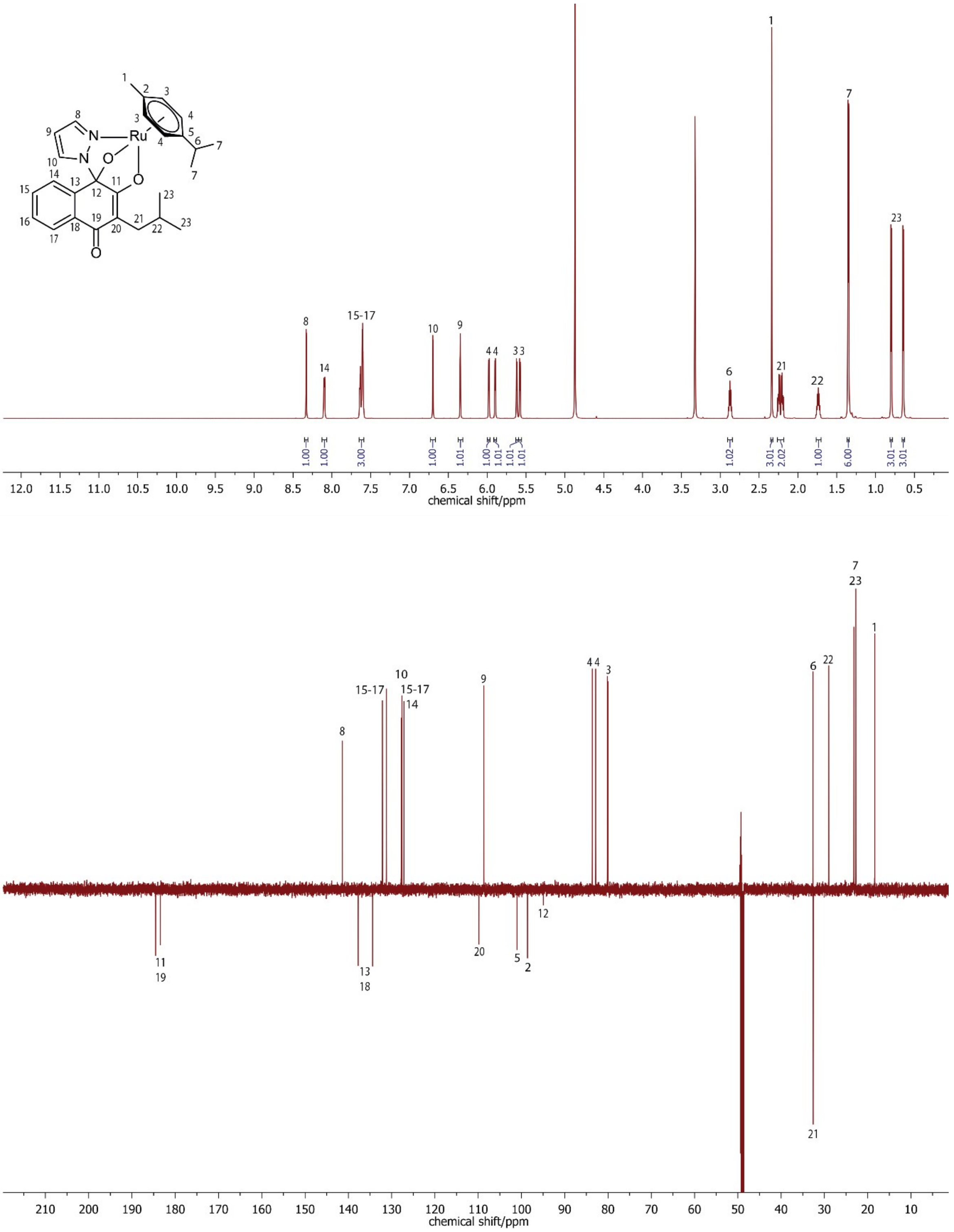

Figure S9: ^{1}H (above) and ^{13}C (below) NMR spectra of complex **1c** in d^{4}-MeOD.

1.1.10 [(3-(tert-butyl)-1-(1H-κN2-pyrazol-1-yl)-4-oxo-1,4-dihydronaphtalene-1,2-bis(olato)-κO1-κO2)(η6-p-cymene)ruthenium(II)]: **1d**

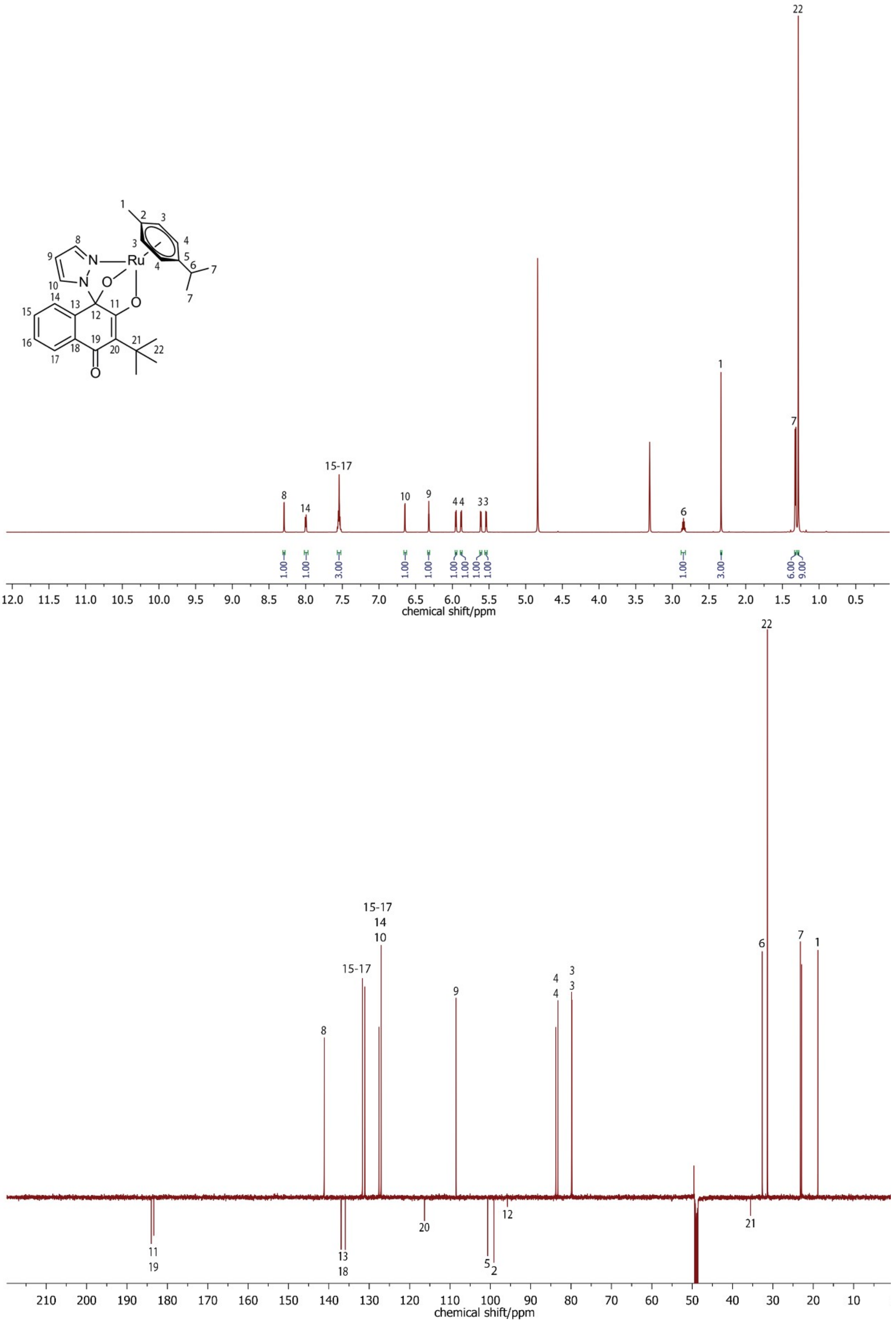

Figure S10: ^{1}H (above) and ^{13}C (below) NMR spectra of complex **1d** in d_4-MeOD.

1.1.11 [(3-Neopentyl-1-(1H-κ*N*2-pyrazol-1-yl)-4-oxo-1,4-dihydronaphtalene-1,2-bis(olato)-κ*O*1-κ*O*2)(η^6-*p*-cymene)ruthenium(II)]: **1e**

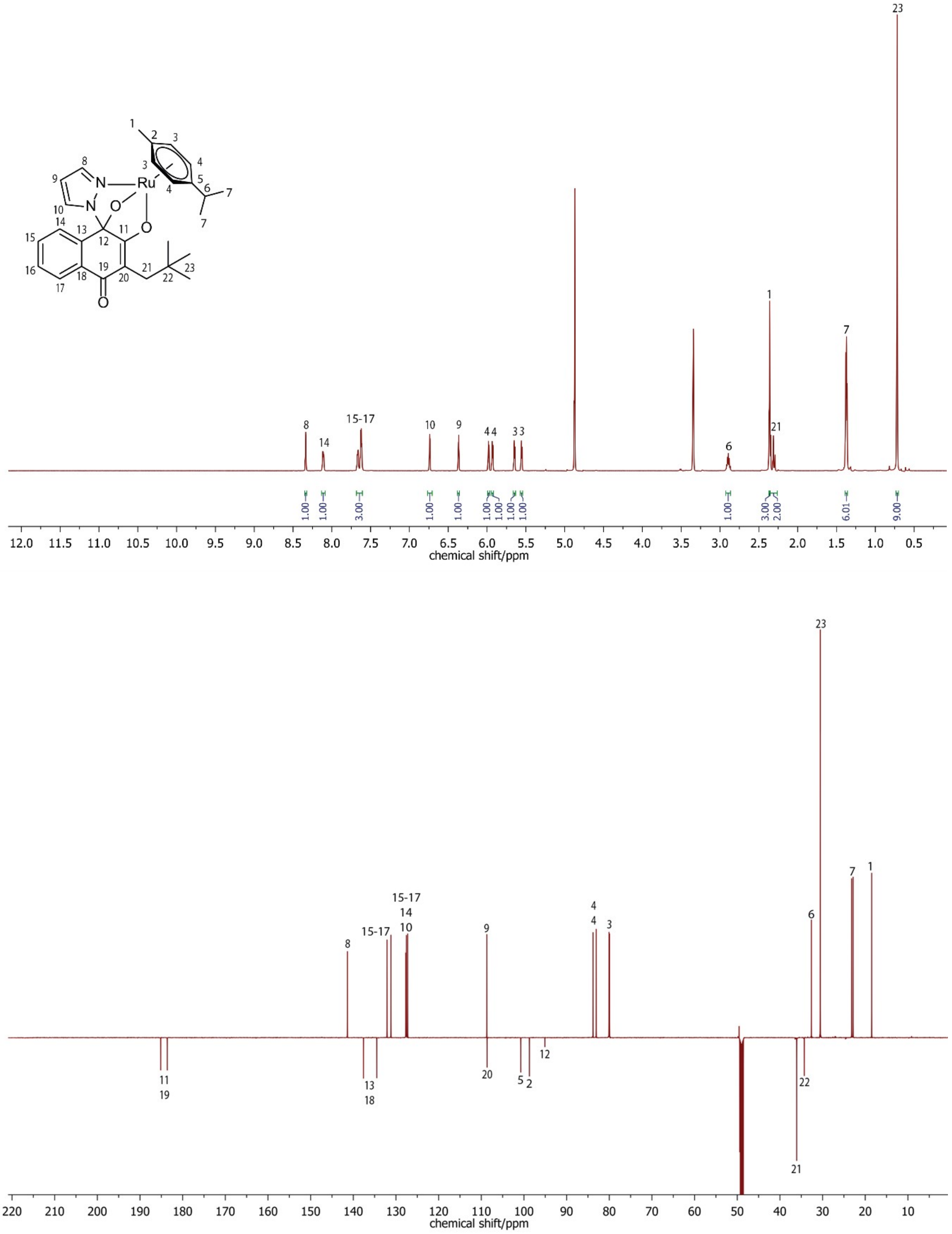

Figure S11: ^{1}H (above) and ^{13}C (below) NMR spectra of complex **1e** in d^4-MeOD.

1.1.12 [(3-(2-Ethylbutyl)-1-(1H-κN2-pyrazol-1-yl)-4-oxo-1,4-dihydronaphtalene-1,2-bis(olato)-κO1-κO2)(η6-p-cymene)ruthenium(II)]: **1f**

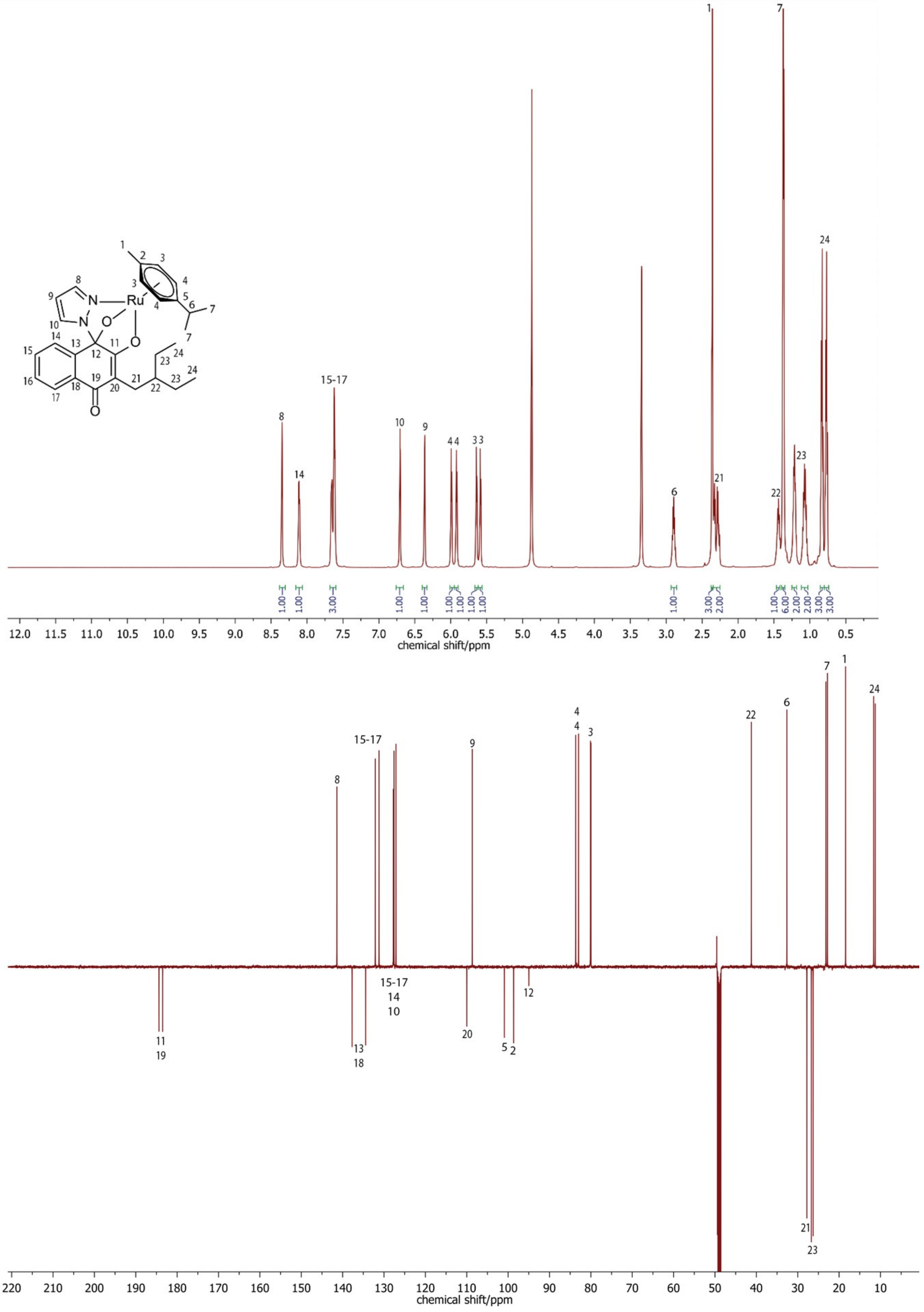

1

Figure S12: ^{1}H (above) and ^{13}C (below) NMR spectra of complex **1f** in d^{4}-MeOD.

1.1.13 [(3-Propyl-1-(1H-κ*N*2-pyrazol-1-yl)-4-oxo-1,4-dihydronaphtalene-1,2-bis(olato)-κ*O*1-κ*O*2)(η^6-*p*-cymene)osmium(II)]: **2a**

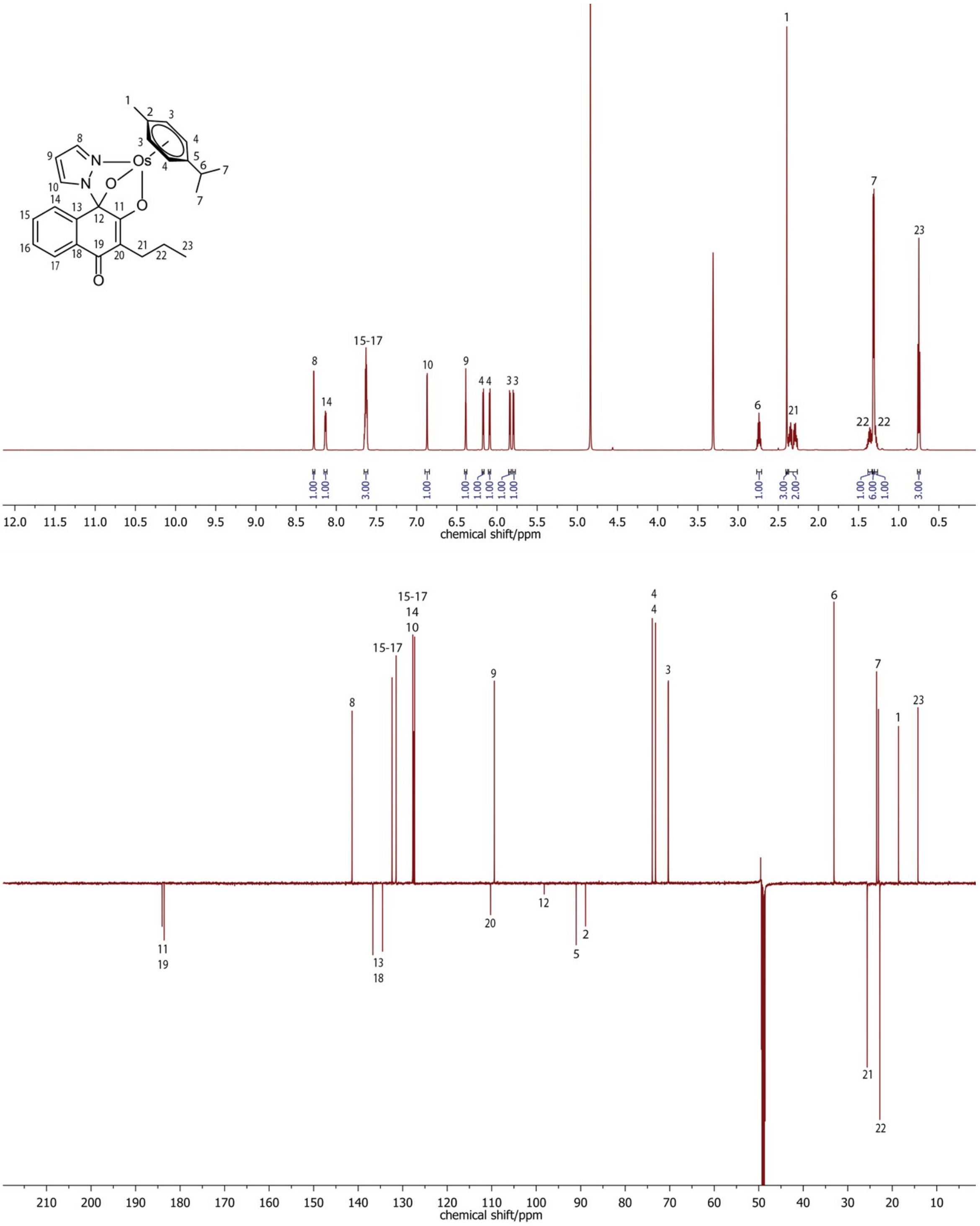

Figure S13: ^{1}H (above) and ^{13}C (below) NMR spectra of complex **2a** in d_4-MeOD.

1.1.14 [(3-Butyl-1-(1H-κ*N*2-pyrazol-1-yl)-4-oxo-1,4-dihydronaphtalene-1,2-bis(olato)-κ*O*1-κ*O*2)(η^6-*p*-cymene)osmium(II)]: **2b**

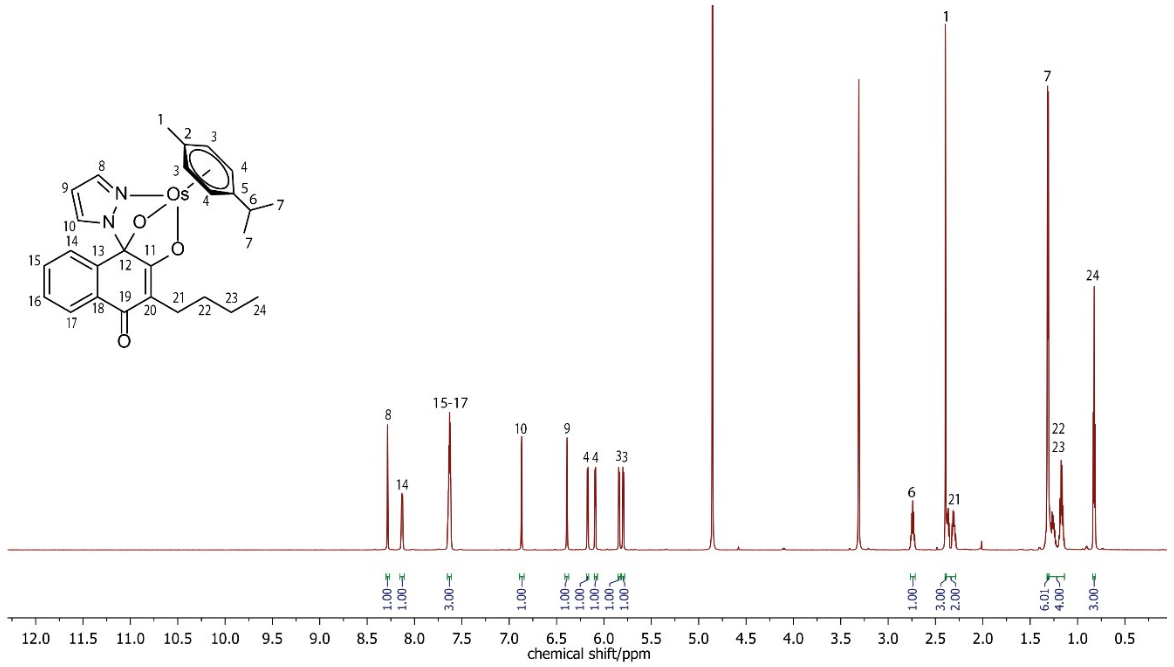

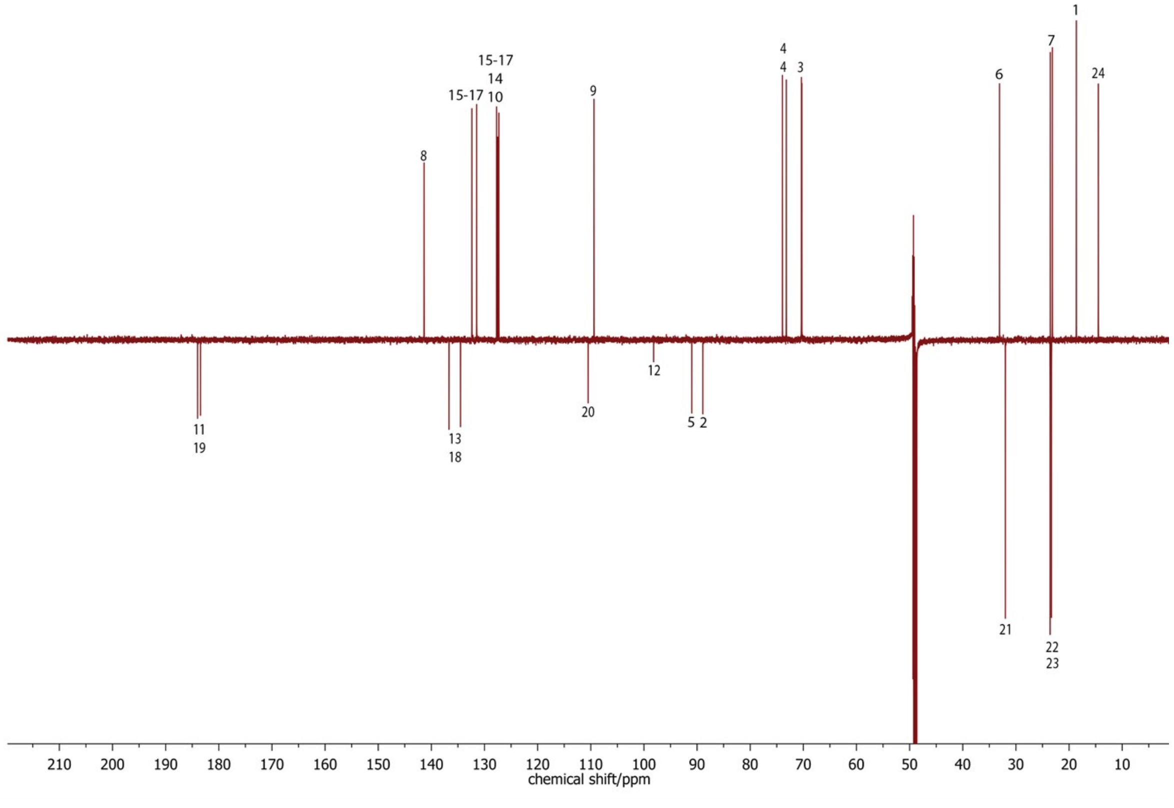

Figure S14: ^{1}H (above) and ^{13}C (below) NMR spectra of complex **2b** in d_4-MeOD.

1.1.15 [(3-Isobutyl-1-(1H-κ*N*2-pyrazol-1-yl)-4-oxo-1,4-dihydronaphtalene-1,2-bis(olato)-κ*O*1-κ*O*2)(η^6-*p*-cymene)osmium(II)]: **2c**

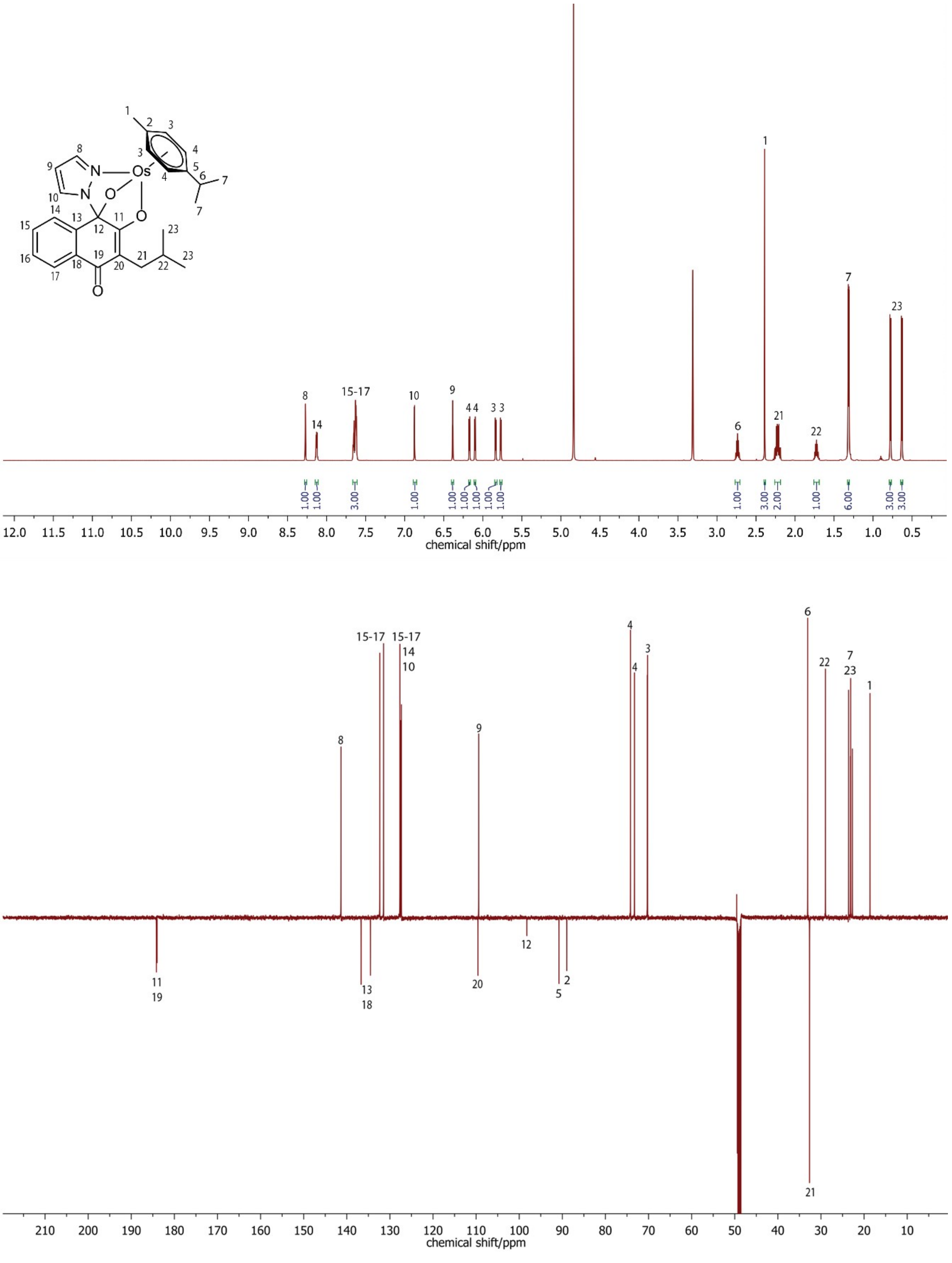

Figure S15: ^{1}H (above) and ^{13}C (below) NMR spectra of complex **2c** in d_4-MeOD.

1.1.16 [(3-(*tert*-butyl)-1-(1H-κ*N*2-pyrazol-1-yl)-4-oxo-1,4-dihydronaphtalene-1,2-bis(olato)-κ*O*1-κ*O*2)(η^6-*p*-cymene)osmium(II)]: **2d**

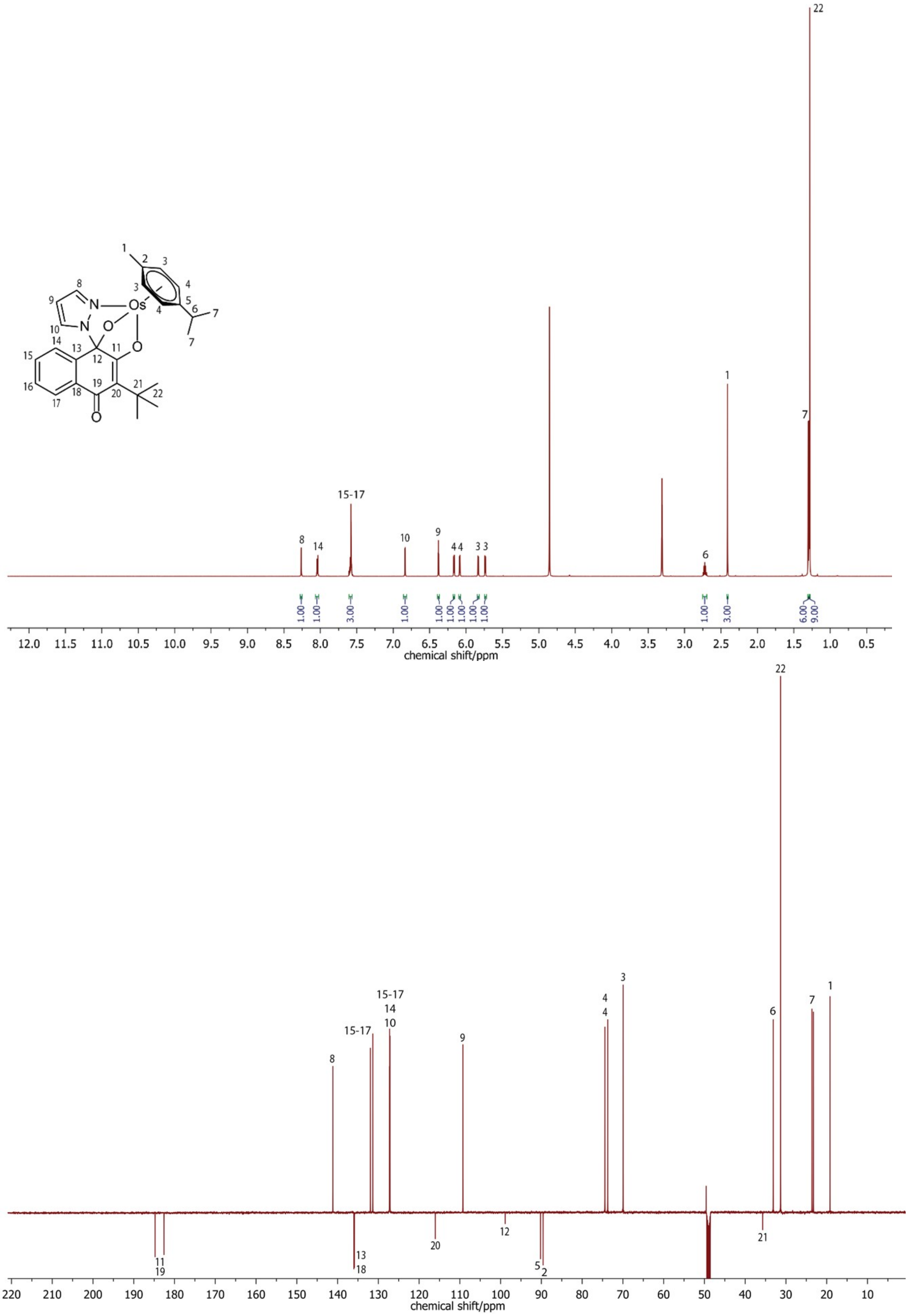

Figure S16: ^{1}H (above) and ^{13}C (below) NMR spectra of complex **2d** in d_4-MeOD.

1.1.17 [(3-Neopentyl-1-(1H-κ*N*2-pyrazol-1-yl)-4-oxo-1,4-dihydronaphtalene-1,2-bis(olato)-κ*O*1-κ*O*2)(η^6-p-cymene)osmium(II)]: **2e**

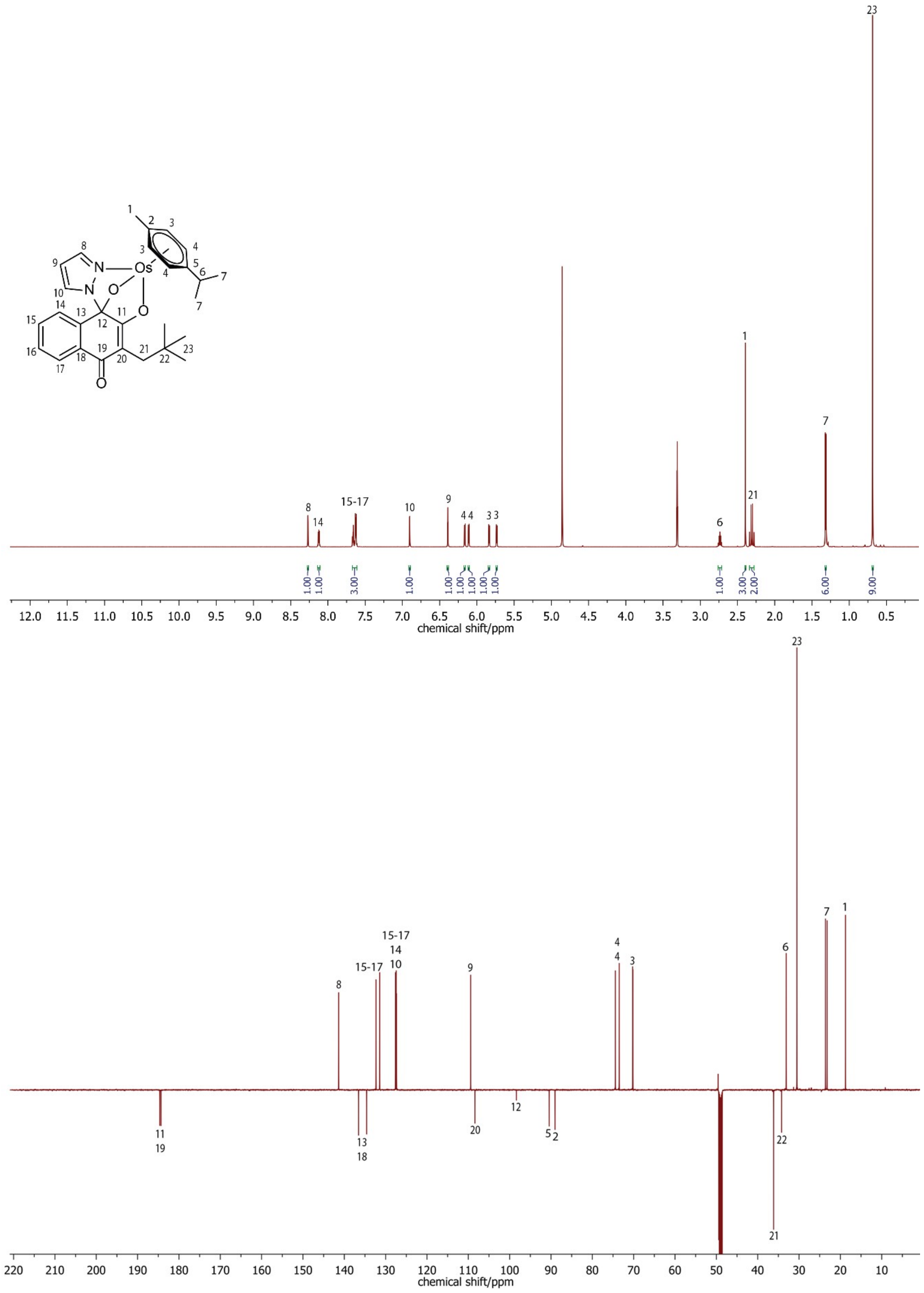

Figure S17: ^{1}H (above) and ^{13}C (below) NMR spectra of complex **2e** in d_4-MeOD.

1.1.18 [(3-(2-Ethylbutyl)-1-(1H-κ*N*2-pyrazol-1-yl)-4-oxo-1,4-dihydronaphtalene-1,2-bis(olato)-κ*O*1-κ*O*2)(η^6-*p*-cymene)osmium(II)]: **2f**

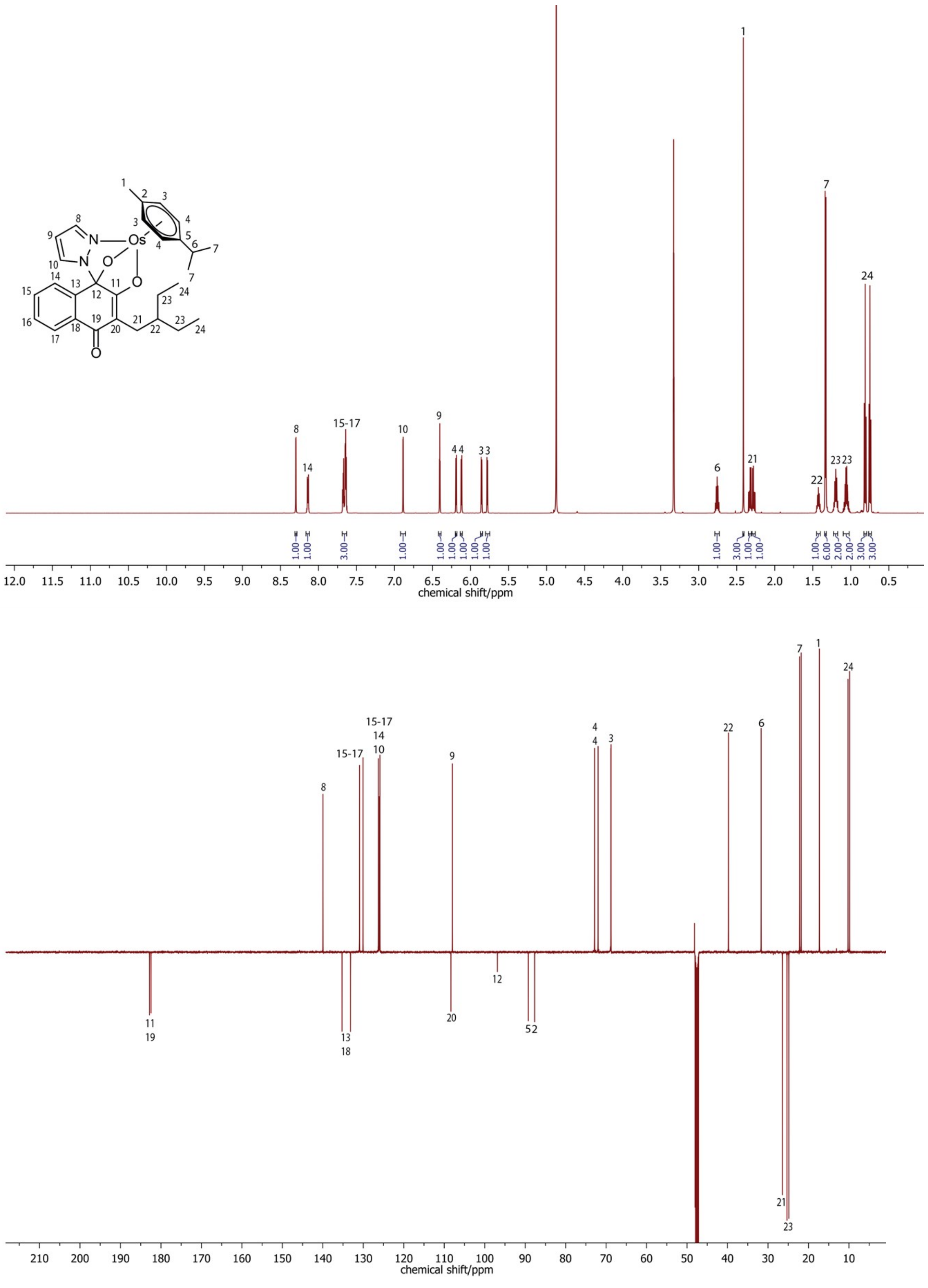

Figure S18: ^{1}H (above) and ^{13}C (below) NMR spectra of complex **2f** in d_4-MeOD.

2 Mass spectra

2.1.1 [(3-Propyl-1-(1H-κ*N*2-pyrazol-1-yl)-4-oxo-1,4-dihydronaphtalene-1,2-bis(olato)-κ*O*1-κ*O*2)(η^6-*p*-cymene)ruthenium(II)]: **1a**

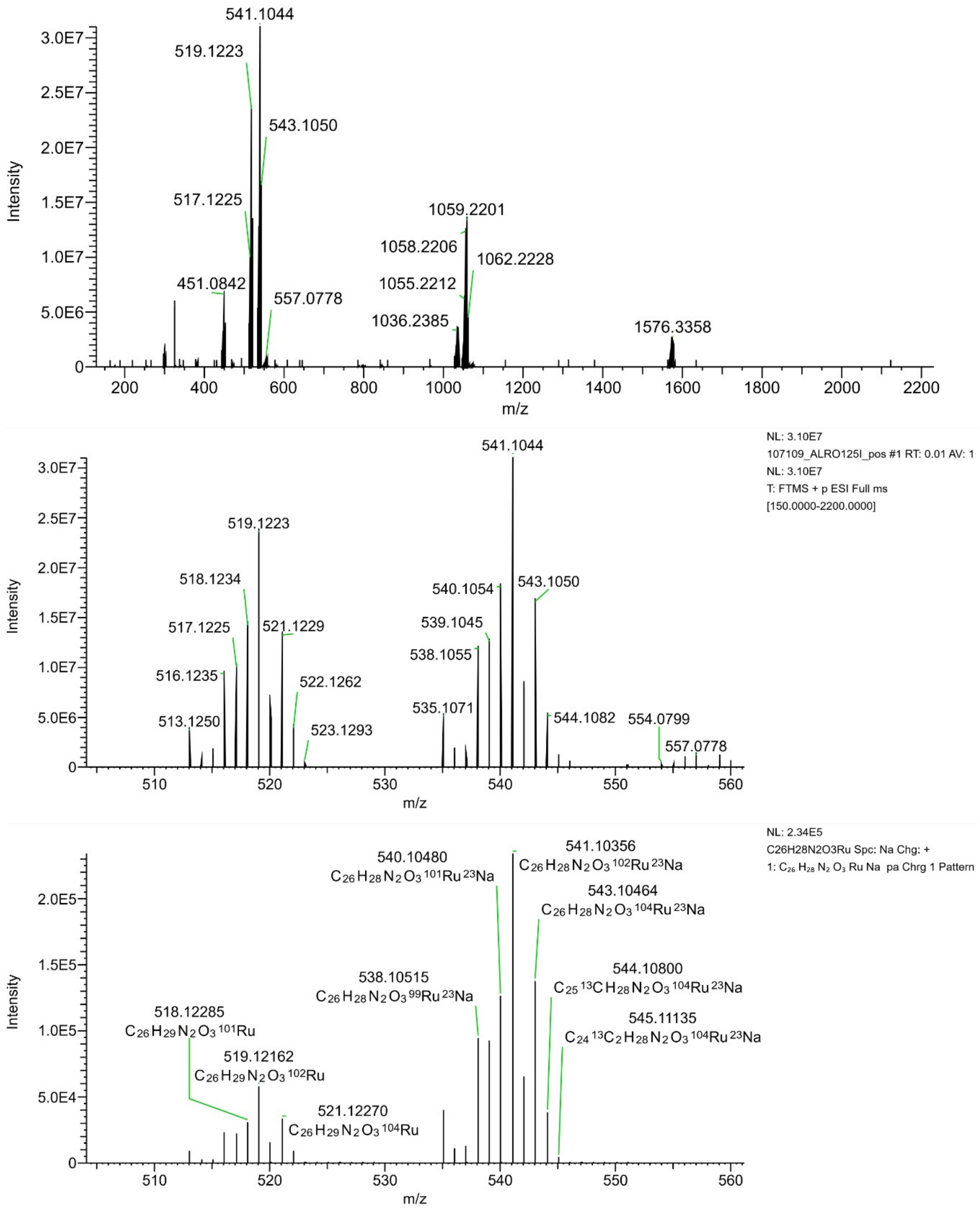

Figure S19: Mass spectrum of complex **1a**.

2.1.2 [(3-Butyl-1-(1H-κ*N*2-pyrazol-1-yl)-4-oxo-1,4-dihydronaphtalene-1,2-bis(olato)-κ*O*1-κ*O*2)(η^6-*p*-cymene)ruthenium(II)]: **1b**

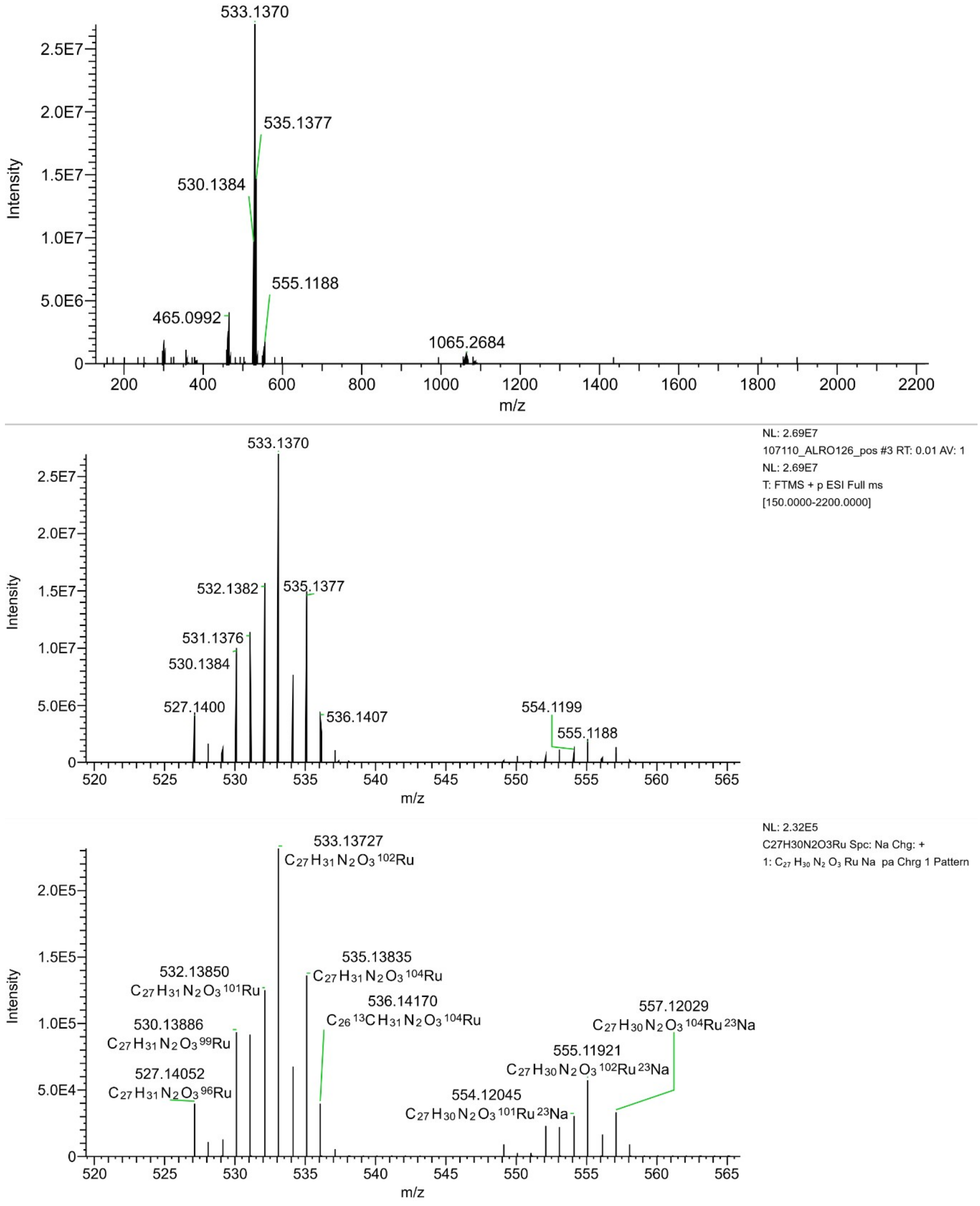

Figure S20: Mass spectrum of complex **1b**.

2.1.3 [(3-Isobutyl-1-(1H-κ*N*2-pyrazol-1-yl)-4-oxo-1,4-dihydronaphtalene-1,2-bis(olato)-κ*O*1-κ*O*2)(η^6-*p*-cymene)ruthenium(II)]: **1c**

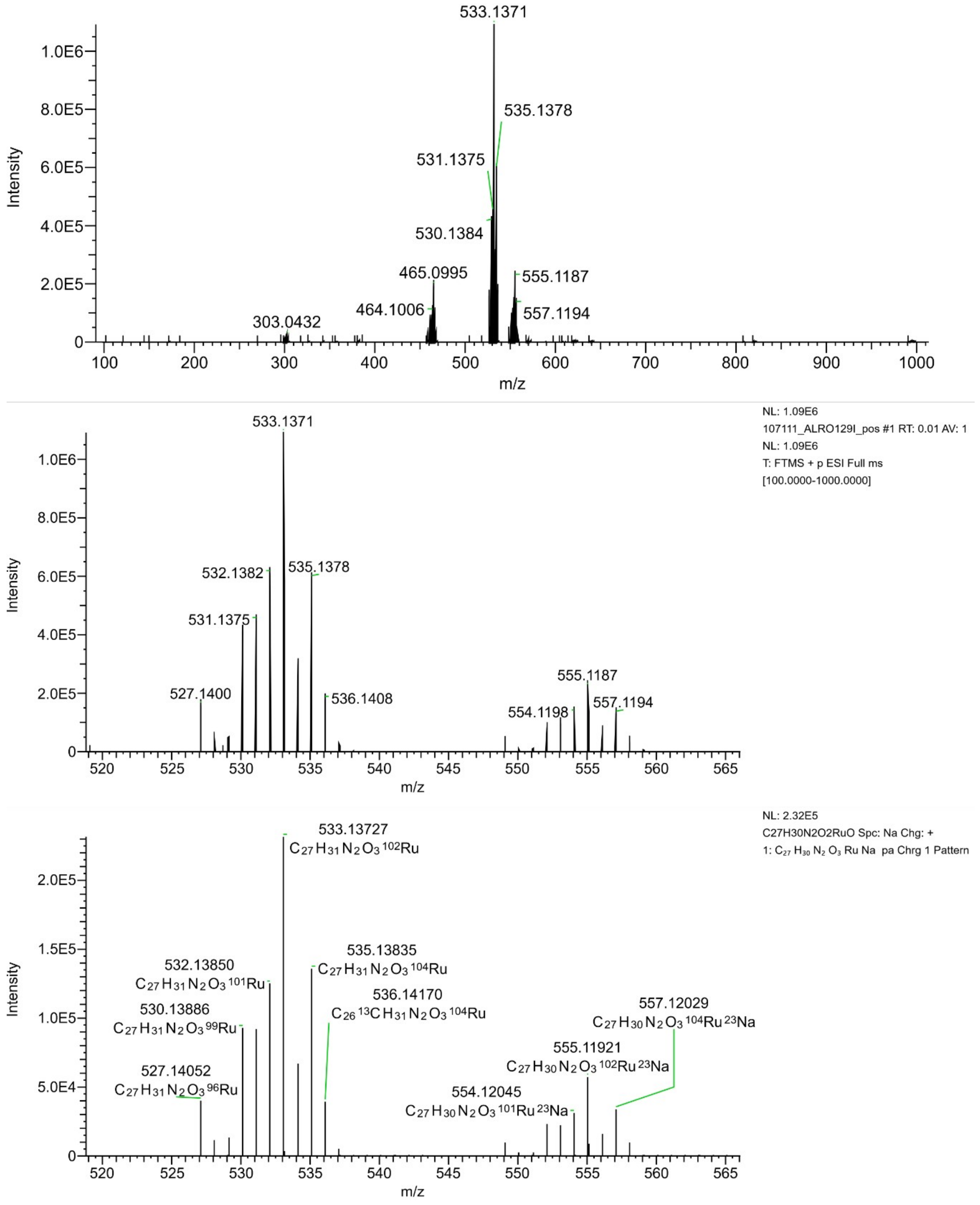

Figure S21: Mass spectrum of complex **1c**.

2.1.4 [(3-(tert-butyl)-1-(1H-κN2-pyrazol-1-yl)-4-oxo-1,4-dihydronaphtalene-1,2-bis(olato)-κO1-κO2)(η6-p-cymene)ruthenium(II)]: **1d**

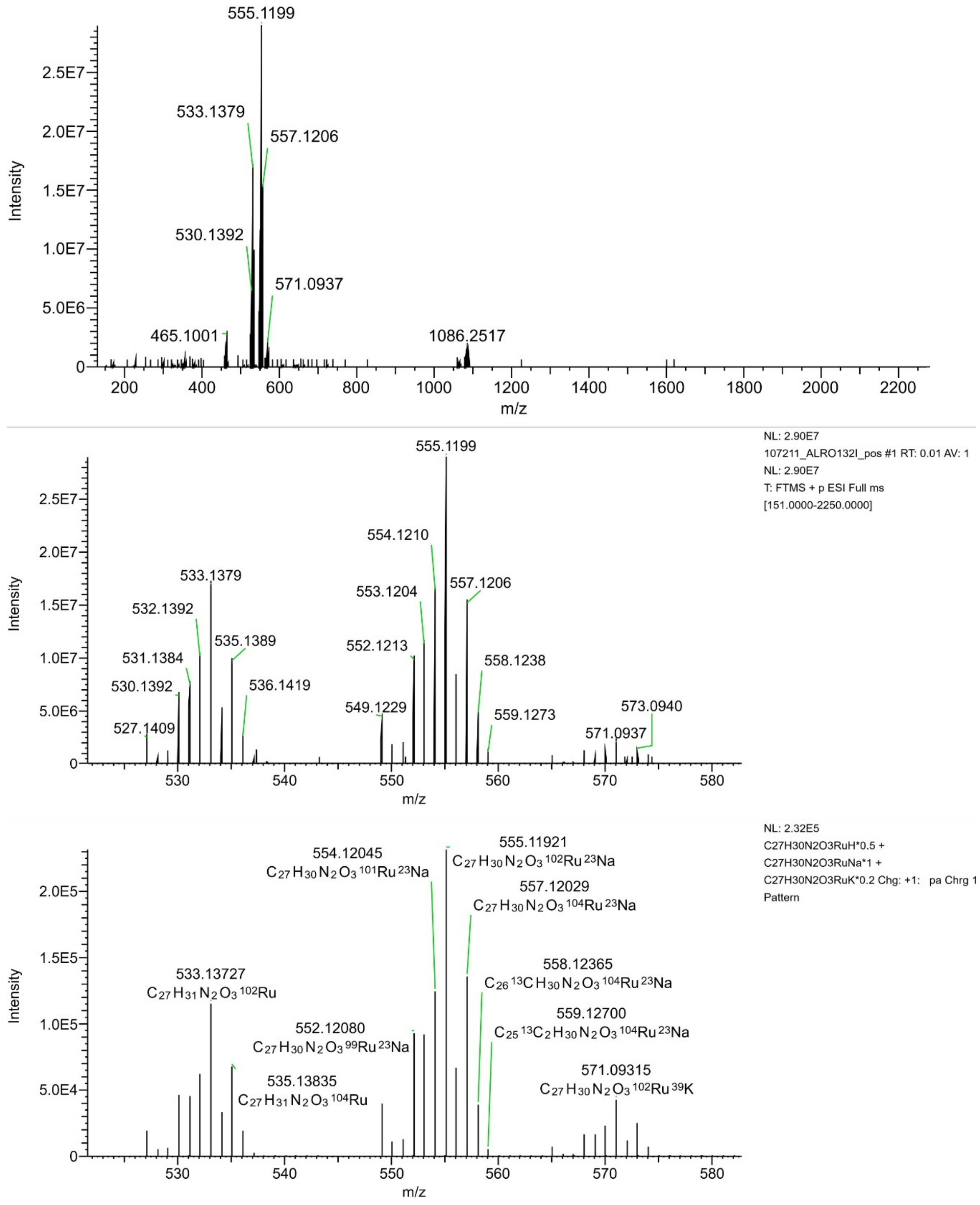

Figure S22: Mass spectrum of complex **1d**.

2.1.5 [(3-Neopentyl-1-(1H-κ*N*2-pyrazol-1-yl)-4-oxo-1,4-dihydronaphtalene-1,2-bis(olato)-κ*O*1-κ*O*2)(η^6-*p*-cymene)ruthenium(II)]: **1e**

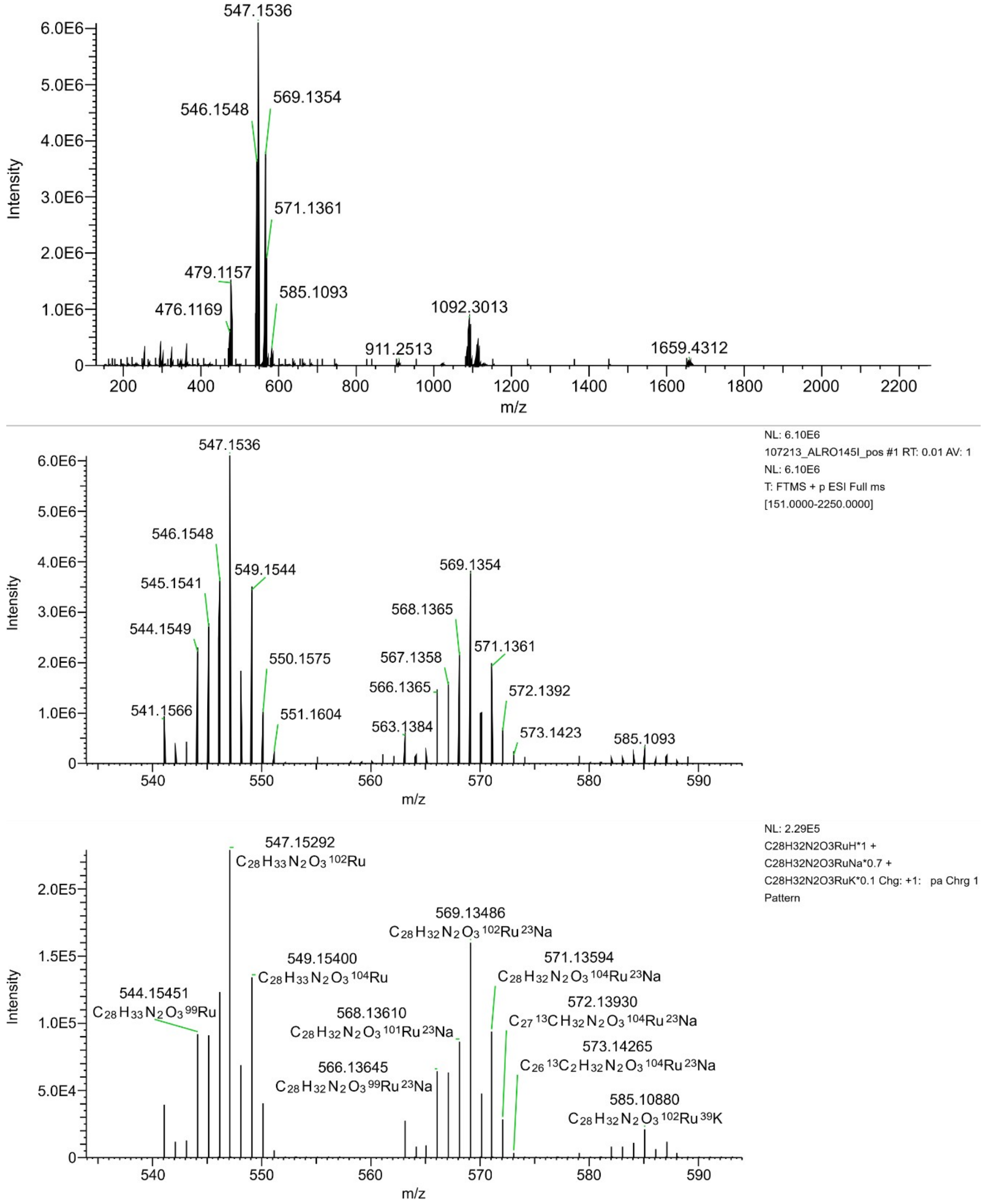

Figure S23: Mass spectrum of complex **1e**.

2.1.6 [(3-(2-Ethylbutyl)-1-(1H-κ*N*2-pyrazol-1-yl)-4-oxo-1,4-dihydronaphtalene-1,2-bis(olato)-κ*O*1-κ*O*2)(η^6-*p*-cymene)ruthenium(II)]: **1f**

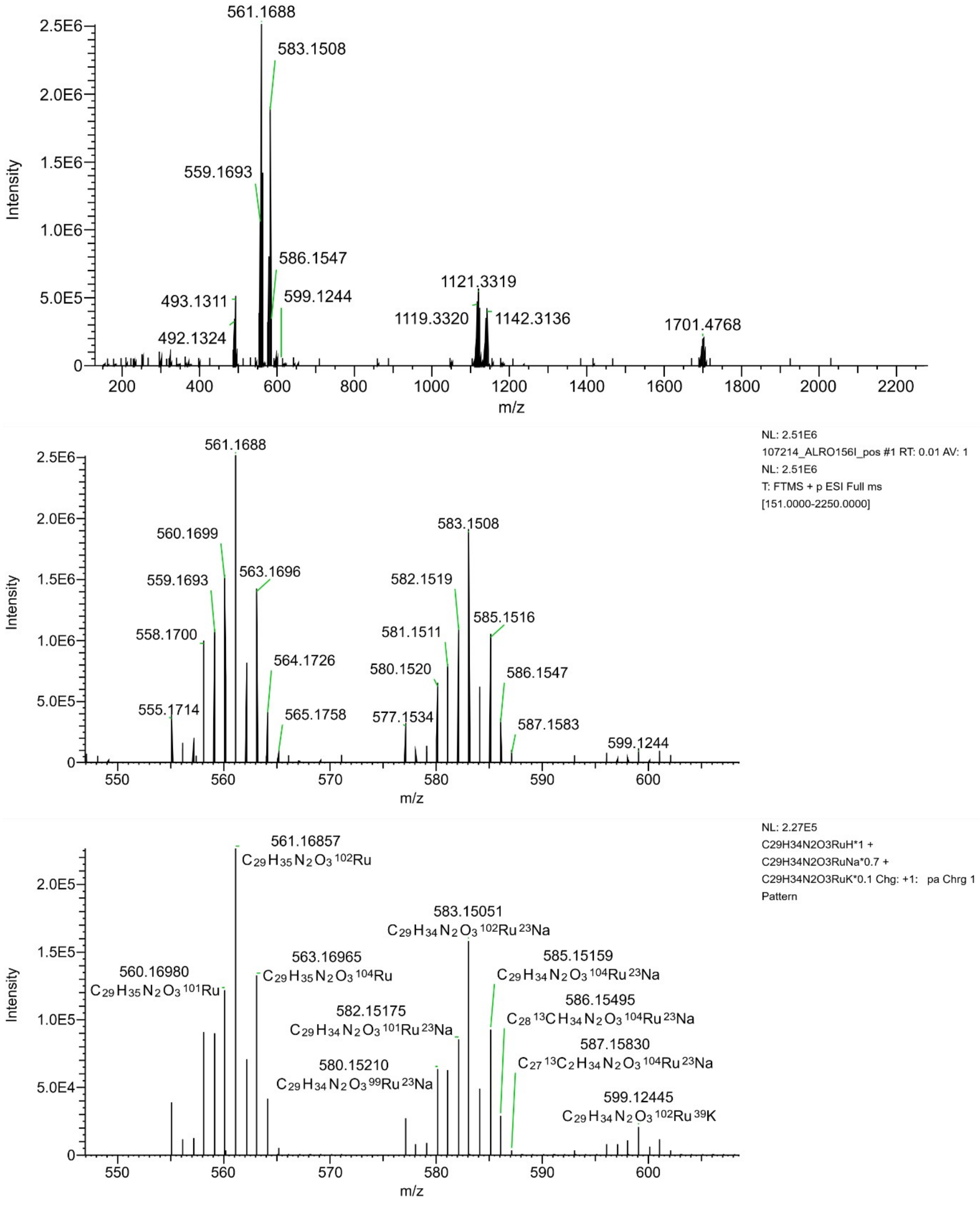

Figure S24: Mass spectrum of complex **1f**.

2.1.7 [(3-Propyl-1-(1H-κ*N*2-pyrazol-1-yl)-4-oxo-1,4-dihydronaphtalene-1,2-bis(olato)-κ*O*1-κ*O*2)(η^6-*p*-cymene)osmium(II)]: **2a**

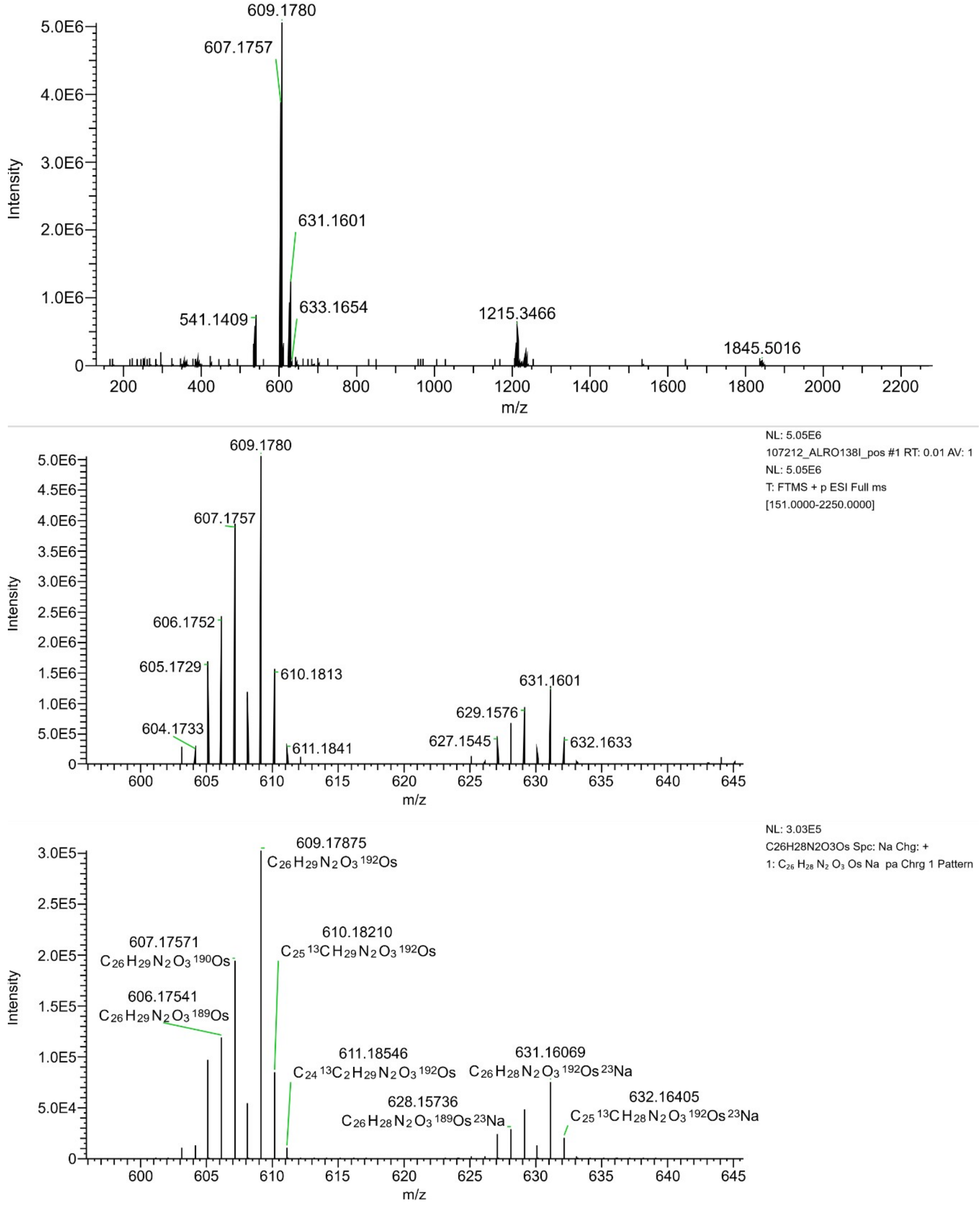

Figure S25: Mass spectrum of complex **2a**.

2.1.8 [(3-Butyl-1-(1H-κ*N*2-pyrazol-1-yl)-4-oxo-1,4-dihydronaphtalene-1,2-bis(olato)-κ*O*1-κ*O*2)(η^6-*p*-cymene)osmium(II)]: **2b**

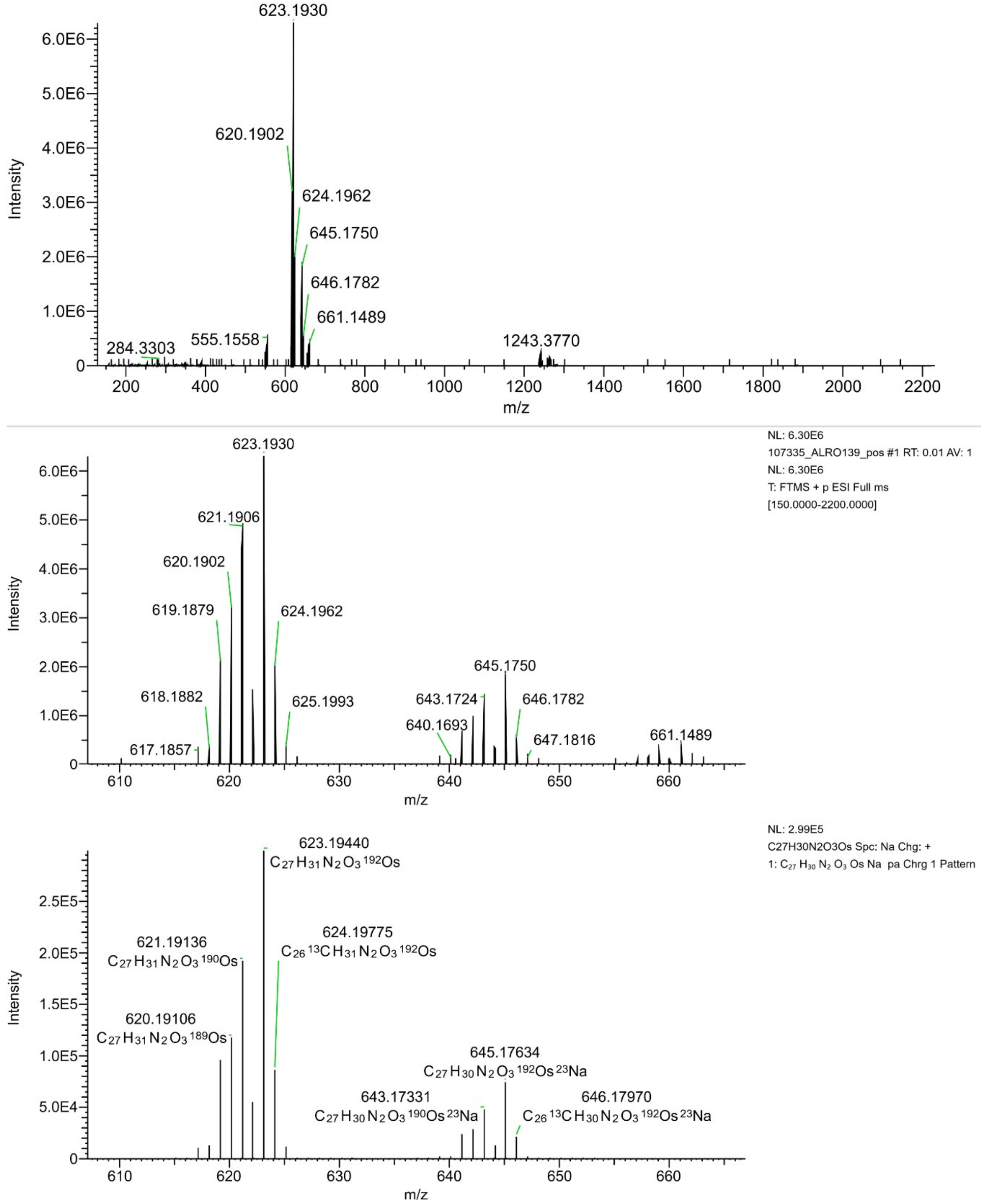

Figure S26: Mass spectrum of complex **2b**.

2.1.9 [(3-Isobutyl-1-(1H-κ*N*2-pyrazol-1-yl)-4-oxo-1,4-dihydronaphtalene-1,2-bis(olato)-κ*O*1-κ*O*2)(η^6-*p*-cymene)osmium(II)]: **2c**

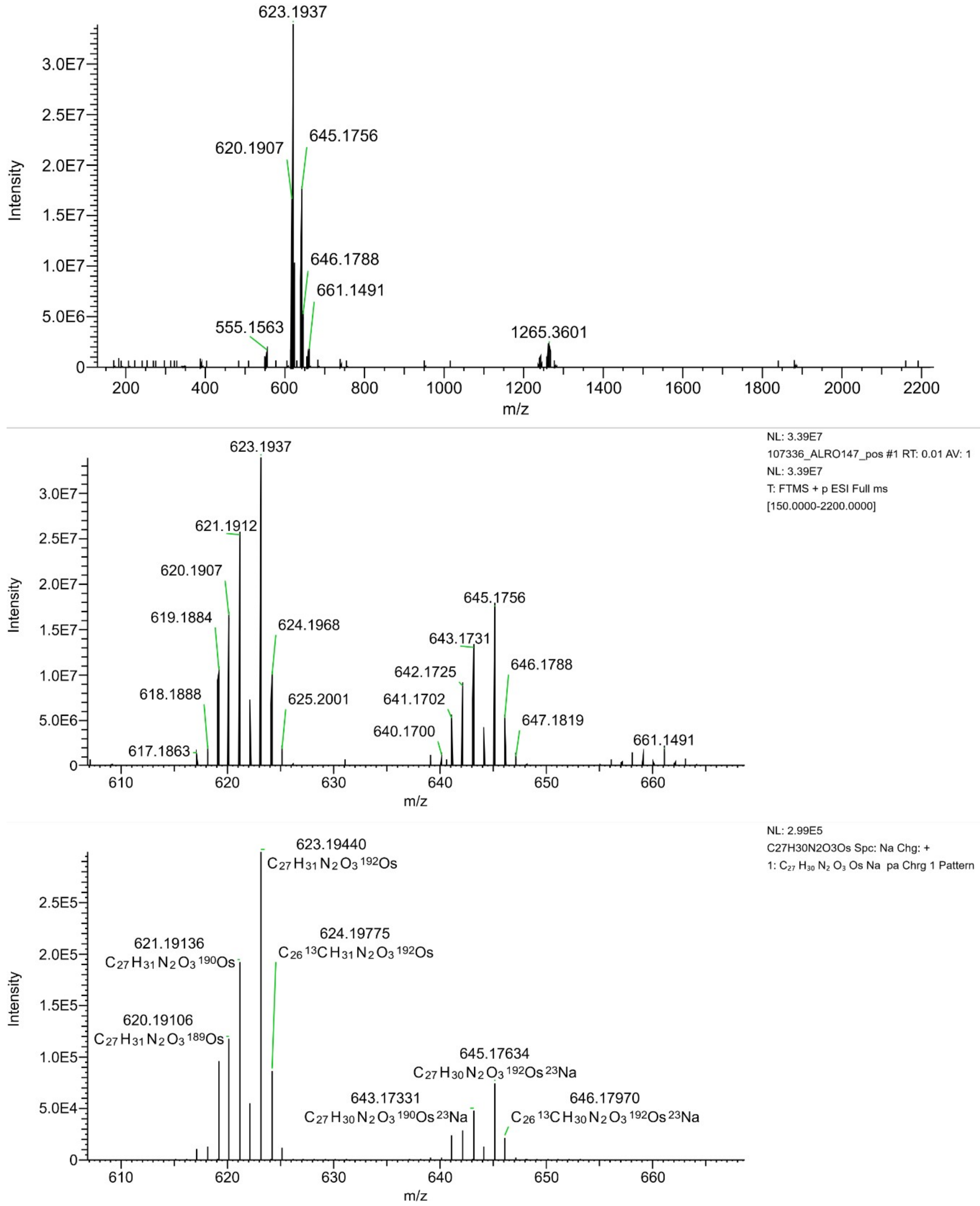

Figure S27: Mass spectrum of complex **2c**.

2.1.10 [(3-(*tert*-butyl)-1-(1H-κ*N*2-pyrazol-1-yl)-4-oxo-1,4-dihydronaphtalene-1,2-bis(olato)-κ*O*1-κ*O*2)(η^6-*p*-cymene)osmium(II)]: **2d**

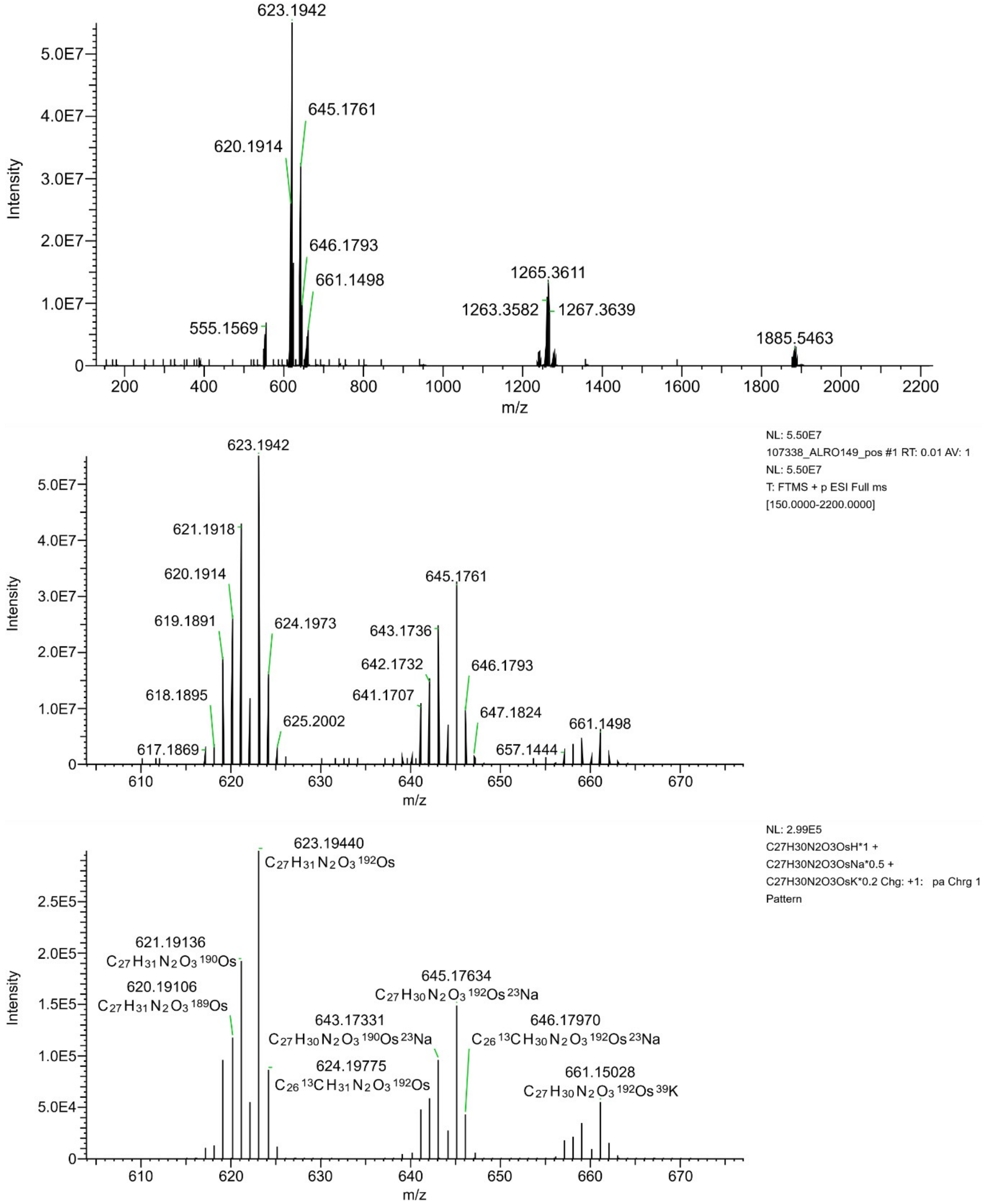

Figure S28: Mass spectrum of complex **2d**.

2.1.11 [(3-Neopentyl-1-(1H-κ*N*2-pyrazol-1-yl)-4-oxo-1,4-dihydronaphtalene-1,2-bis(olato)-κ*O*1-κ*O*2)(η^6-p-cymene)osmium(II)]: **2e**

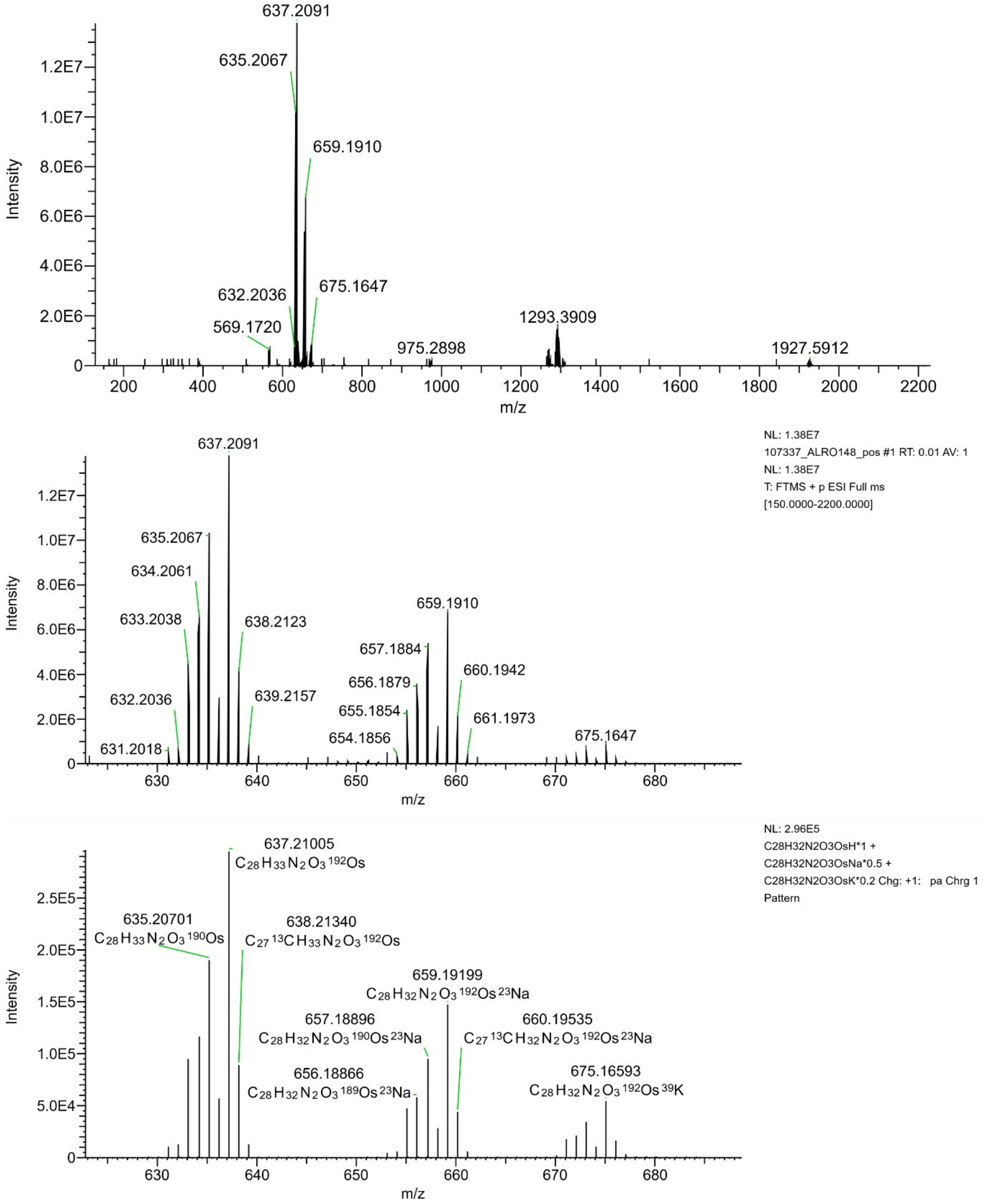

Figure S29: Mass spectrum of complex **2e**.

2.1.12 [(3-(2-Ethylbutyl)-1-(1H-κ*N*2-pyrazol-1-yl)-4-oxo-1,4-dihydronaphtalene-1,2-bis(olato)-κ*O*1-κ*O*2)(η^6-*p*-cymene)osmium(II)]: **2f**

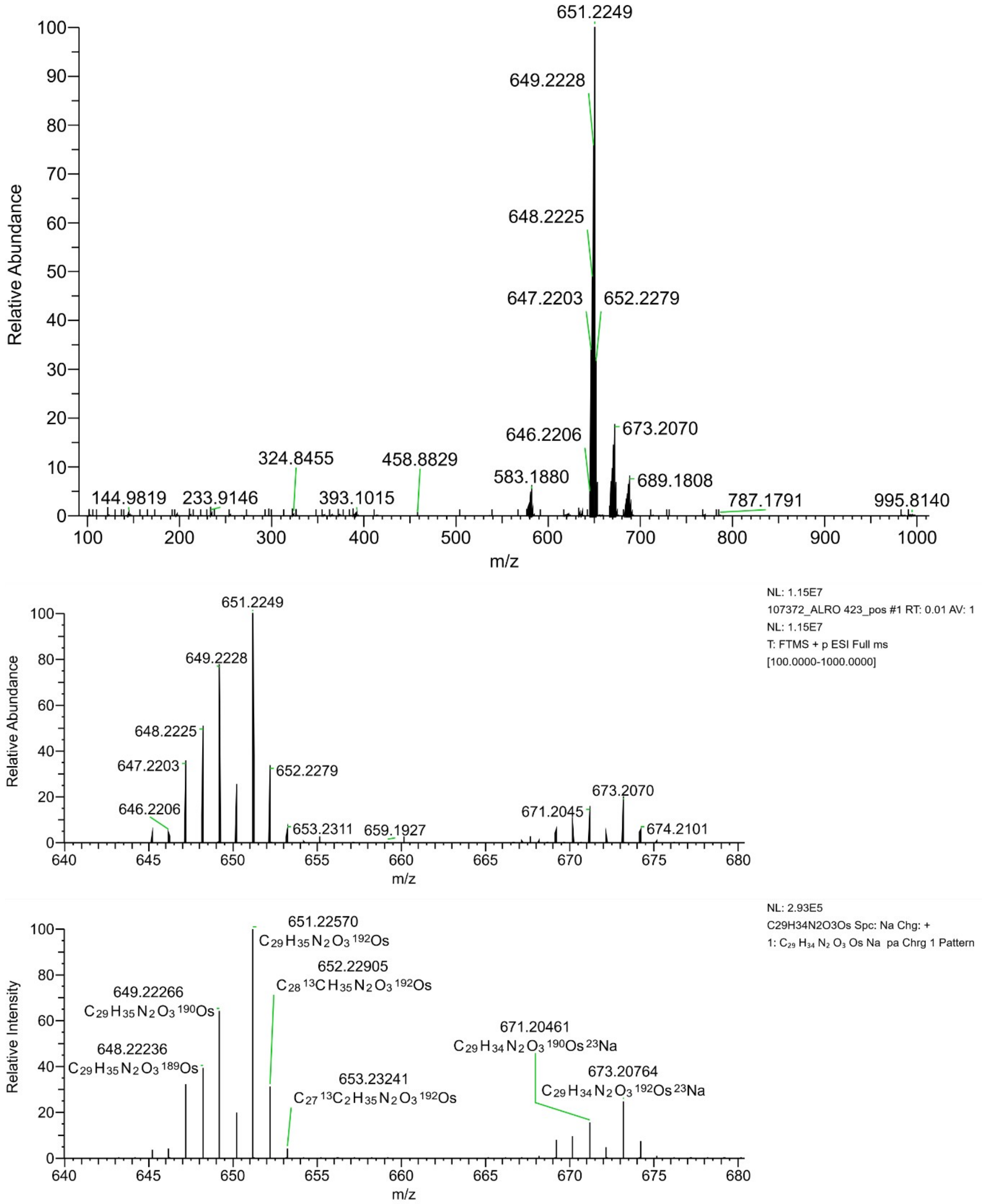

Figure S30: Mass spectrum of complex **2f**.

3 X-ray crystallography data

Single crystals were grown from DCM/toluene/diethyl ether *via* vapor diffusion.

For all samples, compution of molecular graphics, computing of publication material, we used Olex2 1.5-alpha (Dolomanov *et al.*, 2009) and the computing of structure refinement was done *via* SHELXL 2018/3 (Sheldrick, 2015).

3.1.1 [(3-Propyl-1-(1H-κ*N*2-pyrazol-1-yl)-4-oxo-1,4-dihydronaphtalene-1,2-bis(olato)-κ*O*1-κ*O*2)(η^6-*p*-cymene)ruthenium(II)]: **1a (CCDC 2448265)**

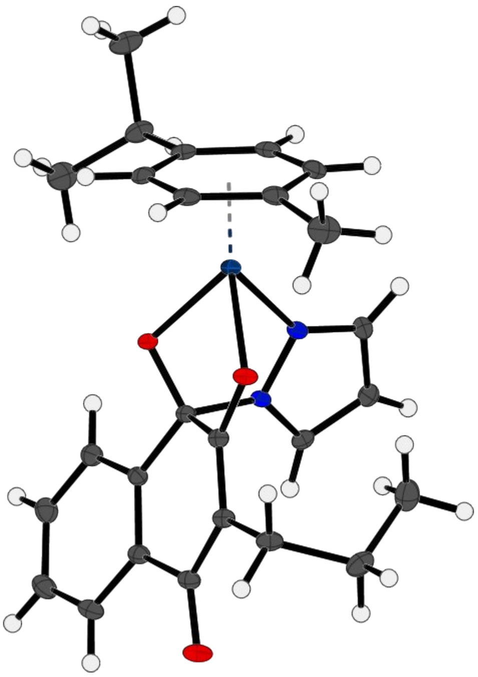

Figure S31: Crystal structure of complex **1a**.

A suitable crystal was selected and analyzed on a Bruker D8 Venture diffractometer. For computing of cell refinement and data collection the APEX4 SUITE was used. This is also valid for all other measurements on BRUKER instruments. The crystal was kept at 100 K during data collection. Using Olex2, the structure was solved with the SHELXS structure solution program using Direct Methods.

Table S1: Sample and crystal data of **1a**.

**Empirical formula**	$C_{26}H_{28}N_2O_3Ru$
**Formula weight**	517.57
**Temperature/K**	100.00
**Crystal system**	triclinic
**Space group**	P-1
**a/Å**	10.1522(3)
**b/Å**	10.4123(4)
**c/Å**	12.5901(4)

**α/°**	102.837(2)
**β/°**	92.546(2)
**γ/°**	118.4480(10)
**Volume/Å^3**	1123.26(7)
**Z**	2
**ρ_{calc}g/cm^3**	1.530
**μ/mm^{-1}**	0.729
**F(000)**	532.0
**Crystal size/mm^3**	0.15 × 0.12 × 0.1
**Radiation**	MoKα (λ = 0.71073)
**2Θ range for data collection/°**	4.63 to 60.19
**Index ranges**	-14 ≤ h ≤ 14, -14 ≤ k ≤ 14, -17 ≤ l ≤ 17
**Reflections collected**	36115
**Independent reflections**	6499 [R_{int} = 0.0298, R_{sigma} = 0.0195]
**Data/restraints/parameters**	6499/0/293
**Goodness-of-fit on F^2**	1.036
**Final R indexes [I>=2σ (I)]**	R_1 = 0.0187, wR_2 = 0.0473
**Final R indexes [all data]**	R_1 = 0.0202, wR_2 = 0.0479
**Largest diff. peak/hole / e Å^{-3}**	0.63/-0.69

3.1.2 [(3-Butyl-1-(1H-κ*N*2-pyrazol-1-yl)-4-oxo-1,4-dihydronaphtalene-1,2-bis(olato)-κ*O*1-κ*O*2)(η^6-*p*-cymene)ruthenium(II)]: **1b (CCDC 2422950)**

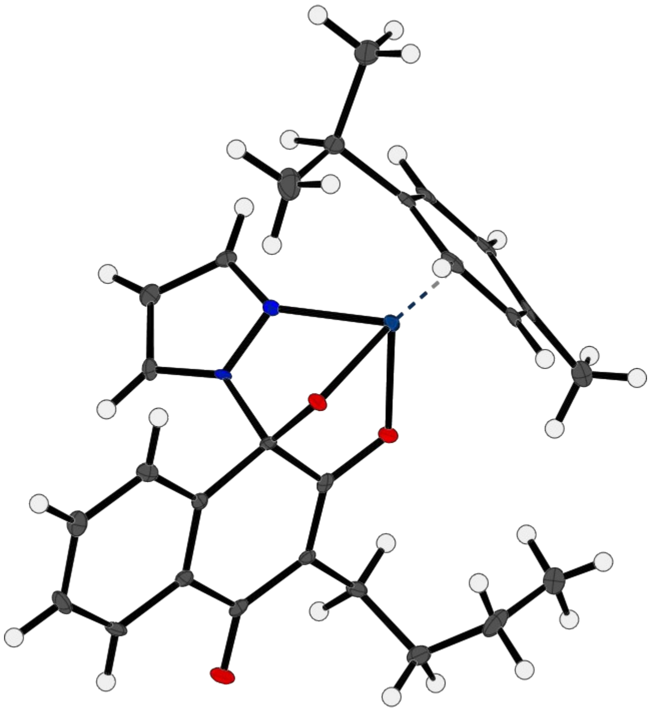

Figure S32: Crystal structure of complex **1b**.

A suitable crystal was selected and analyzed on a STOE STADIVARI diffractometer. For computing of cell refinement, X-Area Recipe 2.0.2 (STOE, 2024) was used. Computing of data collection was conducted *via* X-Area Pilatus3_SV 1.31.194.0 (STOE, 2023). This is also valid for all other measurements on the STOE STADIVARI. The crystal was kept at 100 K during data collection. Using Olex2, the structure was solved with the SHELXS structure solution program using Direct Methods.

Table S2: Sample and crystal data of **1b**.

**Empirical formula**	$C_{27}H_{30}N_2O_3Ru$
**Formula weight**	531.60
**Temperature/K**	100
**Crystal system**	monoclinic
**Space group**	$P2_1/n$
**a/Å**	9.7903(5)
**b/Å**	17.4298(9)
**c/Å**	13.5228(7)
**α/°**	90
**β/°**	94.398(4)

**γ/°**	90
**Volume/Å^3**	2300.8(2)
**Z**	4
**ρ_{calc}g/cm^3**	1.535
**μ/mm^{-1}**	0.714
**F(000)**	1096.0
**Crystal size/mm^3**	0.26 × 0.133 × 0.07
**Radiation**	MoKα (λ = 0.71073)
**2Θ range for data collection/°**	3.82 to 54.966
**Index ranges**	-12 ≤ h ≤ 11, -22 ≤ k ≤ 21, -17 ≤ l ≤ 17
**Reflections collected**	33510
**Independent reflections**	5282 [R_{int} = 0.0402, R_{sigma} = 0.0592]
**Data/restraints/parameters**	5282/6/302
**Goodness-of-fit on F^2**	0.961
**Final R indexes [I>=2σ (I)]**	R_1 = 0.0258, wR_2 = 0.0560
**Final R indexes [all data]**	R_1 = 0.0355, wR_2 = 0.0567

3.1.3 [(3-Isobutyl-1-(1H-κ*N*2-pyrazol-1-yl)-4-oxo-1,4-dihydronaphtalene-1,2-bis(olato)-κ*O*1-κ*O*2)(η^6-*p*-cymene)ruthenium(II)]: **1c (CCDC 2422945)**

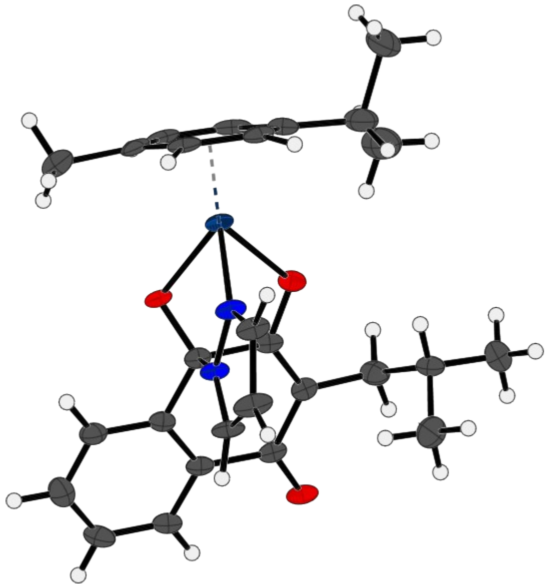

Figure S33: Crystal structure of complex **1c**.

A suitable crystal was selected and analyzed on a STOE STADIVARI diffractometer. The crystal was kept at 100 K during data collection. Using Olex2, the structure was solved with the XT structure solution program using Intrinsic Phasing.

Table S3: Sample and crystal data of **1c**.

**Empirical formula**	$C_{31}H_{40}N_2O_4Ru$
**Formula weight**	605.72
**Temperature/K**	100
**Crystal system**	triclinic
**Space group**	P-1
**a/Å**	9.7041(11)
**b/Å**	12.6241(15)
**c/Å**	14.1057(14)
**α/°**	103.754(8)
**β/°**	100.413(8)
**γ/°**	112.528(8)
**Volume/Å³**	1477.5(3)
**Z**	2
**ρ_{calc}g/cm³**	1.361
**μ/mm⁻¹**	0.567
**F(000)**	632.0
**Crystal size/mm³**	0.13 × 0.073 × 0.03
**Radiation**	MoKα (λ = 0.71073)
**2Θ range for data collection/°**	4.768 to 57.68

**Index ranges**	$-12 \leq h \leq 12$, $-16 \leq k \leq 16$, $-18 \leq l \leq 19$
**Reflections collected**	30235
**Independent reflections**	7146 [R_{int} = 0.0673, R_{sigma} = 0.1639]
**Data/restraints/parameters**	7146/395/375
**Goodness-of-fit on F^2**	0.911
**Final R indexes [I>=2σ (I)]**	R_1 = 0.0433, wR_2 = 0.0808
**Final R indexes [all data]**	R_1 = 0.0832, wR_2 = 0.0852
**Largest diff. peak/hole / e Å^{-3}**	0.98/-1.38

3.1.4 [(3-(tert-butyl)-1-(1H-κN2-pyrazol-1-yl)-4-oxo-1,4-dihydronaphtalene-1,2-bis(olato)-κO1-κO2)(η6-p-cymene)ruthenium(II)]: **1d (CCDC 2448267)**

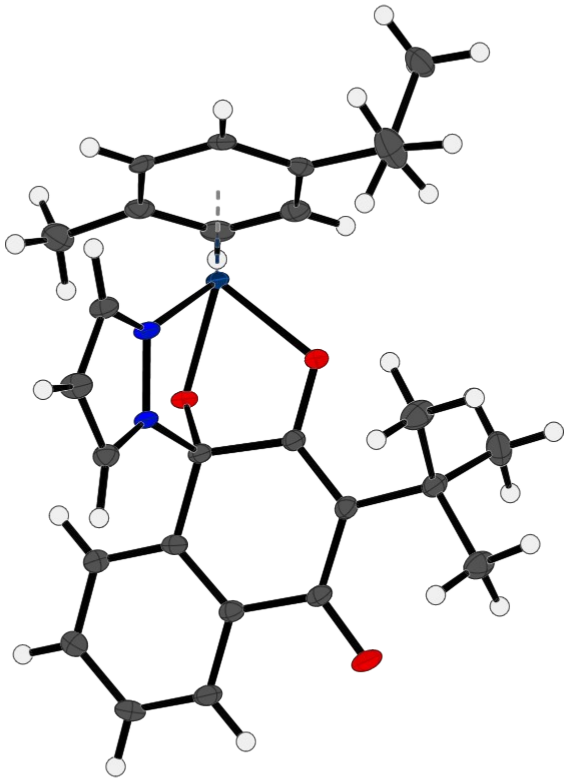

Figure S34: Crystal structure of complex **1d**.

A suitable crystal was selected and analyzed on a Bruker D8 Venture diffractometer. The crystal was kept at 100.00 K during data collection. Using Olex2, the structure was solved with the XS structure solution program using Direct Methods.

Table S4: Sample and crystal data of **1d**.

**Empirical formula**	$C_{27}H_{30}N_2O_3Ru$
**Formula weight**	531.60
**Temperature/K**	100.00
**Crystal system**	triclinic
**Space group**	P-1
**a/Å**	9.7915(18)
**b/Å**	9.8922(18)
**c/Å**	13.6713(16)
**α/°**	101.738(8)
**β/°**	108.241(8)
**γ/°**	96.812(9)

**Volume/Å^3**	1207.5(3)
**Z**	2
**ρ_{calc}g/cm^3**	1.462
**μ/mm^{-1}**	0.680
**F(000)**	548.0
**Crystal size/mm^3**	0.2 × 0.15 × 0.05
**Radiation**	MoKα (λ = 0.71073)
**2Θ range for data collection/°**	4.288 to 56.562
**Index ranges**	-13 ≤ h ≤ 13, -13 ≤ k ≤ 13, -18 ≤ l ≤ 18
**Reflections collected**	54782
**Independent reflections**	5994 [R_{int} = 0.0314, R_{sigma} = 0.0160]
**Data/restraints/parameters**	5994/0/304
**Goodness-of-fit on F^2**	1.061
**Final R indexes [I>=2σ (I)]**	R_1 = 0.0206, wR_2 = 0.0504
**Final R indexes [all data]**	R_1 = 0.0222, wR_2 = 0.0509
**Largest diff. peak/hole / e Å^{-3}**	1.34/-0.75

3.1.5 [(3-Neopentyl-1-(1H-κ*N*2-pyrazol-1-yl)-4-oxo-1,4-dihydronaphtalene-1,2-bis(olato)-κ*O*1-κ*O*2)(η^6-*p*-cymene)ruthenium(II)]: **1e (CCDC 2469756)**

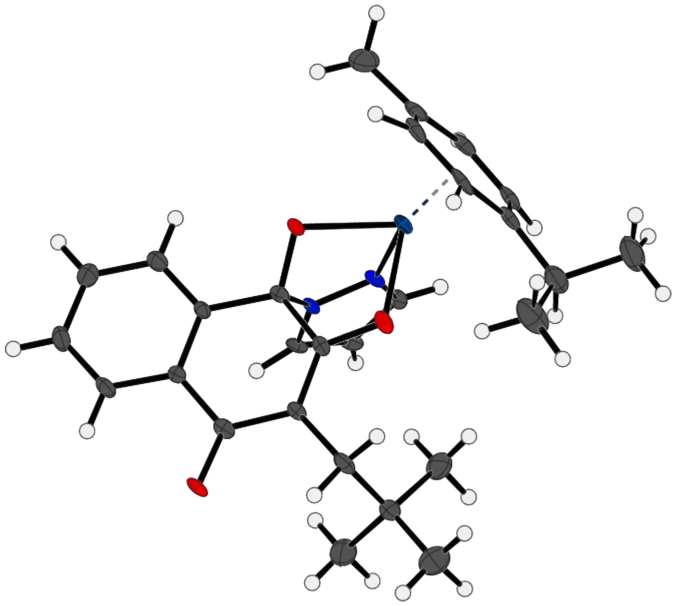

Figure S35: Crystal structure of complex **1e**.

A suitable crystal was selected and analyzed on a STOE STADIVARI diffractometer. The crystal was kept at 100.15 K during data collection. Using Olex2, the structure was solved with the XS structure solution program using Direct Methods.

Table S5:Sample and crystal data of **1e**.

Empirical formula	$C_{32}H_{42}N_2O_4Ru$
Formula weight	619.74
Temperature/K	100.15
Crystal system	triclinic
Space group	P-1
a/Å	9.6961(5)
b/Å	12.7169(8)
c/Å	14.3705(9)
α/°	103.068(5)
β/°	102.083(5)
γ/°	111.797(4)
Volume/Å^3	1516.05(17)
Z	2
ρ_{calc}g/cm^3	1.358
μ/mm^{-1}	0.555
F(000)	648.0
Crystal size/mm^3	0.2 × 0.093 × 0.02
Radiation	MoKα (λ = 0.71073)
2Θ range for data collection/°	5.688 to 57.222

Index ranges	$-10 \leq h \leq 12$, $-16 \leq k \leq 16$, $-19 \leq l \leq 18$
Reflections collected	35415
Independent reflections	7285 [R_{int} = 0.0694, R_{sigma} = 0.1394]
Data/restraints/parameters	7285/154/360
Goodness-of-fit on F^2	0.951
Final R indexes [I>=2σ (I)]	R_1 = 0.0423, wR_2 = 0.0762
Final R indexes [all data]	R_1 = 0.0831, wR_2 = 0.0823
Largest diff. peak/hole / e Å^{-3}	1.19/-2.33

3.1.6 [(3-(2-Ethylbutyl)-1-(1H-κ*N*2-pyrazol-1-yl)-4-oxo-1,4-dihydronaphtalene-1,2-bis(olato)-κ*O*1-κ*O*2)(η^6-*p*-cymene)ruthenium(II)]: **1f (CCDC 2448913)**

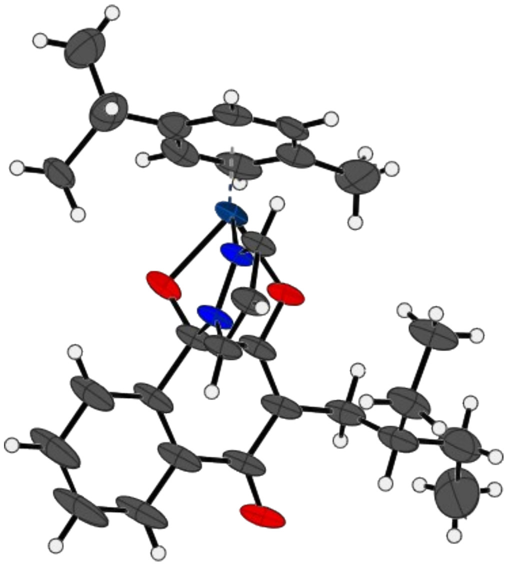

Figure S36: Crystal structure of complex **1f**.

A suitable crystal was selected and analyzed on a STOE STADIVARI diffractometer. The crystal was kept at 100 K during data collection. Using Olex2, the structure was solved with the SHELXT structure solution program using Intrinsic Phasing.

Table S6: Sample and crystal data of **1f**.

**Empirical formula**	$C_{29}H_{34}N_2O_3Ru$
**Formula weight**	559.65
**Temperature/K**	100
**Crystal system**	triclinic
**Space group**	P-1
**a/Å**	12.4670(4)
**b/Å**	14.7959(5)
**c/Å**	15.8824(5)
**α/°**	70.962(2)
**β/°**	81.738(3)
**γ/°**	70.666(2)
**Volume/Å^3**	2611.15(15)
**Z**	4
**ρ_{calc}g/cm^3**	1.424
**μ/mm^{-1}**	0.633
**F(000)**	1160.0
**Crystal size/mm^3**	0.22 × 0.1 × 0.03
**Radiation**	Mo Kα (λ = 0.71073)
**2Θ range for data collection/°**	3.056 to 60.066
**Index ranges**	$-17 \leq h \leq 17, -20 \leq k \leq 20, -22 \leq l \leq 22$

**Reflections collected**	77228
**Independent reflections**	15110 [R_{int} = 0.0383, R_{sigma} = 0.0673]
**Data/restraints/parameters**	15110/288/746
**Goodness-of-fit on F^2**	0.919
**Final R indexes [I>=2σ (I)]**	R_1 = 0.0397, wR_2 = 0.0937
**Final R indexes [all data]**	R_1 = 0.0830, wR_2 = 0.1017
**Largest diff. peak/hole / e $Å^{-3}$**	1.55/-1.08

3.1.7 [(3-Propyl-1-(1H-κ*N*2-pyrazol-1-yl)-4-oxo-1,4-dihydronaphtalene-1,2-bis(olato)-κ*O*1-κ*O*2)(η^6-*p*-cymene)osmium(II)]: **2a (CCDC 2469757)**

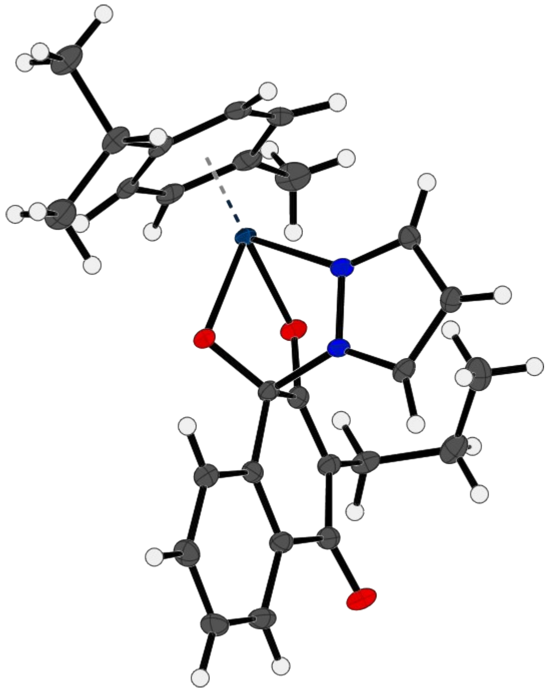

Figure S37: Crystal structure of complex **2a**.

A suitable crystal was selected and analyzed on a STOE STADIVARI diffractometer. The crystal was kept at 100 K during data collection. Using Olex2, the structure was solved with the SHELXT structure solution program using Intrinsic Phasing.

Table 7: Sample and crystal data of **2a**.

Empirical formula	$C_{26}H_{28}N_2O_3Os$
Formula weight	606.70
Temperature/K	100
Crystal system	triclinic
Space group	P-1
a/Å	10.174(2)
b/Å	10.412(2)
c/Å	12.597(3)
α/°	102.88(3)
β/°	92.89(3)
γ/°	118.32(3)
Volume/Å^3	1126.1(5)
Z	2
ρ_{calc}g/cm^3	1.789
μ/mm^{-1}	10.943
F(000)	596.0
Crystal size/mm^3	0.12 × 0.083 × 0.05

Radiation	Cu Kα (λ = 1.54178)
2Θ range for data collection/°	7.32 to 142.956
Index ranges	-12 ≤ h ≤ 10, -12 ≤ k ≤ 10, -9 ≤ l ≤ 15
Reflections collected	21447
Independent reflections	4128 [R_{int} = 0.0122, R_{sigma} = 0.0080]
Data/restraints/parameters	4128/0/293
Goodness-of-fit on F^2	1.097
Final R indexes [I>=2σ (I)]	R_1 = 0.0167, wR_2 = 0.0412
Final R indexes [all data]	R_1 = 0.0171, wR_2 = 0.0414
Largest diff. peak/hole / e $Å^{-3}$	0.44/-1.13

3.1.8 [(3-Butyl-1-(1H-κ*N*2-pyrazol-1-yl)-4-oxo-1,4-dihydronaphtalene-1,2-bis(olato)-κ*O*1-κ*O*2)(η^6-*p*-cymene)osmium(II)]: **2b (CCDC 2448268)**

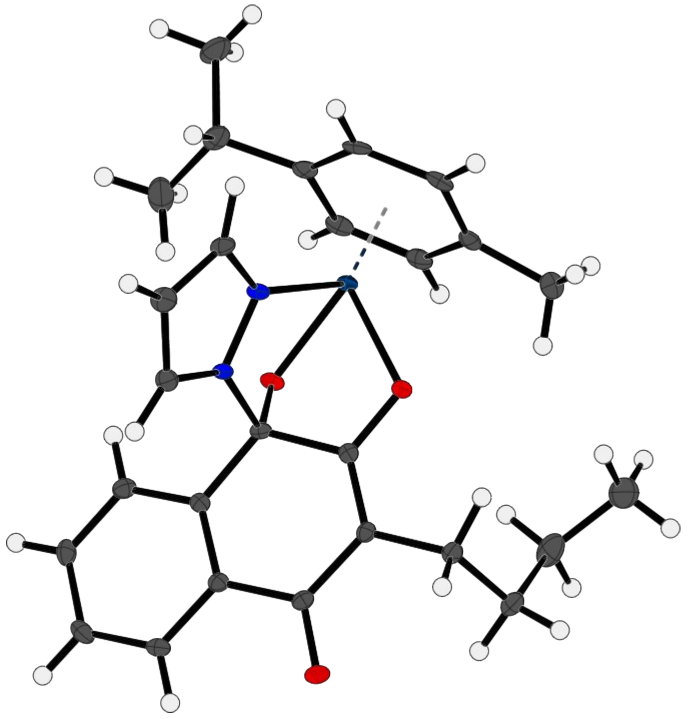

Figure S38: Crystal structure of complex **2b**.

A suitable crystal was selected and analyzed on a Bruker D8 Venture diffractometer. The crystal was kept at 100.00 K during data collection. Using Olex2, the structure was solved with the SHELXT structure solution program using Intrinsic Phasing.

Table S8: Sample and crystal data of **2b**.

Empirical formula	$C_{27}H_{30}N_2O_3Os$
Formula weight	620.73
Temperature/K	100.00
Crystal system	monoclinic
Space group	$P2_1/n$
a/Å	9.8350(4)
b/Å	17.5139(4)
c/Å	13.5380(3)
α/°	90
β/°	94.237(2)
γ/°	90
Volume/Å^3	2325.54(12)

Z	4
ρ_{calc}g/cm^3	1.773
μ/mm^{-1}	5.515
F(000)	1224.0
Crystal size/mm^3	0.18 × 0.1 × 0.08
Radiation	MoKα (λ = 0.71073)
2Θ range for data collection/°	3.81 to 52.744
Index ranges	-12 ≤ h ≤ 12, -21 ≤ k ≤ 21, -16 ≤ l ≤ 16
Reflections collected	101905
Independent reflections	4745 [R_{int} = 0.0371, R_{sigma} = 0.0116]
Data/restraints/parameters	4745/0/302
Goodness-of-fit on F^2	1.075
Final R indexes [I>=2σ (I)]	R_1 = 0.0134, wR_2 = 0.0318
Final R indexes [all data]	R_1 = 0.0152, wR_2 = 0.0323
Largest diff. peak/hole / e Å^{-3}	1.88/-0.89

3.1.9 [(3-Isobutyl-1-(1H-κ*N*2-pyrazol-1-yl)-4-oxo-1,4-dihydronaphtalene-1,2-bis(olato)-κ*O*1-κ*O*2)(η^6-*p*-cymene)osmium(II)]: **2c (CCDC 2422948)**

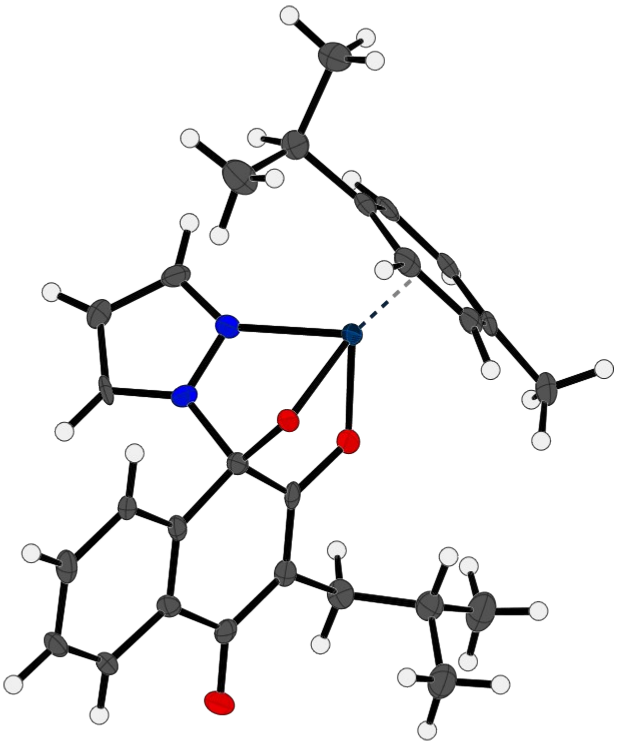

Figure S39: Crystal structure of complex **2c**.

A suitable crystal was selected and analyzed on a STOE STADIVARI diffractometer. The crystal was kept at 100 K during data collection. Using Olex2, the structure was solved with the SHELXT structure solution program using Intrinsic Phasing.

Table S9: Sample and crystal data of **2c**.

Empirical formula	$C_{27}H_{30}N_2O_3Os$
Formula weight	620.73
Temperature/K	100
Crystal system	monoclinic
Space group	$P2_1/n$
a/Å	10.1908(5)
b/Å	17.8342(8)
c/Å	12.9983(8)
α/°	90

β/°	95.158(5)
γ/°	90
Volume/Å^3	2352.8(2)
Z	4
ρ_{calc}g/cm^3	1.752
μ/mm^{-1}	5.452
F(000)	1224.0
Crystal size/mm^3	0.13 × 0.05 × 0.04
Radiation	Mo Kα (λ = 0.71073)
2Θ range for data collection/°	3.888 to 51.35
Index ranges	$-11 \leq h \leq 12$, $-21 \leq k \leq 21$, $-15 \leq l \leq 15$
Reflections collected	27723
Independent reflections	4286 [R_{int} = 0.0454, R_{sigma} = 0.0426]
Data/restraints/parameters	4286/0/304
Goodness-of-fit on F^2	1.011
Final R indexes [I>=2σ (I)]	R_1 = 0.0380, wR_2 = 0.0922
Final R indexes [all data]	R_1 = 0.0551, wR_2 = 0.0982
Largest diff. peak/hole / e Å^{-3}	1.75/-2.47

3.1.10 [(3-(*tert*-butyl)-1-(1H-κ*N*2-pyrazol-1-yl)-4-oxo-1,4-dihydronaphtalene-1,2-bis(olato)-κ*O*1-κ*O*2)(η^6-*p*-cymene)osmium(II)]: **2d (CCDC 2422949)**

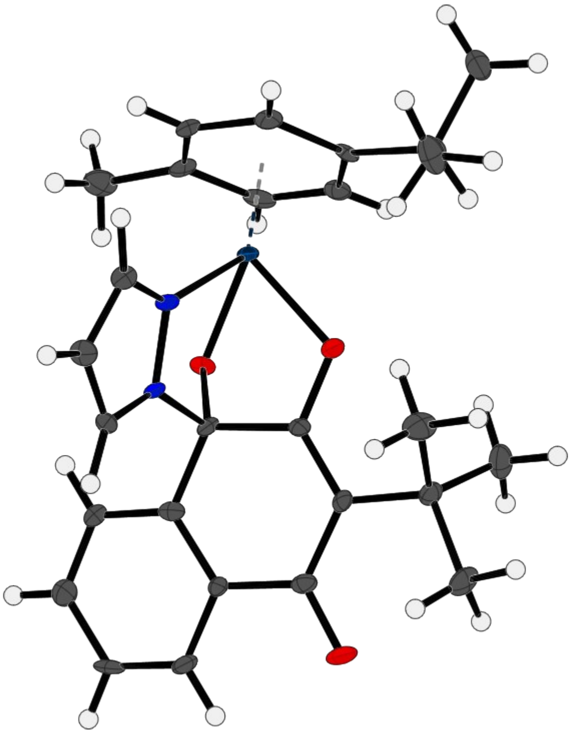

Figure S40: Crystal structure of complex **2d**.

A suitable crystal was selected and analyzed on a STOE STADIVARI diffractometer. The crystal was kept at 100 K during data collection. Using Olex2, the structure was solved with the SHELXT structure solution program using Intrinsic.

Table S10: Sample and crystal data of **2d**.

Empirical formula	$C_{27}H_{30}N_2O_3Os$
Formula weight	620.73
Temperature/K	100
Crystal system	triclinic
Space group	P-1
a/Å	9.7513(4)
b/Å	9.8612(4)
c/Å	13.5758(6)
α/°	101.981(3)
β/°	108.052(3)
γ/°	96.820(3)

Volume/Å^3	1190.39(9)
Z	2
ρ_{calc}g/cm^3	1.732
μ/mm^{-1}	5.387
F(000)	612.0
Crystal size/mm^3	0.125 × 0.065 × 0.02
Radiation	Mo Kα (λ = 0.71073)
2Θ range for data collection/°	6.288 to 51.364
Index ranges	-11 ≤ h ≤ 6, -11 ≤ k ≤ 11, -16 ≤ l ≤ 16
Reflections collected	18197
Independent reflections	4318 [R_{int} = 0.0494, R_{sigma} = 0.0585]
Data/restraints/parameters	4318/0/304
Goodness-of-fit on F^2	0.997
Final R indexes [I>=2σ (I)]	R_1 = 0.0340, wR_2 = 0.0772
Final R indexes [all data]	R_1 = 0.0449, wR_2 = 0.0796
Largest diff. peak/hole / e Å^{-3}	2.38/-1.49

3.1.11 [(3-Neopentyl-1-(1H-κ*N*2-pyrazol-1-yl)-4-oxo-1,4-dihydronaphtalene-1,2-bis(olato)-κ*O*1-κ*O*2)(η^6-p-cymene)osmium(II)]: **2e (CCDC 2422947)**

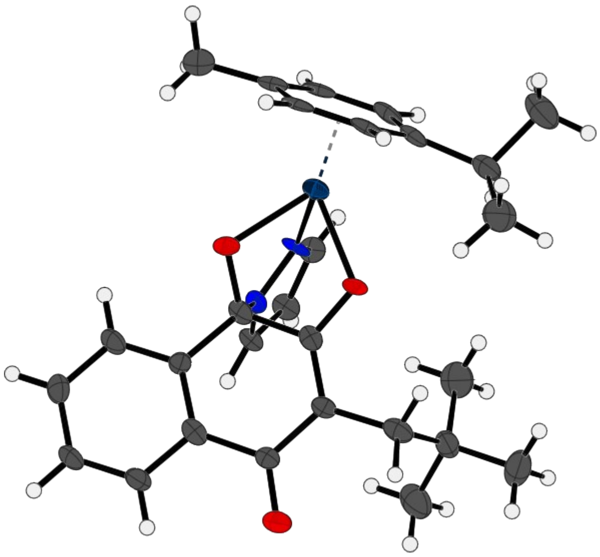

Figure S41: Crystal structure of complex **2e**.

A suitable crystal was selected and analyzed on a STOE STADIVARI diffractometer. The crystal was kept at 100 K during data collection. Using Olex2, the structure was solved with the SHELXT structure solution program using Intrinsic Phasing.

Table S11: Sample and crystal data of **2e**.

Empirical formula	$C_{32}H_{42}N_2O_4Os$
Formula weight	708.87
Temperature/K	100
Crystal system	triclinic
Space group	P-1
a/Å	9.7072(6)
b/Å	12.7353(8)
c/Å	14.3871(8)
α/°	102.980(5)
β/°	102.104(5)
γ/°	111.815(5)
Volume/Å^3	1522.21(17)
Z	2

ρ_{calc}g/cm^3	1.547
μ/mm^{-1}	4.226
F(000)	712.0
Crystal size/mm^3	0.063 × 0.059 × 0.01
Radiation	Mo Kα (λ = 0.71073)
2Θ range for data collection/°	4.77 to 52.944
Index ranges	-12 ≤ h ≤ 11, -15 ≤ k ≤ 14, -17 ≤ l ≤ 17
Reflections collected	26452
Independent reflections	5807 [R_{int} = 0.0713, R_{sigma} = 0.1661]
Data/restraints/parameters	5807/54/360
Goodness-of-fit on F^2	0.873
Final R indexes [I>=2σ (I)]	R_1 = 0.0384, wR_2 = 0.0623
Final R indexes [all data]	R_1 = 0.0881, wR_2 = 0.0706
Largest diff. peak/hole / e Å^{-3}	1.81/-2.15

3.1.12 [(3-(2-Ethylbutyl)-1-(1H-κ*N*2-pyrazol-1-yl)-4-oxo-1,4-dihydronaphtalene-1,2-bis(olato)-κ*O*1-κ*O*2)(η^6-*p*-cymene)osmium(II)]: **2f (CCDC 2469755)**

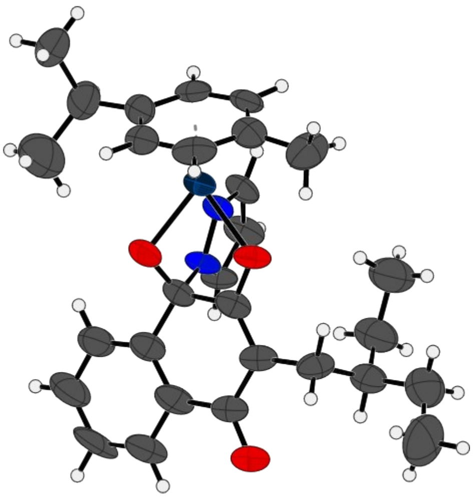

Figure S42: Crystal structure of complex **2f**.

A suitable crystal was selected and measured on a Bruker APEX-II CCD diffractometer. The crystal was kept at 200.15 K during data collection. Using Olex2, the structure was solved with the SHELXT structure solution program using Intrinsic Phasing.

Table 12: Sample and crystal data of **2f**.

**Empirical formula**	$C_{29}H_{34}N_2O_3Os$
**Formula weight**	648.78
**Temperature/K**	200.15
**Crystal system**	triclinic
**Space group**	P-1
**a/Å**	12.4011(16)
**b/Å**	15.266(2)
**c/Å**	16.009(2)
**α/°**	70.693(4)
**β/°**	81.979(4)
**γ/°**	69.726(4)
**Volume/Å³**	2682.0(6)

**Z**	4
**ρ_{calc}g/cm^3**	1.607
**μ/mm^{-1}**	4.786
**F(000)**	1288.0
**Crystal size/mm^3**	0.5 × 0.125 × 0.1
**Radiation**	MoKα (λ = 0.71073)
**2Θ range for data collection/°**	3.802 to 50.684
**Index ranges**	-14 ≤ h ≤ 14, -16 ≤ k ≤ 18, 19 ≤ l ≤ 0
**Reflections collected**	9392
**Independent reflections**	9392 [R_{int} = 0.0922, R_{sigma} = 0.0788]
**Data/restraints/parameters**	9392/344/714
**Goodness-of-fit on F^2**	1.057
**Final R indexes [I>=2σ (I)]**	R_1 = 0.0462, wR_2 = 0.0855
**Final R indexes [all data]**	R_1 = 0.0964, wR_2 = 0.0954
**Largest diff. peak/hole / e Å^{-3}**	1.08/-0.94

4 HPLC-MS

Compounds **1a–f**, **2a–f** were dissolved in DMSO (10 µM) and resulting stock solutions were diluted to final concentrations of 20 µM in phosphate buffered saline (pH 7.4) with 1% DMSO. Samples were stored at 20 °C and analyzed *via* UHPLC at time points 0, 1, 2, 3, 4, 5, 24 and 48 hours (5–95% acetonitrile in MQ water, consistent flow rates at 0.6 mL/min, acid free conditions, C18 column).

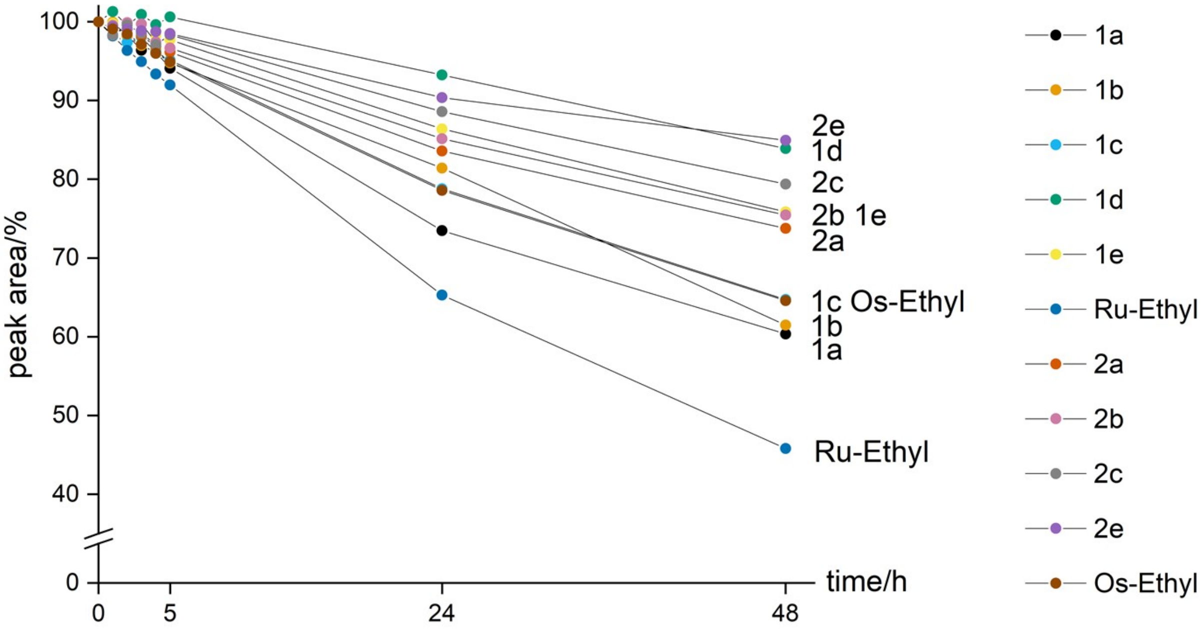

Figure S43: Stability in aqueous solution (PBS/1% DMSO) over 48 hours (A/A_0=area at given timepoint/area at timepoint 0).

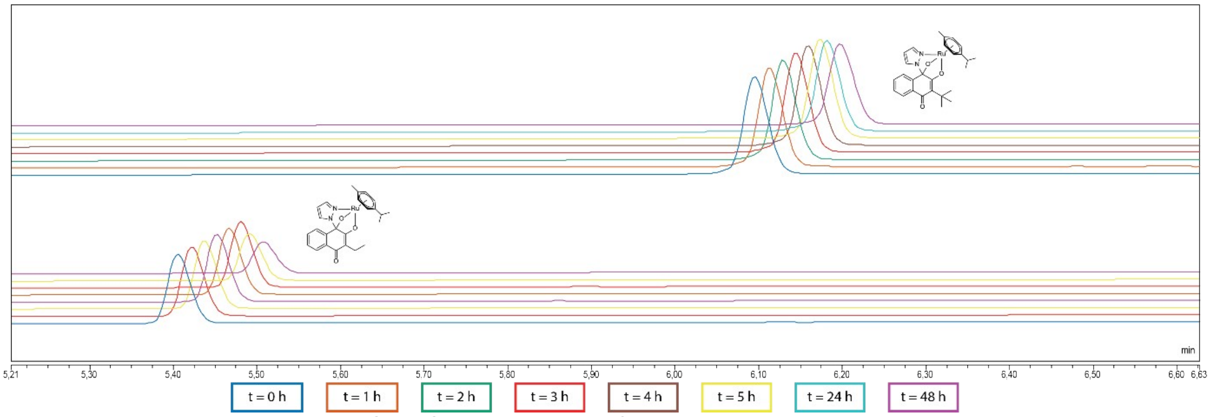

Figure S44: Chromatograms of Ru-Ethyl and **1d** over time.

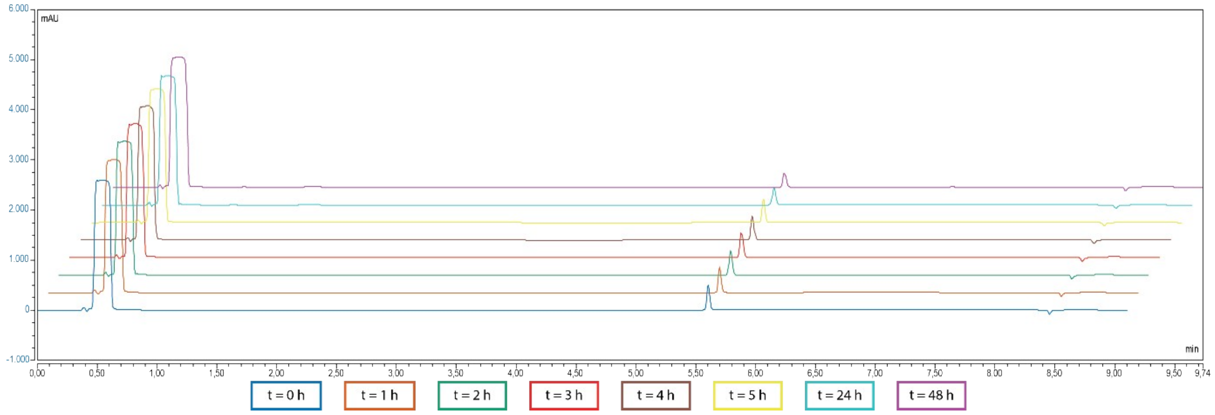

Figure S45: Chromatograms of complex **1a** over time.

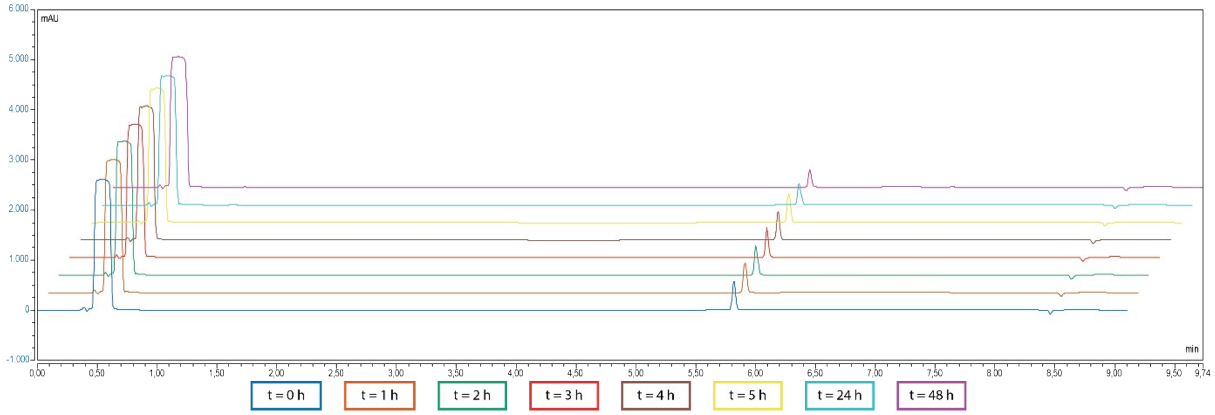

Figure S46: Chromatograms of complex **1b** over time.

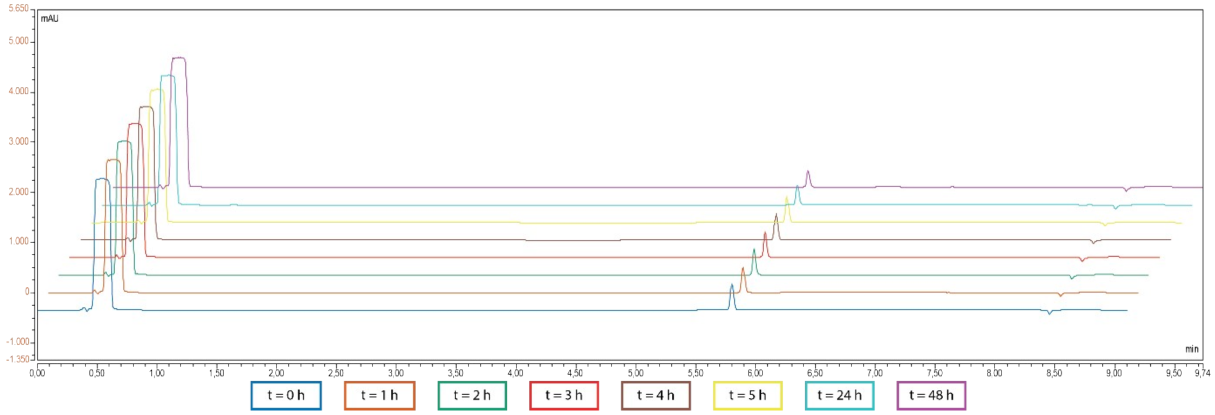

Figure S47: Chromatograms of complex **1c** over time.

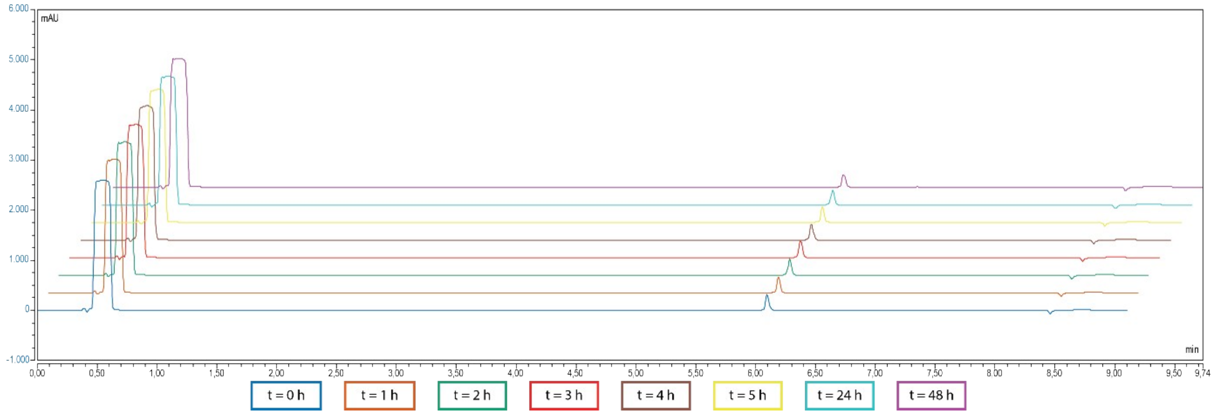

Figure S48: Chromatograms of complex **1d** over time.

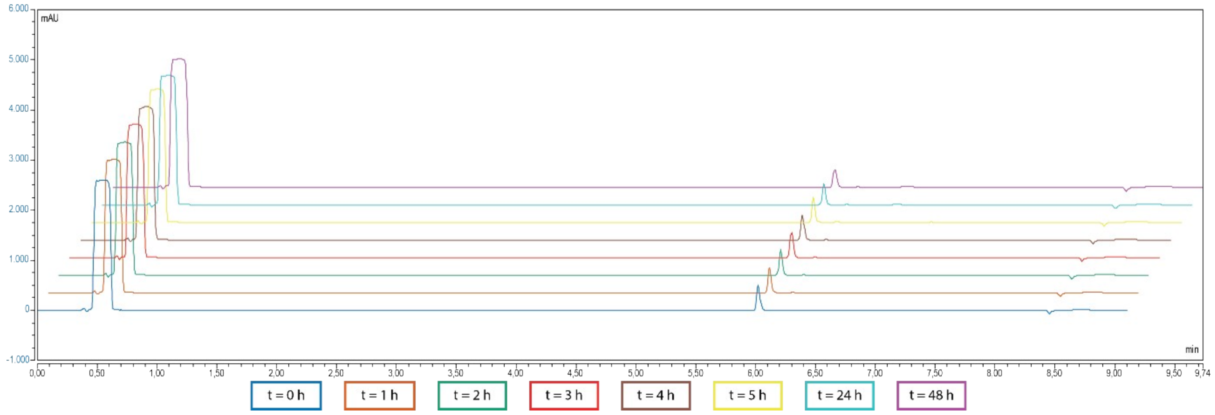

Figure S49: Chromatograms of complex **1e** over time.

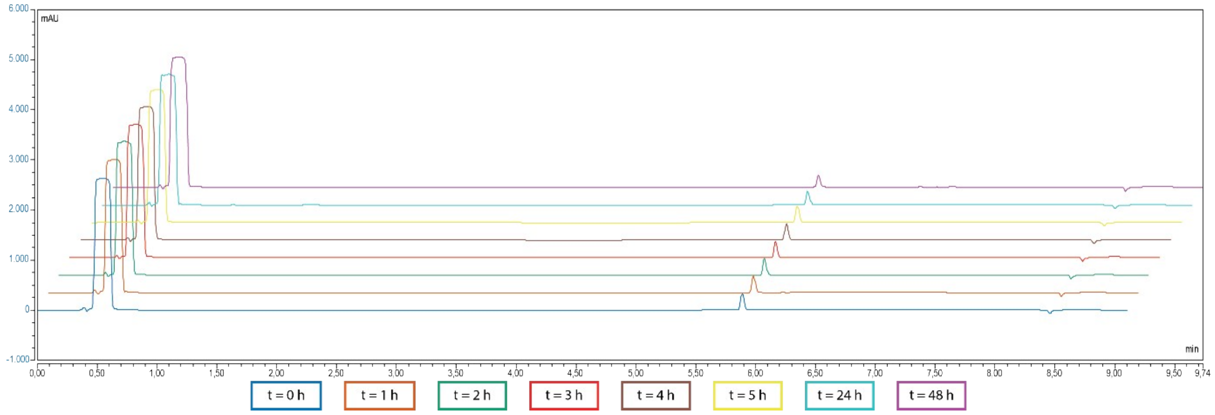

Figure S50:Chromatograms of complex **2a** over time.

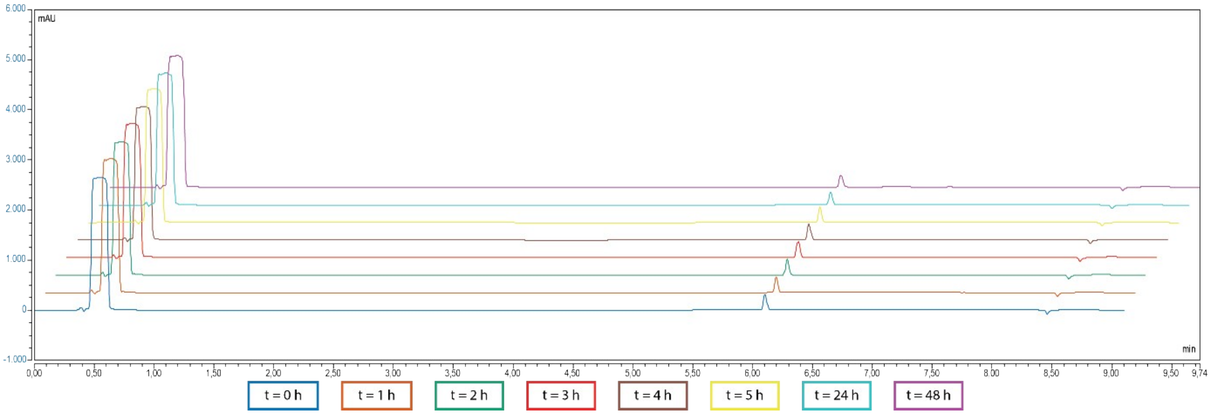

Figure S51: Chromatograms of complex **2b** over time.

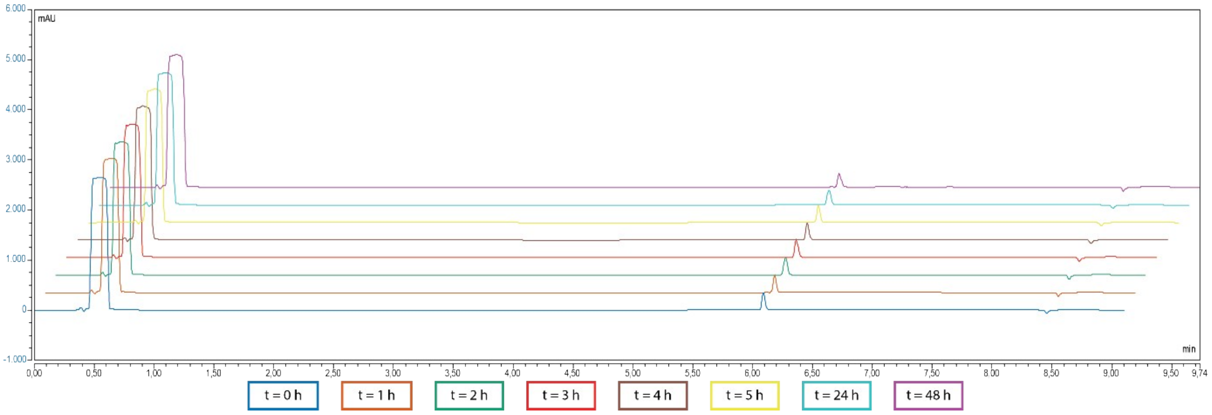

Figure S52: Chromatograms of complex **2c** over time.

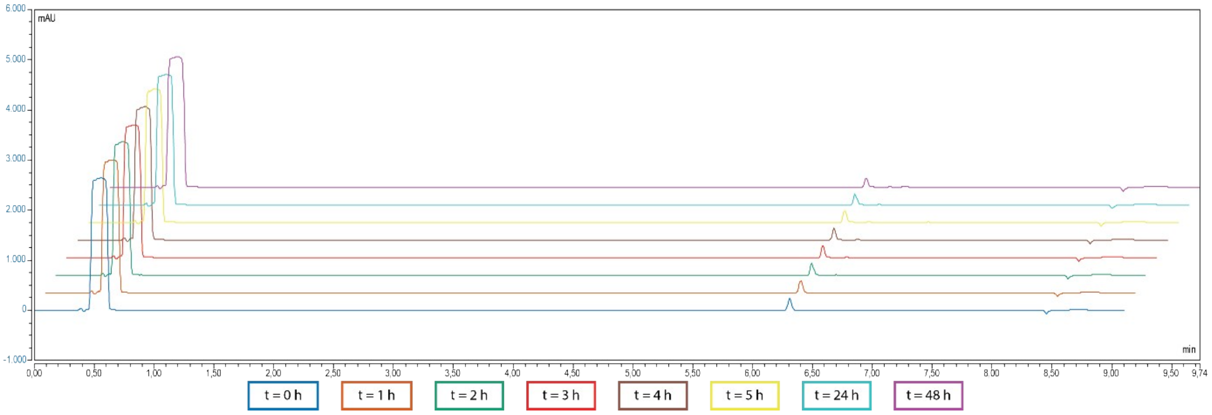

Figure S53: Chromatograms of complex **2e** over time.

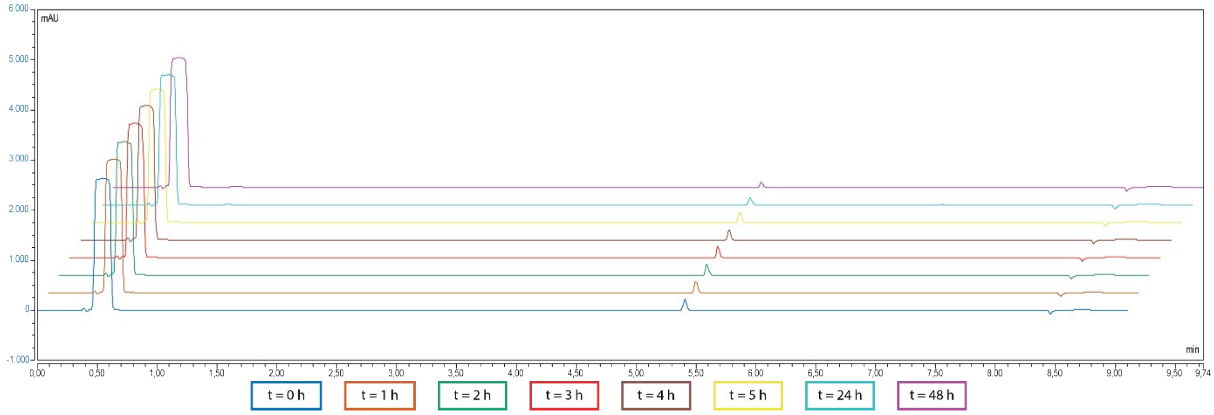

Figure S54: Chromatograms of Ru-Ethyl over time.

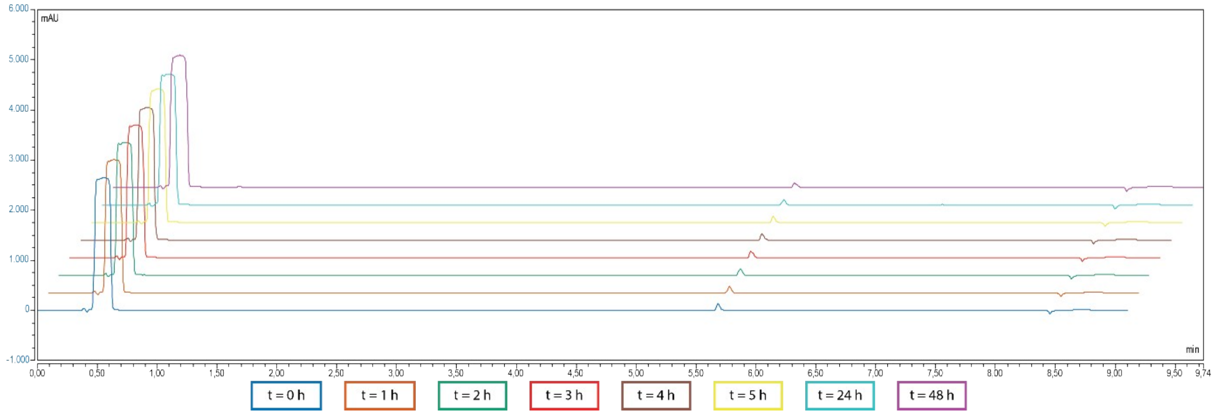

Figure S55: Chromatograms of Os-Ethyl over time.

5 Cell Culture

All cell lines were cultivated at 37°C, 5% CO_2, humidified atmosphere in 75 cm^2 culture flasks as monolayer cultures. The growth culture medium was Minimal Essential Medium (MEM) supplemented with 10% fetal bovine serum (FBS, Biowest), 1 mM sodium pyruvate solution, 1% non-essential amino acids solution (from 100× stock) and 4 mM L-glutamine. RPMI-1640 used in cell viability assay was supplemented with 10% FBS and 4 mM L-glutamine. All reagents were purchased from Sigma-Aldrich (Merck).

5.1 Cell Viability Assay (MTT Assay)

The cytotoxicity of KP2912 was determined on adherently-grown cell cultures via MTT assay (3-[4,5-dimethylthiazol-2-yl]-2,5-diphenyltetrazolium bromide). For this purpose, the cancer cells were seeded in 96-well plates (CytoOne) in cell densities of 3×10^3 cells/well for A549 (non-small cell lung carcinoma), 2×10^3 cells/well for SW480 (colon carcinoma) and 1×10^3 cells/well for PA-1/CH-1(ovarian teratocarcinoma) to ensure exponential growth during the entire experiment. Cells were allowed to recover and settle for 24 h prior to drug exposure. The complex was dissolved in DMSO/MEM in a way that the DMSO concentration on the cells did not exceed 0.5%. The compound dilutions were added to the cells in three technical replicates (100 µL/well), negative control wells were supplemented with complete media (100 µL/well). The plates were incubated for 96 h at 37°C, 5% CO_2, moist atmosphere. After incubation, the drug-containing medium was replaced with 100 µL of a 1:6 MTT-RPMI mixture (MTT solution: 5 mg/mL MTT in PBS) and incubated for 4 h. The yellow MTT-containing supernatant was removed and formazan-crystals formed in viable cells were dissolved in 150 µL of DMSO. After 15 min, the optical densities were measured with a microplate reader (ELx808, Biotek) at a wavelength of 550 nm and a reference wavelength of 690 nm. The IC_{50} values were interpolated from concentration-effect curves. The calculations are based on at least three independently performed experiments, each with triplicates per concentration level.

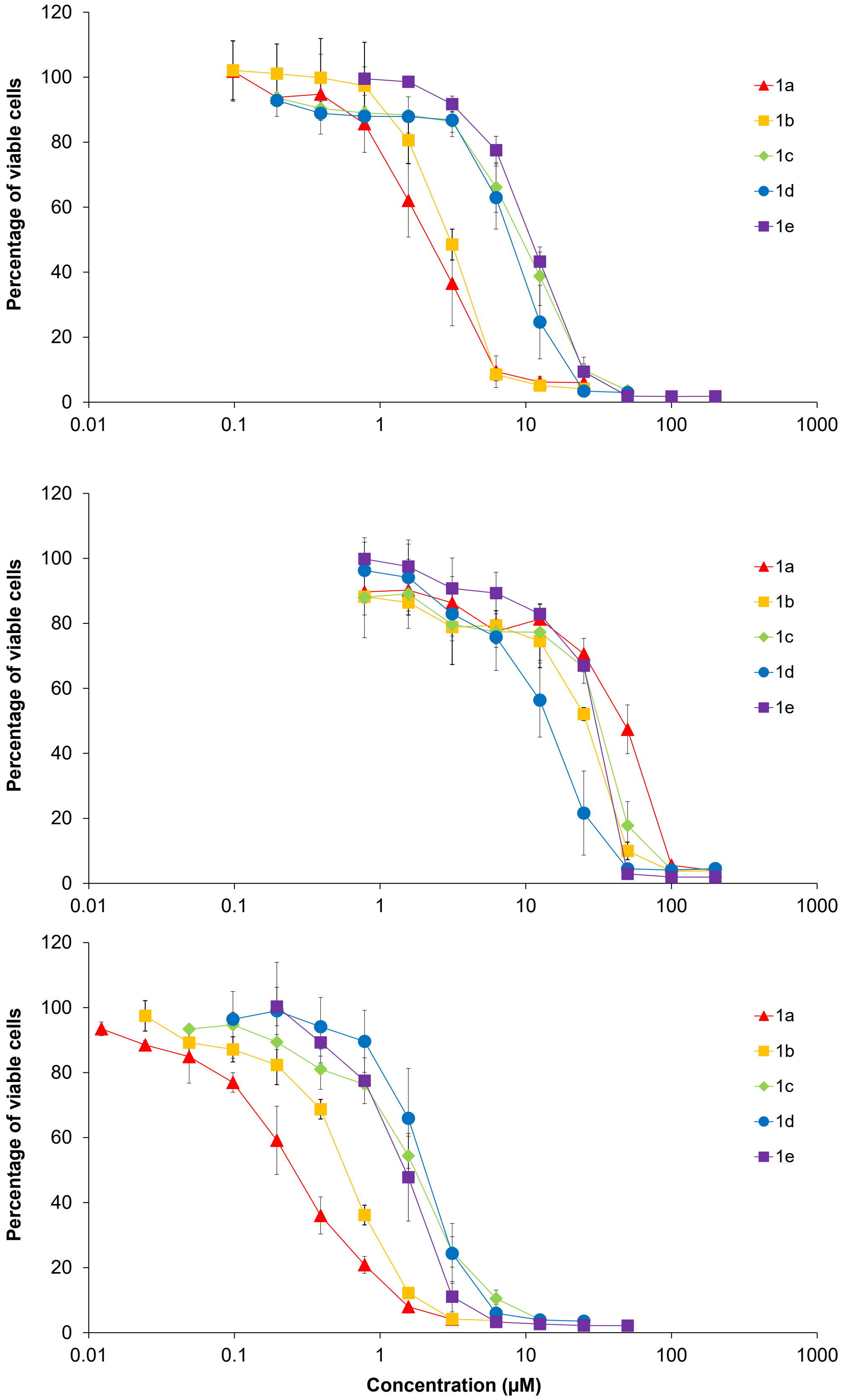

Figure S56: Concentration-effect curves of 1a–1e in A549 (top), CH1/PA-1 (center) and SW480 cells (bottom) relative to untreated controls (100%). Values are means ± standard deviations from at least three independent 96 h MTT assays.

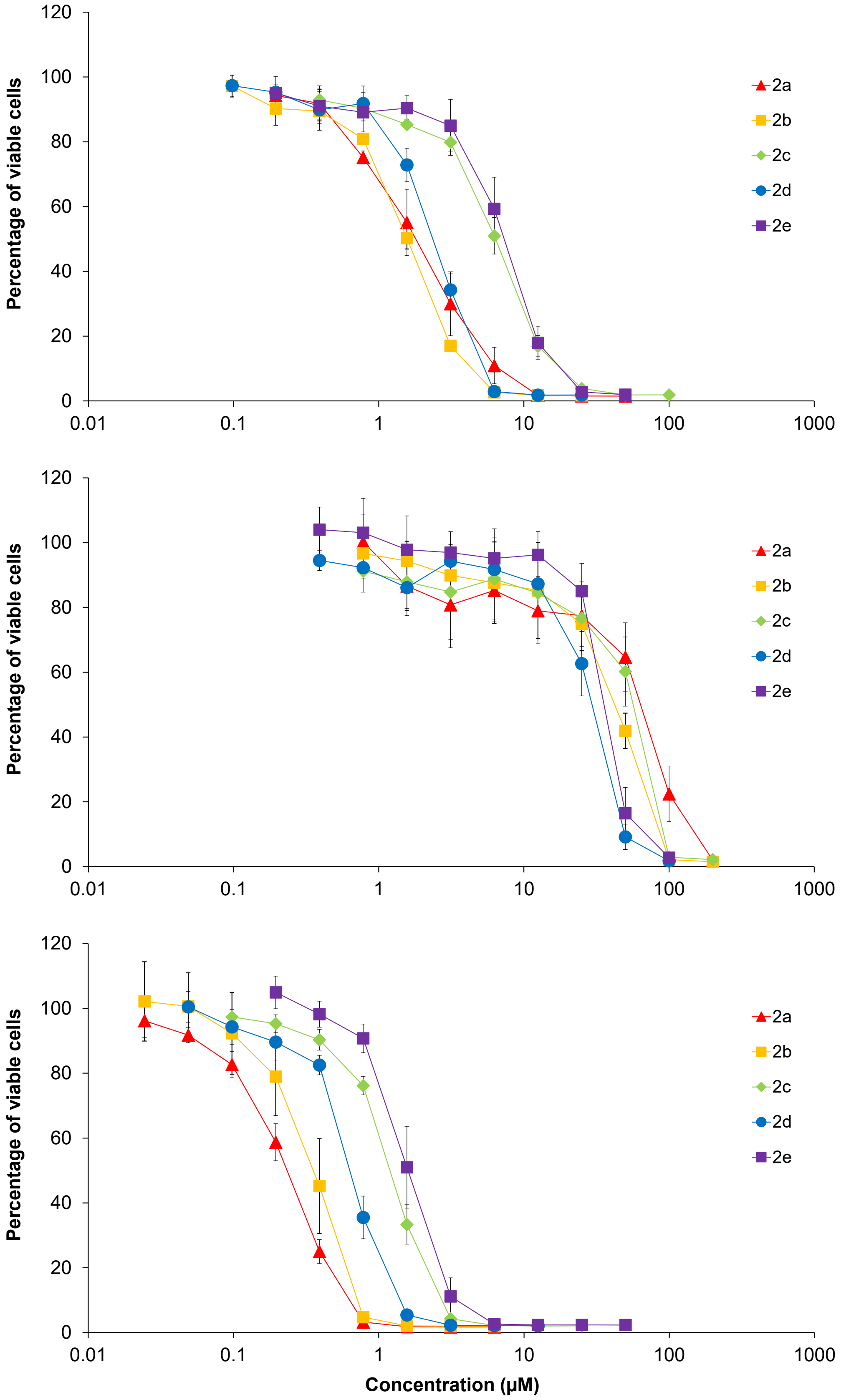

Figure S57: Concentration-effect curves of **2a**–**2e** in A549 (top), CH1/PA-1 (center) and SW480 cells (bottom) relative to untreated controls (100%). Values are means ± standard deviations from at least three independent 96 h MTT assays.

5.2 Cellular accumulation

Table S13: Accumulation in SW480 cells. It was examined for ruthenium derivatives 1a–1e and Ru-Ethyl. Solvent: 0.5% DMSO.

Compound	Substituent	Concentration	Average	Standard deviation	
		µM	fg Ru/cell	fg Ru/cell	RSD/%
**Ru-Ethyl**	C_2H_5	50	545.9	15.0	3
**1a**	C_3H_7	50	362.4	68.3	19
**1b**	C_4H_9	50	242.6	58.6	24
**1c**	C_4H_9	50	109.8	4.6	4
**1d**	C_4H_9	50	78.2	20.9	27
**1e**	C_5H_{11}	50	72.9	6.1	8

5.3 Cell Cycle Studies

For cell cycle studies the colon carcinoma cells (SW480) and ovarian teratocarcinoma cells (PA-1/CH-1) were seeded in 12-well plates (CytoOne) in densities of 1×10^5 cells per well (in 1 mL MEM). After the recovery time of 24 h, cells were treated with different concentrations of the organoruthenium and organoosmium test compounds Ru-Ethyl, Os-Ethyl, **1a**, **1c**, **2a**, **2c**. For this purpose, the test substances were dissolved in DMSO/MEM in a way that the maximum concentration of DMSO on the cells did not exceed 0.5%. Etoposide (causes G2/S phase arrest) and gemcitabine (causes G1/S arrest) were applied as positive controls. Plates were incubated at 37°C, 5% CO_2, moist atmosphere for 24 h. Following the exposure, cells were collected via trypsinisation, washed with complete MEM and PBS and stained with DNA-intercalating dye propidium iodide (PI) diluted in hypotonic fluorochrome solution (HFS: 0.1% (V/V) Triton X-100; 0.1% (W/V) sodium citrate in H_2O, PI/HFS mixture: 40 µg/mL, 500 µL per probe). The PI solution was added to the HFS shortly before use and the cells were stained in the dark at 4 °C overnight. The fluorescence of all samples was measured no longer than 24 h after staining procedure by means of flow cytometry (Guava easyCyte 8HT, Millipore).

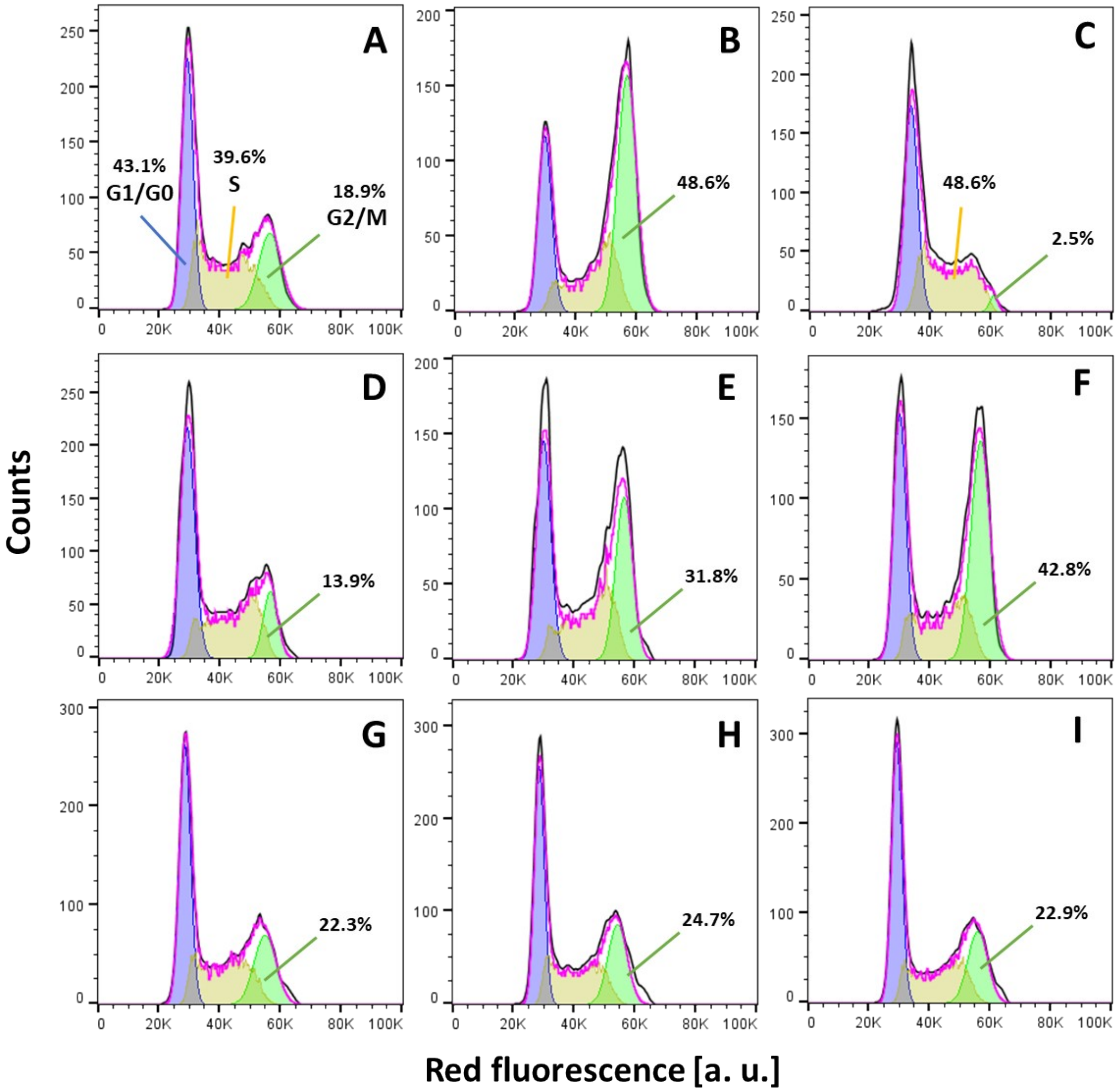

Figure S58: Histograms of cell cycle phase distribution in PA-1 ovarian cancer cells. Negative control (A), etoposide – G2/M phase inhibitor (B), gemcitabine – S phase inhibitor (C), raising concentrations of Ru-Ethyl (D-F, 0.05 μM, 25 μM, 50 μM), raising concentrations of Os-Ethyl (G-I, 0.05 μM, 25 μM, 50 μM).

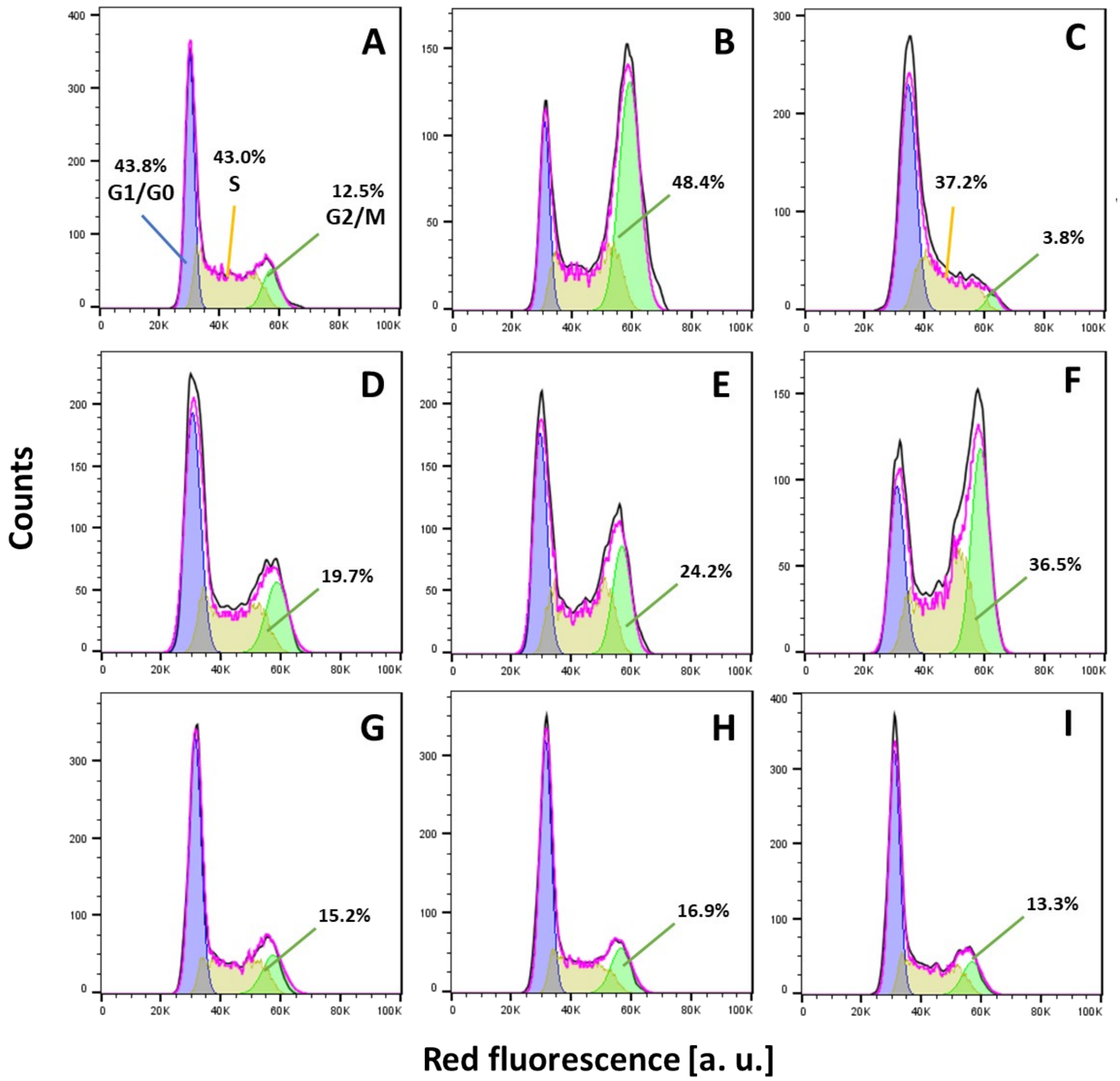

Figure S59: Histograms of cell cycle phase distribution in SW480 colon cancer cells. Negative control (A), etoposide – G2/M phase inhibitor (B), gemcitabine – S phase inhibitor (C), raising concentrations of Ru-Ethyl (D-F, 0.0125 μM, 0.025 μM, 0.05 μM), raising concentrations of Os-Ethyl (G-I, 0.0125 μM, 0.025 μM, 0.05 μM).

5.4 Colorimetric Kyn Assay.

SKOV3 cells were seeded at 4000 cells/well in 96-well plates and allowed to recover for 24 h. Cells were treated for 72 h. For the post-washout experiments, supernatants (150 µL/well) and blank medium were transferred to microcentrifuge tubes. Confluency was measured by Incucyte S3. To precipitate proteins, the supernatants were mixed with 75 µL 30% (w/v) trichloroacetic acid. N-Formyl-Kyn was hydrolyzed to Kyn by incubating the supernatants for 30 min in a thermoblock at 50 °C and 300 rpm. The supernatants were cleared by centrifugation (10 min at 10,000g), and 100 µL of the clear supernatants was transferred to a fresh 96-well plate in duplicates and incubated with 100 µL Ehrlich's reagent [2% 4-(dimethylamino)-benzaldehyde in acetic acid] for 10 min at r.t.. The absorption of Kyn was measured at 490 nm (reference 620 nm), and the absorbance of blank medium was subtracted. The Kyn concentrations were calculated using a standard curve [generated from L-Kyn dilution series in blank medium] and normalized to confluency.

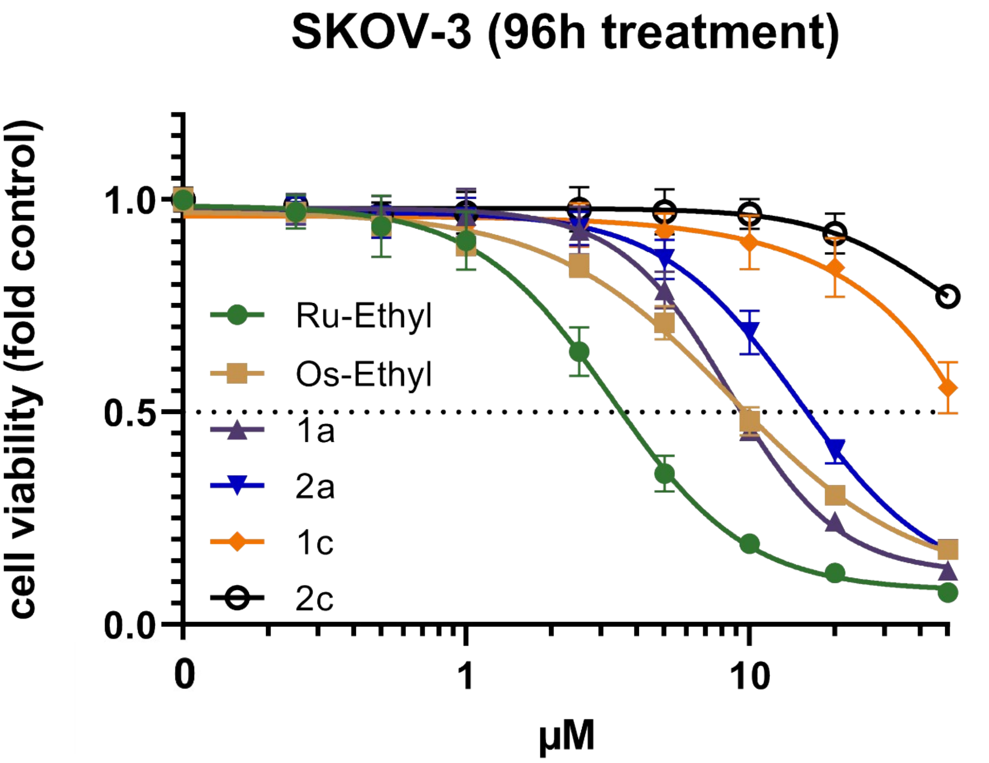

Figure S60: Concentration-effect curves of Ru-Ethyl, Os-Ethyl, **1a**, **2a**, **1c**, **2c** in SKOV3 relative to untreated controls (100%). Values are means ± standard deviations from at least three independent MTT assays.

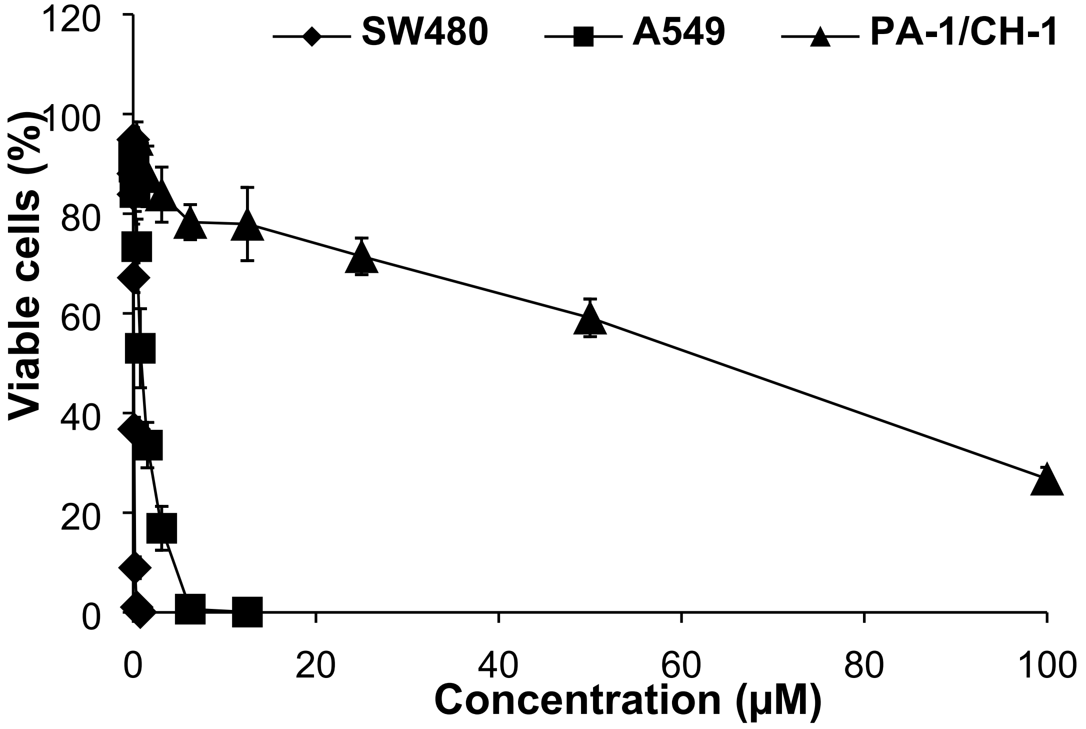

Figure S61: Concentration-effect curves of Os-Ethyl upon treatment of three human cancer cell lines (curves left to right: SW480, A549, CH1/PA-1).

Table S14: Cell cycle phase distribution in CH1/PA-1 cells upon treatment with test-compounds. Values are presented as means ± standard deviations calculated from at least 3 independent experiments. Ru-Ethyl, **1a**, **1c**, Os-Ethyl, **2a** and **2c** were analyzed.

Ruthenium

		Cell cycle phase distribution, %		
Compound	Concentration, µM	G1/G0	S	G2/M
Negative control		37 ± 3	42 ± 5	22 ± 4
Etoposide	0.1	21 ± 4	36 ± 8	45 ± 8
Gemcitabine	0.01	54 ± 4	45 ± 3	2 ± 1
Ru-Ethyl	0.05	37 ± 14	50 ± 10	16 ± 4
	25	31 ± 7	42 ± 7	29 ± 8
	50	29 ± 7	37 ± 9	36 ± 9
**1a**	0.25	41 ± 6	39 ± 4	22 ± 3
	25	29 ± 8	43 ± 10	30 ± 8
	50	30 ± 4	39 ± 7	33 ± 10
**1c**	2	39 ± 8	39 ± 9	23 ± 6
	12.5	31 ± 9	45 ± 12	27 ± 5
	25	32 ± 4	41 ± 11	29 ± 10

Osmium

		Cell cycle phase distribution, %		
Compound	Concentration, µM	G1/G0	S	G2/M
Negative control		43 ± 5	42 ± 2	18 ± 4
Etoposide	0.1	28 ± 6	31 ± 9	45 ± 13
Gemcitabine	0.01	53 ± 8	44 ± 4	4 ± 1
Os-Ethyl	0.05	45 ± 6	41 ± 5	17 ± 5
	25	40 ± 2	45 ± 9	16 ± 7
	50	43 ± 3	38 ± 6	22 ± 6
**2a**	0.25	40 ± 4	46 ± 5	15 ± 4
	25	42 ± 5	39 ± 6	21 ± 6
	50	42 ± 3	37 ± 3	23 ± 3
**2c**	2	42 ± 4	41 ± 4	19 ± 4
	12.5	42 ± 2	39 ± 7	20 ± 5
	25	42 ± 5	39 ± 6	21 ± 5

Table S15: Cell cycle phase distribution in SW480 cells upon treatment with test-compounds. Values are presented as means ± standard deviations calculated from at least 3 independent experiments. Ru-Ethyl, **1a**, **1c**, Os-Ethyl, **2a** and **2c** were analyzed.

Ruthenium

Compound	Concentration, µM	Cell cycle phase distribution, % G1/G0	S	G2/M
Negative control		48 ± 7	38 ± 5	14 ± 2
Etoposide	0.1	29 ± 8	35 ± 4	37 ± 10
Gemcitabine	0.01	57 ± 5	41 ± 6	3 ± 1
Ru-Ethyl	0.0125	38 ± 10	39 ± 4	25 ± 5
	0.025	28 ± 8	43 ± 4	30 ± 6
	0.05	17 ± 7	50 ± 8	35 ± 2
**1a**	0.063	31 ± 6	46 ± 6	26 ± 1
	0.125	20 ± 5	51 ± 9	29 ± 6
	0.25	17 ± 3	53 ± 7	31 ± 10
**1c**	0.5	43 ± 6	42 ± 4	19 ± 4
	1	34 ± 7	44 ± 8	25 ± 4
	2	22 ± 6	50 ± 9	31 ± 5

Osmium

Compound	Concentration, µM	Cell cycle phase distribution, % G1/G0	S	G2/M
Negative control		52 ± 6	37 ± 4	15 ± 1
Etoposide	0.1	35 ± 5	40 ± 10	28 ± 8
Gemcitabine	0.01	62 ± 9	37 ± 9	3 ± 2
Os-Ethyl	0.0125	51 ± 4	38 ± 4	15 ± 2
	0.025	50 ± 6	37 ± 6	15 ± 2
	0.05	50 ± 3	39 ± 4	15 ± 2
**2a**	0.063	49 ± 4	40 ± 7	15 ± 4
	0.125	49 ± 4	37 ± 6	16 ± 2
	0.25	50 ± 4	35 ± 7	19 ± 2
**2c**	0.25	49 ± 4	38 ± 7	17 ± 2
	0.5	50 ± 4	38 ± 4	16 ± 4
	1	52 ± 4	36 ± 5	17 ± 6

SW480 cells | CH1/PA-1 cells

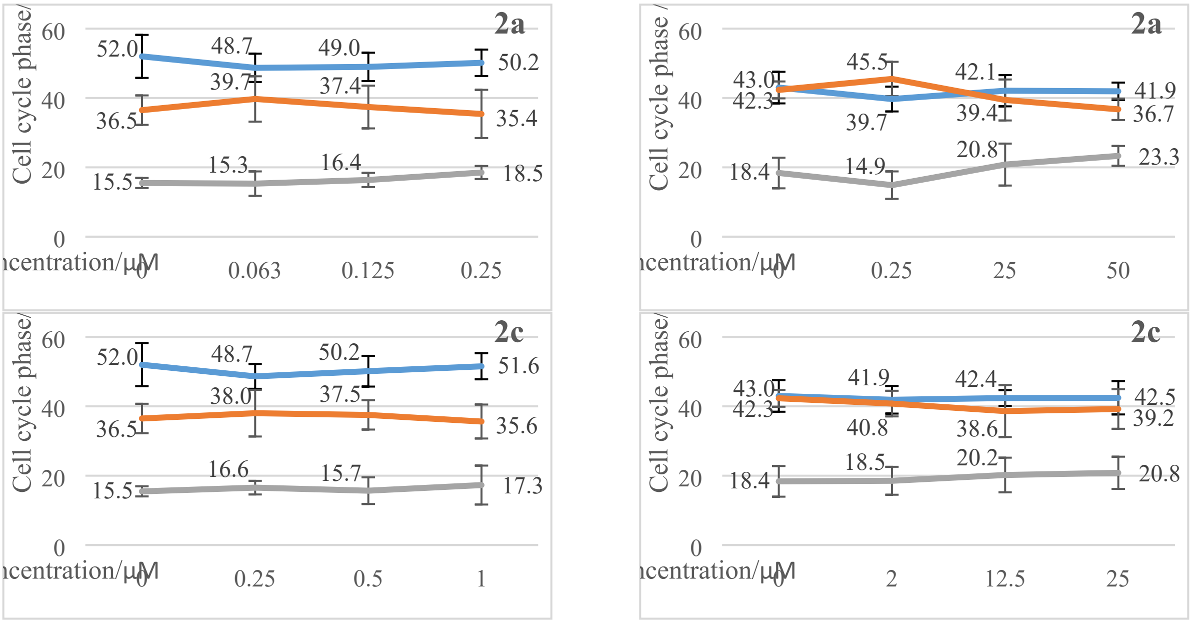

Figure S621: Cell cycle distribution in SW480 colon cancer cells (left) and CH1/PA-1 ovarian cancer (right) cells upon 24 h exposure osmium complexes **2a** and **2c**.

SW480 cells | CH1/PA-1 cells

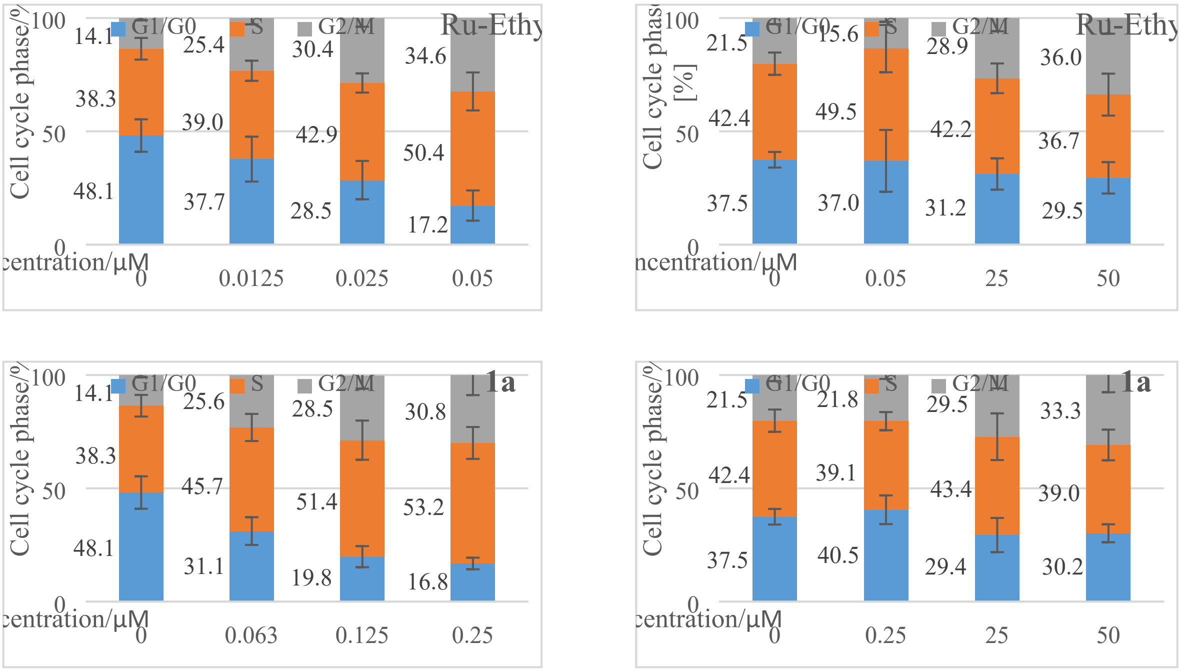

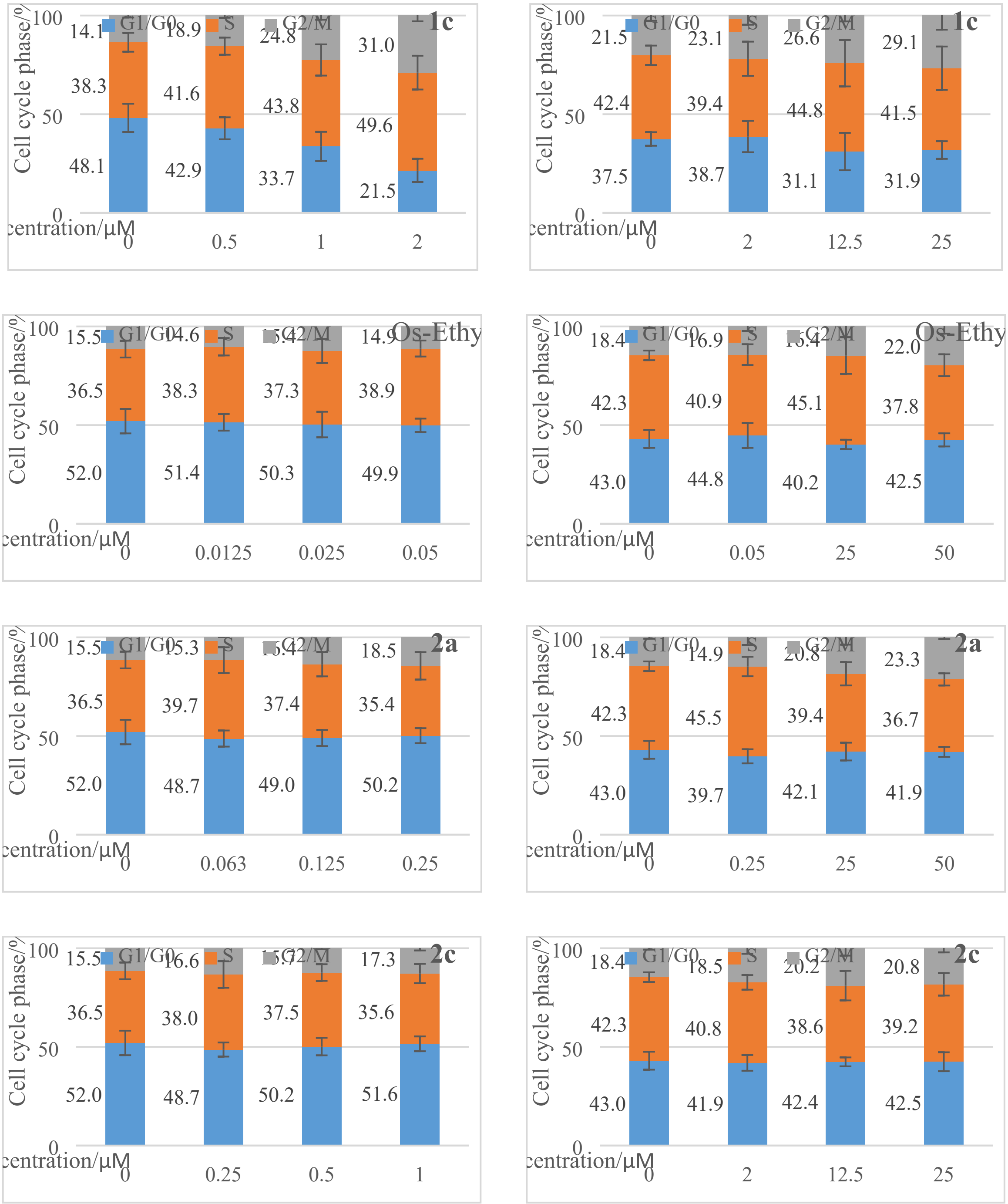

Figure S63: Cell cycle distribution in SW480 colon cancer cells (left) and CH1/PA-1 ovarian cancer (right) cells upon 24 h exposure of complexes Ru-Ethyl, **1a**, **1c**, Os-Ethyl, **2a** and **2c**.
